# Assessment of a Teaching Module for Cardiac Auscultation of Horses by Veterinary Students

**DOI:** 10.3390/ani14091341

**Published:** 2024-04-29

**Authors:** Alyse Wood, Frances Marie Shapter, Allison J. Stewart

**Affiliations:** School of Veterinary Science, University of Queensland Gatton, 5391 Warrego Highway, Gatton, QLD 4343, Australia; alyse.wood@uqconnect.edu.au (A.W.); f.shapter@uq.edu.au (F.M.S.)

**Keywords:** self-directed learning, equine, education, COVID-19, on-line learning, cardiology, murmur, arrhythmia, electronic stethoscope, thematic analysis

## Abstract

**Simple Summary:**

Veterinary students are required to determine an animal’s heart rate and diagnose heart murmurs and arrhythmias by the time of graduation. Accurate assessment of the heart sounds of horses is considered as a day one clinical competency. Limited opportunities exist in the veterinary curriculum to develop these competencies, especially during COVID-19 times. This research aimed to determine if a multimodal learning resource consisting of diagrams, heart sound recordings and visual representation of the sounds would assist in the development of veterinary students’ confidence and ability to recognize normal and abnormal heart sounds of horses. Students were invited to utilize the teaching resource and voluntarily complete surveys about their confidence in recognizing normal heart sounds as well as various murmurs and important arrhythmias of horses. The survey results were analyzed by qualitative and quantitative means. Over a two-year period, 231 fourth-year and 222 fifth-year veterinary students had access to the resource; 89 completed the initial survey and 57 completed a second survey after using the resource. Results confirmed that after access to the resource students’ understanding and perception of their auscultation abilities improved.

**Abstract:**

Auscultation of heart sounds is an important veterinary skill requiring an understanding of anatomy, physiology, pathophysiology and pattern recognition. This cross-sectional study was developed to evaluate a targeted, audio-visual training resource for veterinary students to improve their understanding and auscultation of common heart conditions in horses. Fourth- and fifth-year 2021 and 2022 Bachelor of Veterinary Science students at the University of Queensland (UQ) were provided the learning resource and surveyed via online pre- and post-intervention surveys. Results were quantitatively analyzed using descriptive statistics and Mann–Whitney U tests. Open-ended survey questions were qualitatively analyzed by thematic analysis and Leximancer™ Version 4 program software analysis. Over the two-year period, 231 fourth-year and 222 fifth-year veterinary students had access to the resource; 89 completed the pre-intervention survey and 57 completed the post-intervention survey. Quantitative results showed the resource helped students prepare for practicals and their perception of competency and confidence when auscultating equine cardiac sounds improved (*p* < 0.05). Compared to fifth-year students, fourth-year students felt less competent at identifying murmurs and arrythmias prior to accessing the learning resource (*p* < 0.05). Fourth-year and fifth-year students’ familiarity with detection of murmurs improved after completing the learning resource (*p* < 0.001). Qualitative analysis demonstrated a limited number of opportunities to practice equine cardiac auscultation throughout the veterinary degree, especially during the COVID-19 pandemic, and that integrated audio-visual resources are an effective means of teaching auscultation.

## 1. Introduction

The development of strong practical skills is an important component of veterinary medicine [1,2,3,4]. Skills training is optimized by a multidimensional approach, which creates a safe environment for active learning, provides students with a scaffold to guide their self-directed learning (SDL), encourages students to self-assess their development, is relevant and facilitates students taking responsibility for their learning and tailoring it to their individual strengths and weaknesses [2,5,6,7,8]. A recent study conducted by Muca et al. (2023) also determined that flipped classroom (FC) and peer-assisted learning methods helped veterinary students engage more in nutritional classes [9]. The flipped classroom technique incorporates self-directed learning components that enhance a student’s engagement and willingness to learn, and use time more effectively in class [9,10].

Due to costs, safety and animal welfare considerations, during a five-year Bachelor of Veterinary Science degree, opportunities to practice equine auscultatory skills are finite, with the bulk of equine auscultations occurring during final year rotations. Students obtain cardiac auscultation experiences across many species, which supports comparative learning between, and cumulative learning across, all species taught. However, while most auscultation skills are transferable between species, some aspects of diagnostic equine auscultation are specific to horses. The conventional approach to learning equine cardiac auscultation is through the delivery of lecture content, practical sessions and participating in rotations involving client-owned animals. UQ students’ first exposure to cardiac anatomy and physiology content is in the first semester of their second year. This is followed by physical examination practicals in their third year, where students practice multi-species heart auscultation, including horses. In the first semester of their fourth year, equine cardiology theory is reviewed, followed by a forty-minute practical in the second semester focusing on equine cardiac auscultation. Finally, in their fifth year, students spend a minimum of one month on an equine rotation and have the option to competitively apply to enroll in an additional four-week rotation in the specialist equine hospital. However, in 2020 and 2021 the third- and fourth-year practical classes, as well as fifth-year rotations, were severely impacted by the COVID-19 pandemic, requiring the addition of new supporting resources to bridge the gaps in face-to-face teaching. The 2020 to 2022 graduating cohorts may have only been exposed to live auscultation of the equine heart during their final year rotations. Across many disciplines, graduates whose practical education was affected by the COVID-19 pandemic teaching constraints are cognizant of perceived and actual deficiencies in their learning. 

Veterinary licensure allows a veterinarian to treat any patient within their capabilities. However, it is difficult for students and new graduates to determine what they do not know, and if physical examination competencies are underdeveloped, then the health of the patient may suffer [11,12]. Veterinary and medical educators agree that students benefit from a multidimensional approach to teaching auscultation techniques [4,13,14]. Therefore, it is paramount that during their training, students receive as many varied learning and self-assessment opportunities as possible to improve their competency and self-efficacy prior to graduation. UQ students who have an interest in equine or mixed practice can become involved in a student led Equine Special Interest Group, assist with an annual equine practitioner’s conference and can complete two to four weeks of additional elective rotations involving horses. Additionally, many students appreciate self-directed learning (SDL) opportunities to increase their skills, especially when the availability of practical sessions or clinical rotations is curtailed, as occurred during the COVID-19 pandemic. 

**Figure 1:**
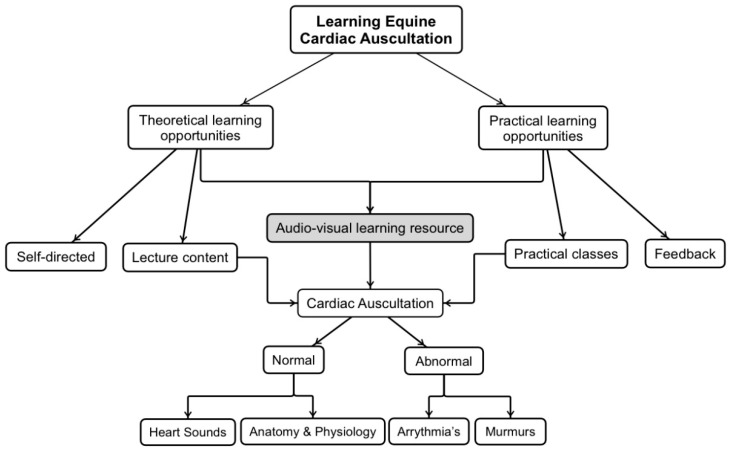
Equine cardiac auscultation learning pathway for veterinary science students at the University of Queensland.

When creating SDL resources for auscultation skills, electronic stethoscopes allow recordings of heart, lung and gastrointestinal (GIT) sounds and their graphical representation (phonograph) to be saved as media files that can be incorporated into digital training resources [15]. Electronic stethoscopes allow for the recording of heart, lung and gastrointestinal (GIT) sounds and their graphical representation (phonograph). The sounds and phonographs can be saved as media files that can be incorporated into training resources. Some studies have shown that pairing sounds from electronic stethoscopes, digitally or in real-time, with phonographs assists with the identification of heart sounds to a greater degree than using conventional stethoscopes [13,16]. Integration of multidimensional teaching tools also improves animal health and well-being, as the students are better prepared during live animal interactions [1,17,18]. Hence an audio-visual equine cardiac auscultation resource was developed for veterinary students at UQ to integrate theoretical and practical content (Figure 1).

This study was designed to determine whether there would be an uptake of an additional learning SDL tool by already time-poor students, and whether the resource aided in the development of students’ perceived equine cardiac auscultatory confidence and improved practical training of fourth- and final-year veterinary students at UQ. Feedback was collected from pre- and post-intervention surveys, which is essential to understand the value of further development of multimodal learning resources and models for veterinary students nationally and internationally. 

## 2. Methodology

### 2.1. Live Animal Recordings

This study was conducted in compliance with the animal ethics guidelines (certificate number SVS/214/20). Thirty-five adult horses (University of Queensland Australian Stock Horse Stud and client owned), ten foals/juvenile (University of Queensland Australian Stock Horse Stud) and six client-owned horses with cardiac pathologies (with written consent) had their heart sounds recorded utilizing the Eko CORE™ (Oakland, CA, USA) digital stethoscope. Heart sounds were recorded on the left side over the pulmonary (3rd intercostal space), aortic (4th intercostal space) and mitral valves (5th intercostal space), as well as on the right side over the tricuspid valve (4th intercostal space). Recordings were made under field conditions.

### 2.2. Resource Development and Distribution

**Figure 2:**
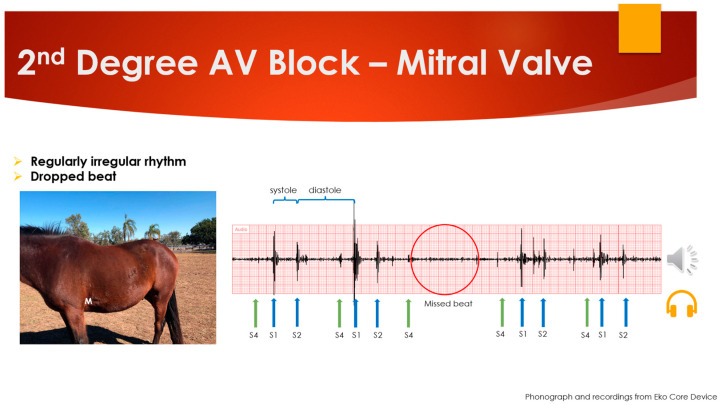
Example slide from the audio-visual resource (PowerPoint™ slide 53).

Sound recordings and phonographs of normal and abnormal auscultatory findings were downloaded from the Eko CORE™ Digital Stethoscope application (App, https://app.ekodevices.com (accessed on the 2 December 2019)) and uploaded to a cloud-based data manager. Normal and abnormal heart auscultatory findings were selected to create a PowerPoint™ (Microsoft, Redmond, WA, USA) learning resource. The resource included recordings of normal heart sounds, sounds with background interference from other internal sounds (gastrointestinal sounds) and interference from external sources (tractors, wild birds). Examples of classic equine cardiac abnormalities that were utilized as recordings included second-degree atrioventricular blocks, atrial fibrillation and murmurs (mitral, aortic and tricuspid, ventricular septal defect and pentalogy of Fallot). An example of the visual presentation of slides can be seen in Figure 2 and the full presentation can be viewed in Supplementary File S1. 

### 2.3. Survey Data Capture

The usefulness of the resource was assessed using qualitative means and quantitative statistics based on pre- and post-intervention surveys, which included an assessment of pre- and post-perceived knowledge (Supplementary File S2). Utilizing the PowerPoint™ presentation learning resource was not a curriculum requirement; however, all fourth- and fifth-year veterinary students were encouraged to access it via the online learning management system (Blackboard™; Reston, VI, USA). With ethical approval (project number 2020/HE002986), students who engaged with the learning resource were given the additional option of undertaking online, anonymous, pre- and post-intervention surveys. Human ethics required that students could utilize the resource without completing the surveys as it would likely enhance the grades of all students who did use the resource. All student responses had to be kept anonymous and lecturers that graded the students were not allowed to directly determine which students had utilized the resource. Therefore, it was impossible to test if student utilization of the resource directly improved the students’ grades or abilities. 

Overarching survey questions focused on establishing background information, the individual’s diagnostic capabilities and the importance of targeted, audio-visual training resources in the development of students’ practical skills in the auscultation of heart sounds. Surveys captured mainly dichotomized or ordinal responses (e.g., Likert scale or yes/no questions). Students were encouraged to complete the surveys for future study and resource purposes; however, students didn’t need to complete either survey due to human ethics constraints. 

### 2.4. Method of Data Analysis

Responses from the anonymous, pre- and post-intervention surveys were collated in Microsoft Excel™ (Microsoft, Redmond, WA, USA) spreadsheets and GraphPad Prism™ (GraphPad Software Version 10, San Diego, CA, USA). The Likert-style questions (Supplementary File S2) of both surveys were coded (strongly agree = 5, agree = 4, neutral = 3, disagree = 2, strongly disagree = 1). Simple descriptive statistics such as the number of respondents, the mean, standard deviation, median, inter-quartile ranges and confidence intervals (as appropriate for parametric and non-parametric data) were calculated for the coded responses of the pooled, fourth-year and fifth-year responses, on both software platforms for both surveys. The normality of distribution was assessed using the Shapiro–Wilk test on GraphPad Prism™. As all the data was non-parametric, GraphPad Prism™ was utilized to perform a Mann–Whitney U test on questions 8 to 14 of the pooled data comparing both surveys and comparing the fourth- and fifth-year responses for each question. The *p* value was set at <0.05 [19]. 

Qualitative analysis of the open-ended pre- and post-intervention survey questions was conducted by manual thematic analysis, the findings of which were compared with those generated by the Leximancer™ Version 4 software data analysis program (Brisbane, Queensland, Australia). The first step of the manual analysis was to examine the raw data and inductively develop a set of codes to represent patterns in the data (themes) [20,21]. This ‘coding template’ was utilized to code each response individually [20]. Once coded, key themes were identified based on salience to the research project and how prevalent they were across the dataset [20]. Leximancer™, a qualitative analysis software platform that statistically analyses a document’s text for keywords and concepts through the use of algorithms then displays these in a visual concept map without subjectivity and bias when qualitatively analyzing the data set [22,23,24,25]. The comparison of manually-coded and Leximancer analyses constitutes a form of triangulation and strengthens the reliability of this study’s qualitative findings [22,26,27].

## 3. Results

### 3.1. Quantitative Data 

**Figure 3:**
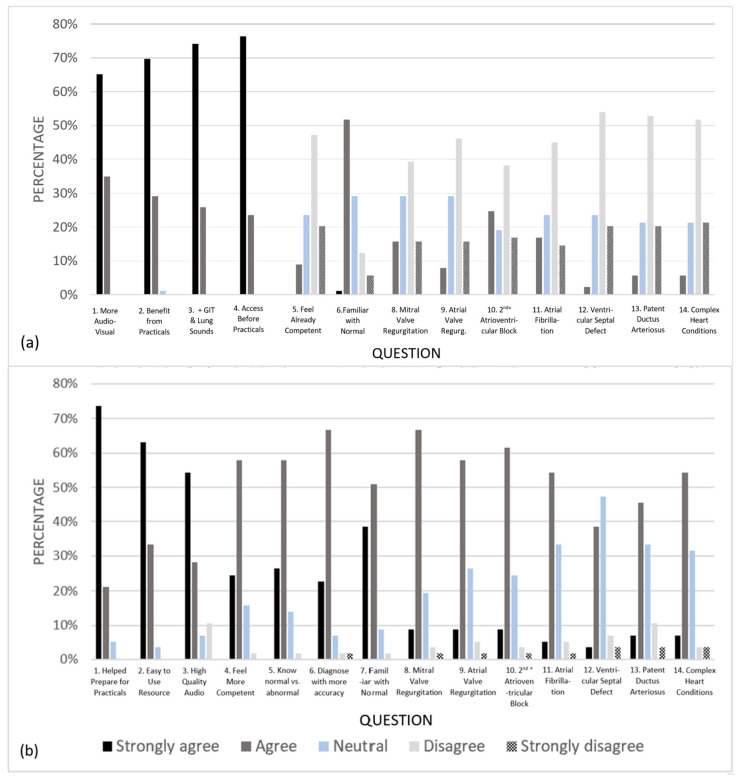
Response rates for the audio-visual auscultation resource surveys comparing pooled data for pre- and post-survey questions; (**a**) Pre-intervention survey Likert response rates, (**b**) post-intervention survey Likert response rates.

**Figure 4:**
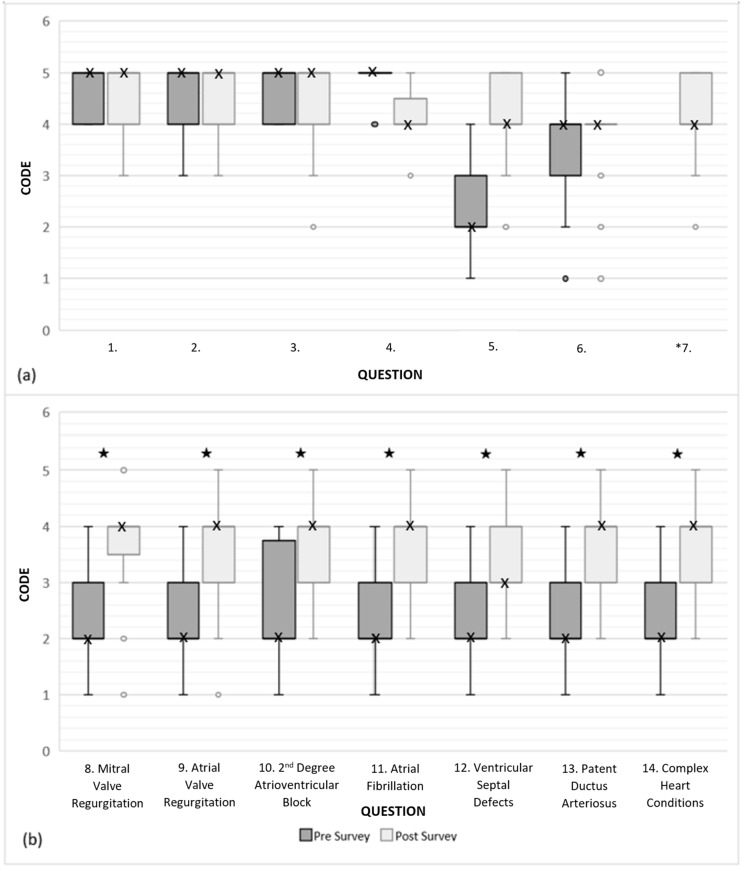
Comparison of the Likert values of Box and whisker plots comparing pre-intervention and post-intervention survey results for questions 8 to 14 (median and IQR). Sub-figure (**a**) shows comparison of responses to questions 1–7 and sub-figure (**b**) shows comparison of responses 8–14 of both surveys. The Y-axis score shows the student’s perceived ability to recognize the auscultatory findings associated with specific cardiac pathology using a 1–5 scale. For abbreviations, refer to Figure 3. ★ *p* < 0.0001; o Outliers; X Median. * There was no equivalent question for Q7 of the post-intervention survey in the pre-intervention survey.

Utilizing the learning resource was not mandatory, and additional surveys were optional even if students used the resource. Students were time-poor; therefore, completion rates of pre- and post-intervention surveys demonstrated positive student engagement with this training resource (Table 1). When completing the survey, there were specific questions asked that were compulsory to answer, for example, “What is the name of your first pet?” This enabled results from the individual students who responded in consecutive years to remain anonymous, yet be identified and excluded from further analysis, ensuring the data represented equaled the number of individuals surveyed. Two students completed the surveys in both their fourth and fifth years and their responses from their fifth year were excluded. Pooled responses for the pre-intervention surveys showed that the majority of respondents strongly agreed to being interested in more audio-visual learning resources for cardiac auscultation, and they thought these resources would benefit their practical studies and they would feel more competent in practicals if they had access to such a learning resource beforehand (Q1, Q2 and Q4, Figure 3a). For questions 1 to 4, the median score was five, which further indicated strong agreement with these statements (Figure 4a). For question 5, 47% of participants disagreed that they were already competent at auscultating sounds over each of the heart valves (Figure 3a). This was supported by a median score of two (Figure 4a). The majority of respondents agreed that they were familiar with identifying normal heart sounds (Q6, Figure 3a). Finally, respondents had a median score of two (Figure 4b) and disagreed that they were able to identify murmurs and arrythmias (Q8–14, Figure 3a).

Pooled responses for the pre-intervention surveys identified that the majority of respondents strongly agreed that they were interested in more audio-visual learning resources for cardiac auscultation, and that they thought these resources would benefit their practical studies and would feel more competent in practicals if they had access to such learning resources beforehand (Q1, Q2 and Q4.). Almost half of the participants did not feel competent at auscultating sounds over each of the heart valves (Q5). The majority of respondents agreed that they felt familiar with identifying normal heart sounds (Q6), but felt unable to identify murmurs and arrhythmias (Q8–14). Likert scale percentages and median values for each question are presented in Figure 3a and Figure 4a, respectively.

Pooled responses for the post-intervention survey showed that most participants strongly agreed that the resource helped them prepare for practicals, that the resource was easy to work with and that they prefer high-quality audio without background noise and artifacts for learning auscultation techniques (Q1–3, Figure 3b). The median score for each of these questions was five, supporting strong agreeance (Figure 4a). Additionally, students agreed that they felt more competent with their practical and diagnostic skills, that they could differentiate between normal and abnormal equine heart sounds, that they believed they could diagnose heart sounds with more accuracy and that they were familiar with identifying normal heart sounds after utilizing the resource (Q4–7, Figure 3b). Respondents also agreed that they believed that they could now identify murmurs and arrhythmias (Q8–14, Figure 3b). This was supported by a median of four for questions 8 to 11, 13 and 14 (Figure 4). 

**Figure 5:**
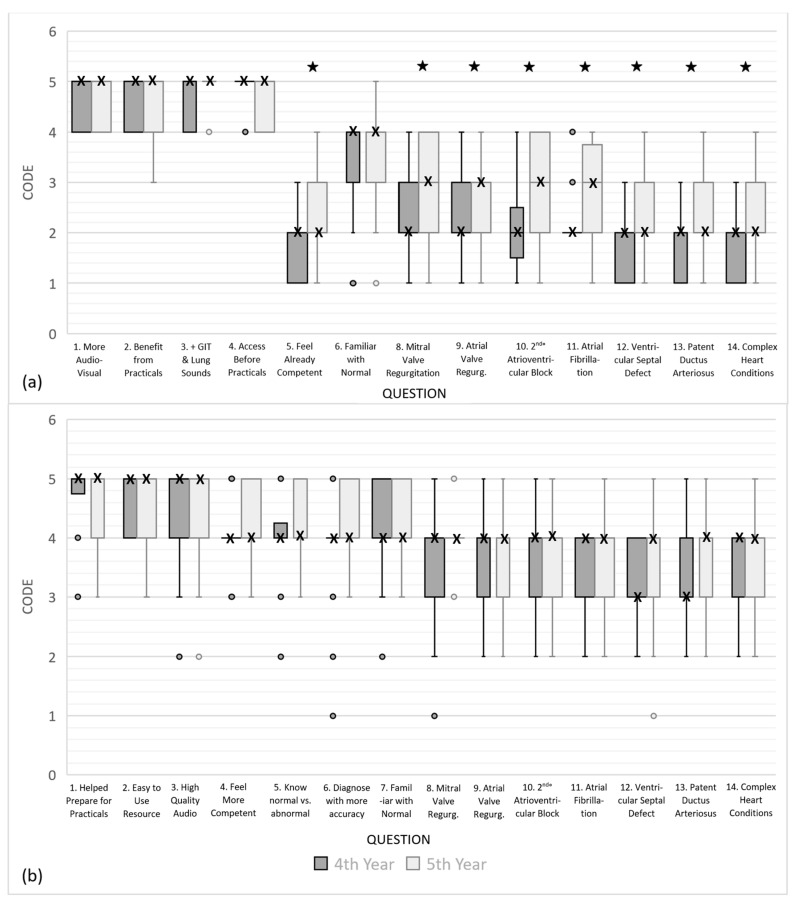
(**a**) Box and whisker plots comparing fourth- and fifth-year student’s pre-intervention Likert survey responses (median and IQR) (**b**) Box and whisker plots comparing fourth- and fifth-year students’ post-intervention Likert survey responses (median and IQR). The *y*-axis score shows student’s survey responses using a 1–5 scale for questions 1–14. For abbreviations, refer to Figure 3. ★ *p* < 0.05; o Outliers; X Median.

Data from all questions (Table 2) from the pooled data sets and year level responses were not normally distributed. Graphs showing responses for questions 8 to 14 (median and interquartile ranges (IQR)) for the fourth- and fifth-year cohorts are shown (Figure 5). Comparative analysis (*p* < 0.05) of the pre-intervention survey data identified that fourth-year students believed they were less competent at auscultating sounds over each of the heart valves and less able to identify arrhythmias and murmurs when compared to fifth-year students (Table 2 and Figure 5). No differences were detected in the post-survey questions between the two year levels (*p* > 0.05). Comparing the pooled data for pre- and post-survey questions 8 to 14, an improvement in students’ abilities to identify equine cardiac murmurs and arrhythmias after access to the learning resource was detected (*p* < 0.0001).

### 3.2. Qualitative Data 

A summary of the coded responses to the open-ended survey questions is shown in Table 3 and Supplementary File S3. The codes represent patterns in the data. For example, a participant’s response to the pre-intervention survey question: ‘What do you find most difficult about developing auscultation skills?’ was “I struggle to find the proper landmarks or perhaps do not have the proper technique for my auscultation to be of diagnostic quality”. This was coded under ‘understanding anatomy, physiology and placement of stethoscope’ (A&P) and ‘lack of opportunity to develop practical skills’ (PRAC). As a further example, the response “unless I can hear something, and be told/figure out—what I’m hearing is ‘this’, then it’s tricky to develop an ear for it” was coded under ‘having supervision when auscultating, teachers or learning resources’ (TEACH).

In relation to what students find most difficult about developing auscultation skills, most participants responded that having few opportunities to practice equine cardiac auscultation in the program was problematic. This perception was exemplified in responses such as “practical training in this area is extremely lacking in this program” and “lack of hands-on experience, we learn so much theory, but I had probably only heard 8 murmurs before 5th year”. Finding it hard to distinguish between normal and abnormal heart sounds, especially low-grade heart murmurs, was also a concern for many respondents. For example, a coded response for this was “hearing low grade heart murmurs” and “developing confidence to confidently identify mildly abnormal changes”.

**Figure 6:**
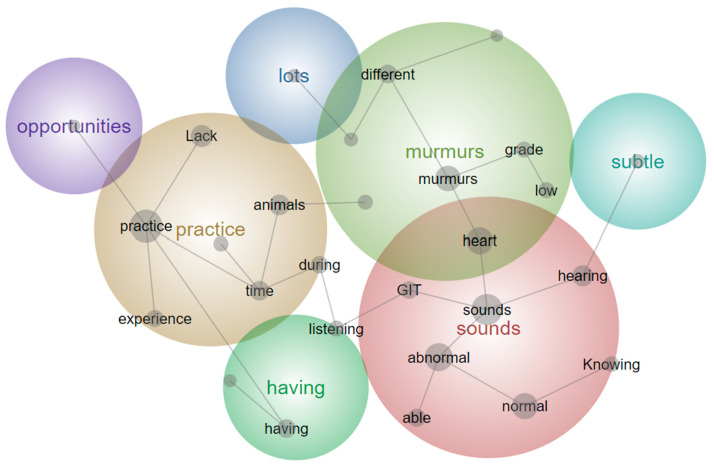
Leximancer concept map for ‘What do you find most difficult about developing auscultation skills?’ The large, colored circles are the overarching themes, whilst the words within are the concepts linking the themes.

Leximancer analysis of this question grouped responses similarly (Figure 6). The three dominant themes from the Leximancer analysis were “practice”, “sounds” and “murmurs”. The analysis found that students perceive it as difficult to distinguish between normal and abnormal, especially with subtle differences. Additionally, students reported it is difficult to diagnose low-grade murmurs and there is therefore a need for exposure to many different murmurs before they could feel competent with equine cardiac auscultation. 

In relation to the second open-ended question ‘What was the most helpful about the learning resource?’, thematic analysis showed that the audio-visual nature of the learning resource was most helpful to respondents. Some examples of responses included “the audios were very helpful, and the animations were very good to follow along with” and it was helpful to have “realistic sounds with gut sounds in background—useful for in practice to know what these sound like”. Many students also appreciated the access to a complete resource that was easy to use and had clear explanations and clear recordings embedded. Examples of correlated responses included “excellently presented and easy to use—very straightforward” and it was also helpful “having audio files and all the information in one place, it was very clearly structured and written”.

**Figure 7:**
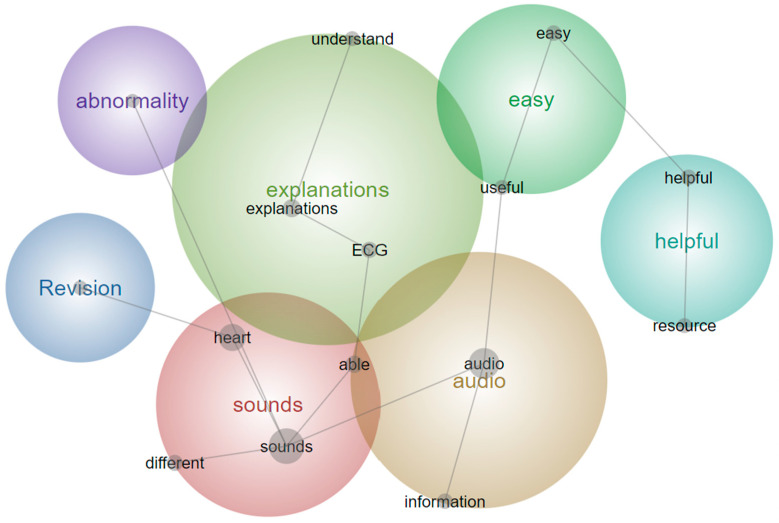
Leximancer concept map for ‘What was the most helpful about the learning resource?’ The large, colored circles are the overarching themes, whilst the words within are the concepts linking the themes.

The Leximancer Version 4 software analysis of this question was again very similar to the manual thematic analysis (Figure 7). Students found the explanations of abnormalities, ECG’s and heart sounds to be clear, helpful and easy to understand. From the visual representation of the program analysis (Figure 7), it was also clear that the audio-visual resource was easy to use and could also be utilized for revision purposes.

The thematic analysis of responses to the final question ‘What would improve this learning resource?’ centered around further content development: inclusion of more recordings; clearer audio and the inclusion of more technology and graphics on the slides of the resource. Examples of correlating responses include “slightly clearer sound examples”, “different recordings of the same problem as every case is different and hearing the same problem on different horses would sound different”, and digitally mapping the “corresponding position on ECG as the audio is playing”.

**Figure 8:**
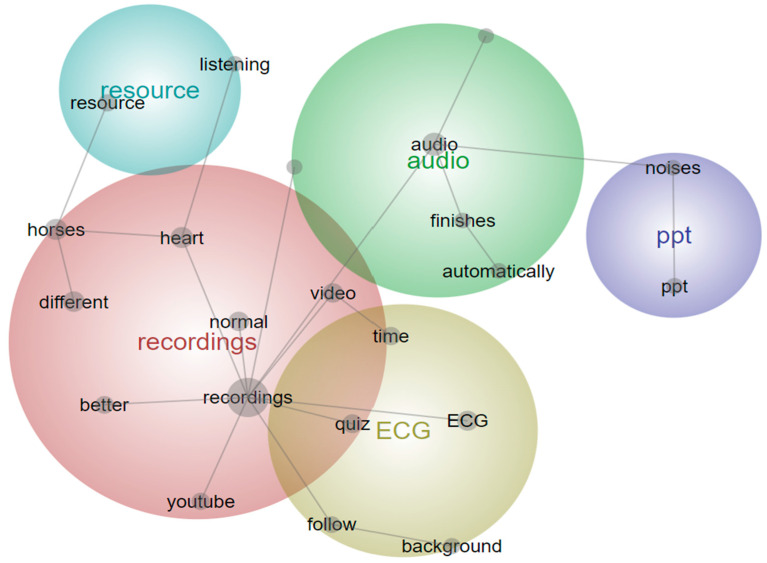
Leximancer concept map for ‘What would improve this learning resource?’ The large, colored circles are the overarching themes, whilst the words within are the concepts linking the themes; ppt = PowerPoint.

Leximancer software analysis identified similar themes for this question (Figure 8). The key themes the program found were recordings, audio and ECG. The inclusion of additional and higher quality audio recordings was the main suggestion for improvement of the learning resource. Additionally, different horse recordings, the inclusion of background ECG traces and embedding the YouTube recordings were suggested improvements.

## 4. Discussion

Analysis of pre- and post-intervention survey data showed that before accessing the resource, most respondents had a low perception of their ability to identify cardiac murmurs (mitral valve regurgitation, aortic valve regurgitation, tricuspid valve regurgitation, VSDs, PDAs and murmurs from complex congenital abnormalities) or common arrhythmias (second degree AVBs and atrial fibrillation). Their perceptions of competency for distinguishing between normal and abnormal cardiology findings, equine cardiology diagnostics and auscultation of murmurs and arrythmias improved after utilizing the learning resource. Respondents also appreciated the usability of the resource, the compilation of all information in one location, and believed that the use of the audio-visual resource assisted with their understanding of the auscultation of cardiac abnormalities. Additionally, thematic analysis and Leximancer software analysis revealed that students would like more practical opportunities to practice their auscultation and to be able to better differentiate between normal and abnormal heart sounds. Adequate cardiac auscultation is also a day-one clinical competency that medical students must meet [28]. “Simulation-based medical education” can improve students’ cardiac auscultation knowledge and practical skills [29]. Similar results were found when third-year medical students were examined after access to an online cardiac auscultation tutorial and simulation [28]. Access to online multimodal learning databases has been demonstrated to improve medical students’ abilities to distinguish murmurs [30,31,32].

Although the learning resource was highly recommended to all fourth- and fifth-year students, as we believed it would improve their knowledge, prepare them for the fourth-year equine auscultation practical class and clinical cases and likely improve their grades, it was not compulsory or directly assessed. Use of the learning resource and completion of the surveys was voluntary, and our human ethics required that the resource be made available to all students irrespective of whether they volunteered their time to complete the surveys. As the use of the resource and completion of the surveys had to be anonymous, we could not test if its utilization improved the student’s grades. It was our impression that most of the students did ultimately utilize the resource (often after the practical class, once they heard from their colleagues how useful it was). Unfortunately, as with most surveys, the number of students who gave up their time to complete the surveys was much lower than the number that utilized the resource. 

A higher percentage of students filled out the pre-intervention survey compared to the post-intervention survey, and fourth-year students had the highest response rate for both surveys. The COVID-19 pandemic impacted many teaching modalities, especially practical classes, across different professions [33,34,35,36,37], including the graduating class of 2022’s cardiac auscultation practicals (2021 fourth-year cohort). There has been a general shift to incorporating more online learning opportunities for veterinary students in recent years [37]. Veterinary students have also had more access to portable devices in COVID times, and due to social distancing restrictions, there has been an inability to conduct face-to-face lectures or even access hard-copy textbooks [37]. Potentially, fourth-year student response rates were higher due to willingness to view and complete surveys for the audio-visual resource during SDL, whereas fifth-year students were on clinical rotations and had less SDL time available. This cohort had their third-year physical examination practical canceled due to COVID-19 restrictions for face-to-face learning and were only exposed to one (40 min) fourth-year equine cardiac auscultation practical before beginning their fifth-year rotations in 2022. The two highest response rates were from the cohorts that undertook their fourth-year training, where the bulk of the auscultation was taught, under stricter COVID protocols and reduced practical training opportunities. It was theorized that because the students felt like their learning had been compromised, they were more willing to engage in additional SDL opportunities in preparation for their fifth-year clinical placements. 

Quantitative analysis showed that before undertaking the learning resource, fourth-year students felt less competent at auscultating over each of the heart valves and were less able to identify murmurs and arrhythmias (questions 5, and 8 through to 14) compared to fifth-year students. This is to be expected, as fifth-year students should be further advanced in their veterinary degree. However, after completion of the learning resource, no difference was detected between the fourth- and fifth-year responses for questions 5 and 8 to 14 on the post-intervention survey. The use of the learning resource appeared to equalize their perceived knowledge. There was a significant improvement in perceived competencies for both fourth- and fifth-year students when comparing the pre- and post-intervention survey responses (*p* value < 0.0001 for Questions 6–8), suggesting that the audio-visual resource helped with students’ understanding of equine cardiology and perceived competency of auscultation. Finally, there was also a strong interest in more resources for auscultation, as 100% of respondents either strongly agreed (65%) or agreed (35%) that audio-visual resources would benefit practical studies and students would be more competent if they had accessed the learning resource before practical classes. 

Thematic analysis can be criticized as being highly subjective and lacking in scientific rigor when measured against quantitative evaluative criteria [20,21,26,38]. However, qualitative research strategies, such as thematic analysis, allow researchers to generate a granularity of findings that cannot be obtained through quantitative means [20,21,26]. Participants can give voice to their individual perspectives and are not constrained by the researcher’s hypotheses [20,21,26]. Qualitative researchers can then take the full breadth of these individual perceptions and analyze them to describe and report patterns within data sets [20,21,26]. If the thematic analysis codes are well defined and representative, then a high intercoder agreement will be achieved [39]. In this study, the qualitative findings provided valuable insights into what students perceived to be particularly challenging about developing equine auscultation skills and how they perceived the learning resource could be improved. 

Through both comparative analyses of the open-ended survey questions utilizing a thematic analysis technique and the Leximancer software program, it was evident that students desired more practical opportunities to improve their auscultation skills throughout their studies. Respondents also relayed that the learning resource helped distinguish normal from abnormal sounds, and also identified common internal and external background noises. Some students believed that this made the identification of abnormalities more difficult, but most appreciated that these noises were reflective of real-world clinical practice. The utilization of both methods of qualitative analysis offered quality control of results and reduced the likelihood of individual bias [24,38].

Qualitative analysis determined that students found it difficult to diagnose low-grade murmurs and needed exposure to lots of different murmurs before they could feel competent with equine cardiac auscultation. New graduates are not expected to be competent at differentiating all murmurs by auscultation [40]. Expected day one competencies include identifying between normal and abnormal, the location of the point of maximum intensity of the murmur, the timing of the murmur (systolic/diastolic), grade of the murmur and the formulation of a differential list [40]. To understand the sound, students need to understand the anatomy and physiology/pathophysiology. Characteristic sounds allow the detection of an abnormality for further investigation by electrocardiogram or echocardiology to determine the appropriate prognosis and necessity of treatment or safety recommendations. Day one competencies can be improved after many years of practice, continuing education, ongoing case-based reading, internships, residencies, post-graduate competency courses, exams and board certification [31,41]. 

One of the limitations of this study was the moderate completion rate of both surveys. The overall community survey completion rates of the cohorts were approximately 20% for the pre-intervention survey and 13% for the post-intervention survey. The voluntary completion rate of student evaluations of fourth- and fifth-year veterinary students at UQ is between 10% and 25%; therefore, the completion rates for this study were comparable. To enhance program learning outcomes, particularly in the light of COVID restrictions on practical teaching, all students were provided equal access to the learning resource irrespective of whether they elected to complete one or both surveys. Therefore, it was not possible to have a control group that did not have access to the learning resource and test the difference in knowledge between students who did or did not utilize the resource. The surveys were completed voluntarily with no incentive or benefit from survey completion. The low response rate to the post-intervention survey is acknowledged; however, completion was dependent on the student’s generosity to donate their time to assist with this study. Although we did not detect a difference in perceived competencies between fourth- and fifth-year students after utilizing the resource, this may have been a true lack of difference (with the resource equalizing their knowledge), or failure to detect a difference due to inadequate power. It should also be noted that whilst the resource indicates where to auscultate heart sounds on a horse, it does not replace practically finding the heart sounds on a live horse, it just better prepares students before practicals. 

During practical classes and clinical rotations, it was noted by lecturers and tutors that individual students frequently commented on how useful the learning resource was and encouraged their colleagues to utilize the resource. The resource was likely utilized more frequently than the completion of the actual surveys. As students’ learning is cumulative across the curriculum with ongoing overlapping multispecies courses, it was not possible to determine the exact improvement in students’ perceived abilities attributable solely to this learning resource. Clinical equine rotation occurs throughout their final year, and students likely had greater knowledge at the end of their clinical year when compared to the start of the clinical year. Anecdotally, most students completed the post-survey questions immediately after utilizing the resource, but some students did complete it within the two-week equine medicine rotation and their knowledge may have improved during the rotation. Highly motivated students were more likely to make time to participate in voluntary additional learning opportunities, and the results of the surveys may not be representative of the entire cohort. Students with an interest in equine medicine or previous horse experience may have had improved perceptions of their abilities before utilizing the resource. A question in the pre-survey regarding the level of previous experience with horses to determine if there was any difference in the pre- and post-survey results for students with prior equine experience would be a valuable addition to future work.

This study determined that the students’ perception of knowledge improved after the utilization of the targeted, audio-visual training resource. However, actual diagnostic capabilities before or after utilizing the learning resource, or between students who did or did not utilize the resource, were not determined because no form of physical assessment was able to be implemented during COVID restrictions and managing their ongoing impacts. It is well illustrated by Cook et al. (2012) that one must be careful about the claims made about whether an educational intervention worked or not [42]. However, as the perception of the resource was very positive, then it appears worthwhile to embed the resource within the curriculum, possibly incorporating more quiz questions within the resource and continuing to encourage students to use the resource before practical classes and as a means to prepare for examinations in earlier years. High-quality, audio-based quiz questions before and after using the resource, or in end-of-semester equine cardiology exams, could be used to assess the effectiveness of the resource; however, as a clinical skill, this should ideally be measured via a structured skills exam. 

Traditionally, veterinary students learn about cardiology based on a foundation in physiology and pathophysiology. Auscultation skills are practiced on a variety of species, comparatively providing cumulative knowledge. Although this learning resource was centered around equine cardiology, the basic skills learned would benefit all students irrespective of their interest in equine practice. As horses have large hearts and slow heart rates, in addition to lack of panting (dogs) or purring (cats), horses with a placid temperament provide an ideal species for students to learn to auscultate normal heart sounds over different valves, in addition to common murmurs and arrhythmias. This resource was tested in students affected by COVID lockdowns and its benefits were proven within a framework of limited other opportunities that disrupted the usual cumulative teaching program. Student feedback indicated that this multimodal teaching resource should be deployed repeatedly to third-, fourth- and fifth-year students. Our resource is provided for download in an open-access platform to benefit veterinary students worldwide.

Electronic stethoscopes have the potential for artificial intelligence and algorithm-generated diagnoses [16]. As more recordings are taken of abnormal and normal sounds, the algorithms can be improved with greater accuracy [43]. The audio data collected during this project will be utilized to assist with engineering stethoscope algorithms tailored for use in horses to benefit clinical examinations. The development of such algorithms will assist the veterinary profession on a global scale and aid in creating algorithms for other species. This will ultimately result in increased telemedicine capabilities to assist rural, remote and even third-world veterinarians [13,44]. Adding recordings to a patient’s electronic medical record also helps to document future disease progression [13].

## 5. Conclusions

This research sought to improve the quality of the learning resources available to the veterinary science program at UQ, and through presentations and publications to assist the National and International veterinary education community. There is a plethora of teaching resources available, but few have quantitative data to support their efficacy as a teaching device. Results established that the targeted, audio-visual training resource helped veterinary students improve their understanding and perception of competency and confidence when auscultating equine cardiac sounds. This improved perception of auscultatory competency may assist in communicating clinical findings with clients in the future. Similar to all studies that rely on voluntary completion of surveys, there are limitations due to volunteer bias and a moderate participation rate. However, forcing survey participation is unethical, only providing the resource to a subset of students is not equitable and the workload required to implement pre- and post-intervention skills exams during clinical training for large cohorts is unmanageable. The post-intervention survey responses indicated that the audio-visual learning resource helped students prepare for practical classes and rotations and was easy to utilize. Respondents agreed that they could differentiate between normal and abnormal equine heart sounds and could recognize various murmurs and arrhythmias after accessing the resource. Irrespective of future testing, it was clear that students found this resource helpful to their learning, and sharing this resource would likely benefit the wider veterinary population. The use of this audio-visual resource shows the effectiveness of the implementation of such multimodal presentations for use in furthering veterinary education. The ultimate impact is increased confidence in the diagnostic capabilities of future veterinarians when auscultating cardiac abnormalities.

## Figures and Tables

**Table 1:** Audio-visual Auscultation Resource Survey Response Rates.

Year & Total Responses		Pre-Intervention Cohort		Post-Intervention Cohort	
Year & Cohort	Total	Responses	Percentage	Responses	Percentage
2021 4th year	112	35	31%	26	23%
2021 5th year	110	16	15%	9	8%
2022 4th year	119	14	12%	8	7%
2022 5th year	112	24	21%	14	13%

**Table 2:** Differences between fourth- and fifth-year responses for pre- and post-intervention surveys.

Pre-Intervention Survey Difference for 4th versus 5th Year Responses		Post-Intervention Survey Difference for 4th and 5th Year Responses		Pre and Post-Survey Comparison of Pooled Responses	
Question	*p* Value	Question	*p* Value	Question	*p* Value
1. I am interested in more audio-visual learning resources for cardiac auscultation	0.66	1. This resource helped me prepare for practicals/training	0.50	Questions 1–7 are not comparable between the pooled pre-and post-intervention responses	
2. I think these resources would benefit practical studies immensely	0.99	2. The resource was easy to work with	0.11		
3. I would like to see more resources with recordings of GIT and lung sounds also	0.14	3. I prefer clear high-quality audio without background noise and artefacts for learning new auscultation techniques,	0.87		
4. I/students would feel more competent in practicals if we/they had access to such learning resources beforehand	0.46	4. I feel more competent with my practical and diagnostic skills	0.28		
5. I am already competent at auscultating sounds over each of the heart valves	0.005 *	5. I can differentiate between normal and abnormal equine heart sounds	0.30		
6. I am familiar with identifying normal heart sounds	0.72	6. I believe I can diagnose heart sounds with more accuracy after using the resource	0.14		
7. #		7. I am familiar with identifying normal heart sounds	0.32		
8. I am able to identify mitral valve regurgitation (systolic murmur)	0.0001 *	8. I am able to identify mitral valve regurgitation (systolic murmur)	0.11	8.	<0.0001 √
9. I am able to identify aortic valve regurgitation (diastolic murmur)	0.007 *	9. I am able to identify aortic valve regurgitation (diastolic murmur)	0.36	9.	<0.0001 √
10. I am able to identify second degree atrioventricular blocks (AVB)	0.0001 *	10. I am able to identify second degree atrioventricular blocks (AVB)	0.24	10.	<0.0001 √
11. I am able to identify atrial fibrillation	0.0008 *	11. I am able to identify atrial fibrillation	0.55	11.	<0.0001 √
12. I am able to identify ventricular septal defects (VSD)	0.005 *	12. I am able to identify ventricular septal defects (VSD)	0.17	12.	<0.0001 √
13. I am able to identify a patent ductus arteriosus (PDA)	0.008 *	13. I am able to identify a patent ductus arteriosus (PDA)	0.05	13.	<0.0001 √
14. I understand how to diagnose complex heart conditions	0.01*	14. I understand how to diagnose complex heart conditions	0.29	14.	<0.0001 √

* *p* < 0.05 = difference between the fourth and fifth-year student responses; √ *p* < 0.0001 = difference between pre- and post-intervention pooled survey responses; # Notes: One less question was asked on the pre-intervention survey, and question numbers were re-calibrated for simplicity in results. The full original data set is provided in Supplementary Data.

**Table 3:** Definitions of coding nodes for efficacy of audio-visual resource survey questions.

**Pre-Intervention Survey Question: ‘What Do You Find Most Difficult about Developing** **Auscultation Skills?’**			
**Title**	**Code**	**Attributes**	**Responses**
Practical skills	PRAC	Few opportunities to gain practical skills in auscultation	41
Abnormal versus normal	A&N	Difficulty in distinguishing between normal and abnormal cardiac sounds/pathologies to make a diagnosis	23
Anatomy and physiology	A&P	Understanding anatomy, physiology and placement of stethoscope for auscultation	16
Heart murmur	MUR	Heart murmurs specifically subtle/low-grade heart murmurs	13
Teaching and learning	TEA	Having supervision when auscultating, teachers or learning resources	11
GIT auscultation	GIT	GIT auscultation	3
Other	OTH	Anything that doesn’t fit any of the above	4
**Post-intervention survey question: ‘What was most helpful about the learning resource?’**			
**Title**	**Code**	**Attributes**	**Responses**
Complete resource	RES	The helpfulness of having the material condensed into a single resource	5
Audio visual	AV	The audio or visuals being helpful or the inclusion of audio recordings and/or ECG’s in the resource	46
Abnormal	ABN	Including information regarding abnormalities or pathologies	6
Nothing	NOT	The resource not being helpful	1
Revision	REV	Anatomy and/or revision	5
Heart murmur	MUR	Heart murmurs specifically	5
Clear explanation	CLE	Clear explanations and descriptions	13
Ease of use	EASE	Ease of use	2
Understanding	UN	Understanding information presented in the resource	5
Other	OTH	Anything that doesn’t fit any of the above	1
**Post-intervention survey question: ‘What would improve the learning resource?’**			
**Title**	**Code**	**Attributes**	**Responses**
Clearer explanations	CLA	The explanations and clarity of information in the resource	3
Audio	AUD	The clearness of audio recordings or inclusion of more recordings	17
Concise	CON	Improving the conciseness of the slides	5
Nothing	NOT	The resource not needing any improvements	1
Cases	CAS	More cases and/or inclusion of more quiz questions	5
More interaction	INT	Technology and improved graphics to make it even more interactive	17
Accessibility	ACC	More accessibility to the resource and/or incorporation in more areas of the curriculum	2
Additional information	INFO	Additional information to include	6
Other	OTH	Anything that doesn’t fit any of the above	2

## Data Availability

The data presented in this study are available on request from the corresponding author. The data are not publicly available due to ethical restrictions.

## Supplementary Materials

The following supporting information can be downloaded at: https://www.mdpi.com/article/10.3390/ani14091341/s1. File S1. Learning Resource; File S2. Pre-intervention Survey; File S3. Post-intervention Survey; File S4. Thematic analysis coding and questions.
