# Supplementary material for: Assessment of a Teaching Module for Cardiac Auscultation of Horses by Veterinary Students
Source: Animals (Basel). 2024 Apr 29;14(9):1341. doi: 10.3390/ani14091341 (PMC11083587; doi:10.3390/ani14091341)
Supplement: Supplementary file 1 [file animals-14-01341-s001.zip › File S1. Final Student Version Cardiovascular Recordings.pptx]

## Slide 1
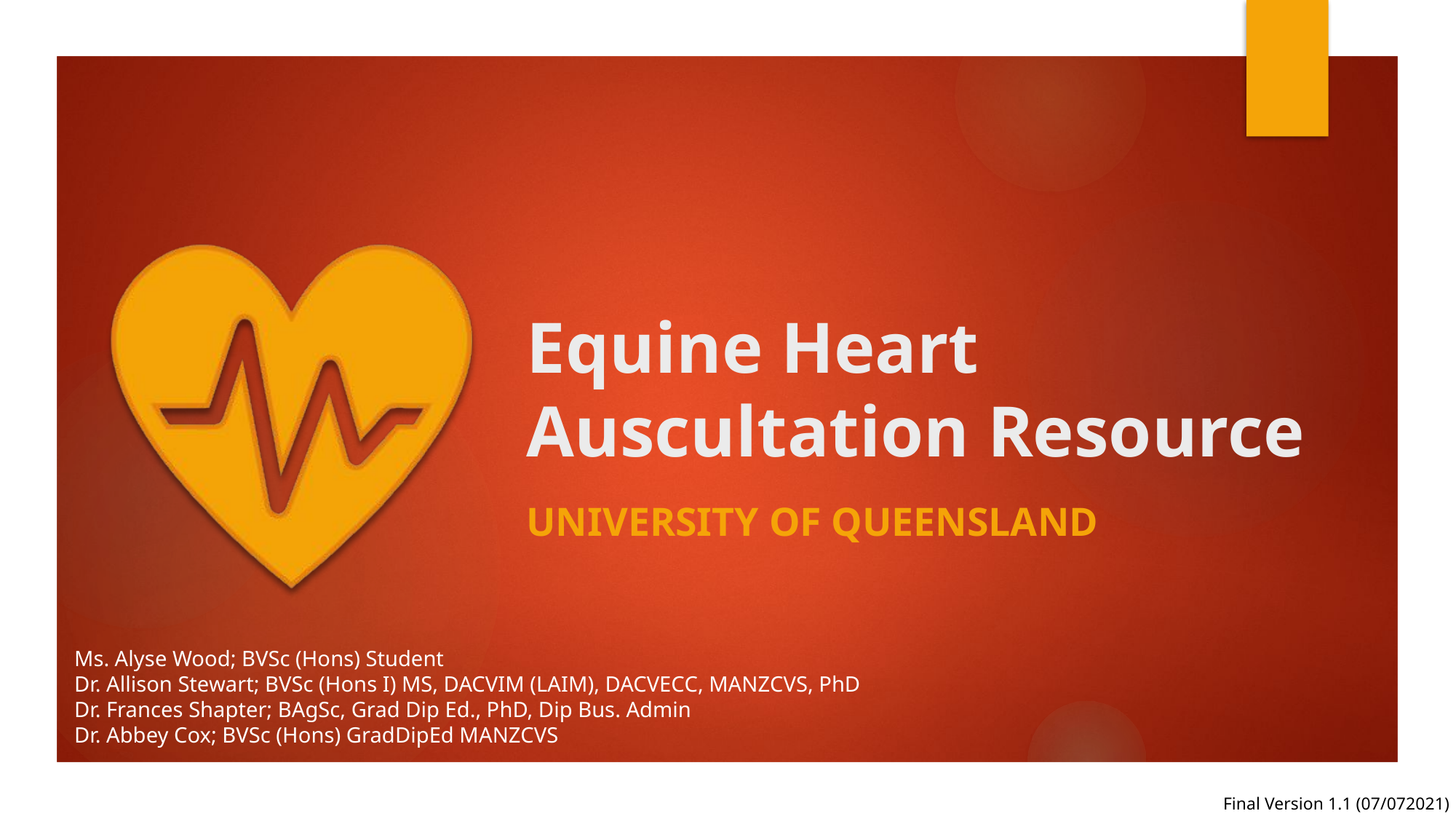

# Equine Heart Auscultation Resource
University of Queensland
Ms. Alyse Wood; BVSc (Hons) Student
Dr. Allison Stewart; BVSc (Hons I) MS, DACVIM (LAIM), DACVECC, MANZCVS, PhD
Dr. Frances Shapter; BAgSc, Grad Dip Ed., PhD, Dip Bus. Admin
Dr. Abbey Cox; BVSc (Hons) GradDipEd MANZCVS
Final Version 1.1 (07/072021)

## Slide 2
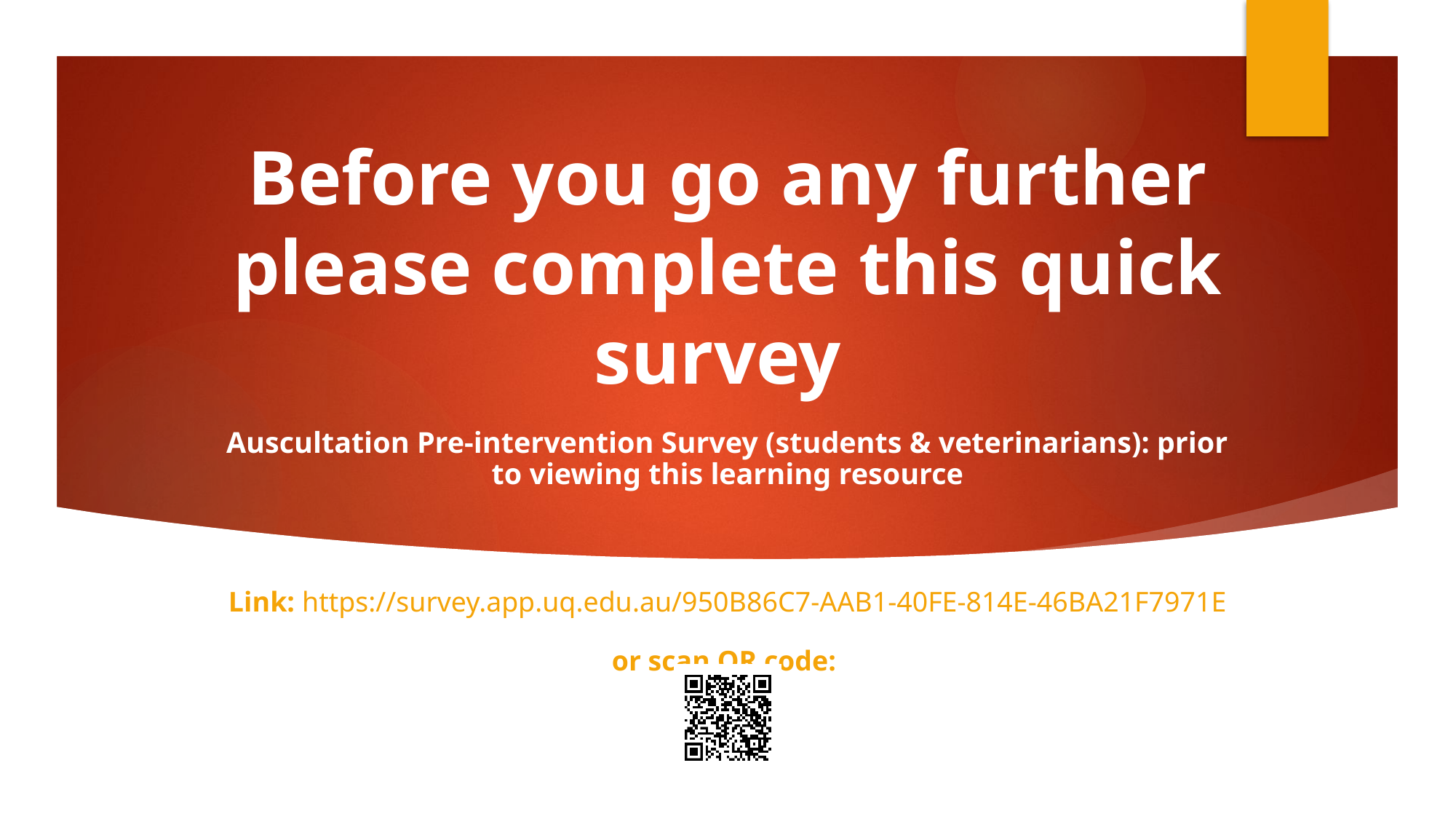

Before you go any further please complete this quick survey
Auscultation Pre-intervention Survey (students & veterinarians): prior to viewing this learning resource
Link: https://survey.app.uq.edu.au/950B86C7-AAB1-40FE-814E-46BA21F7971E
or scan QR code:

## Slide 3
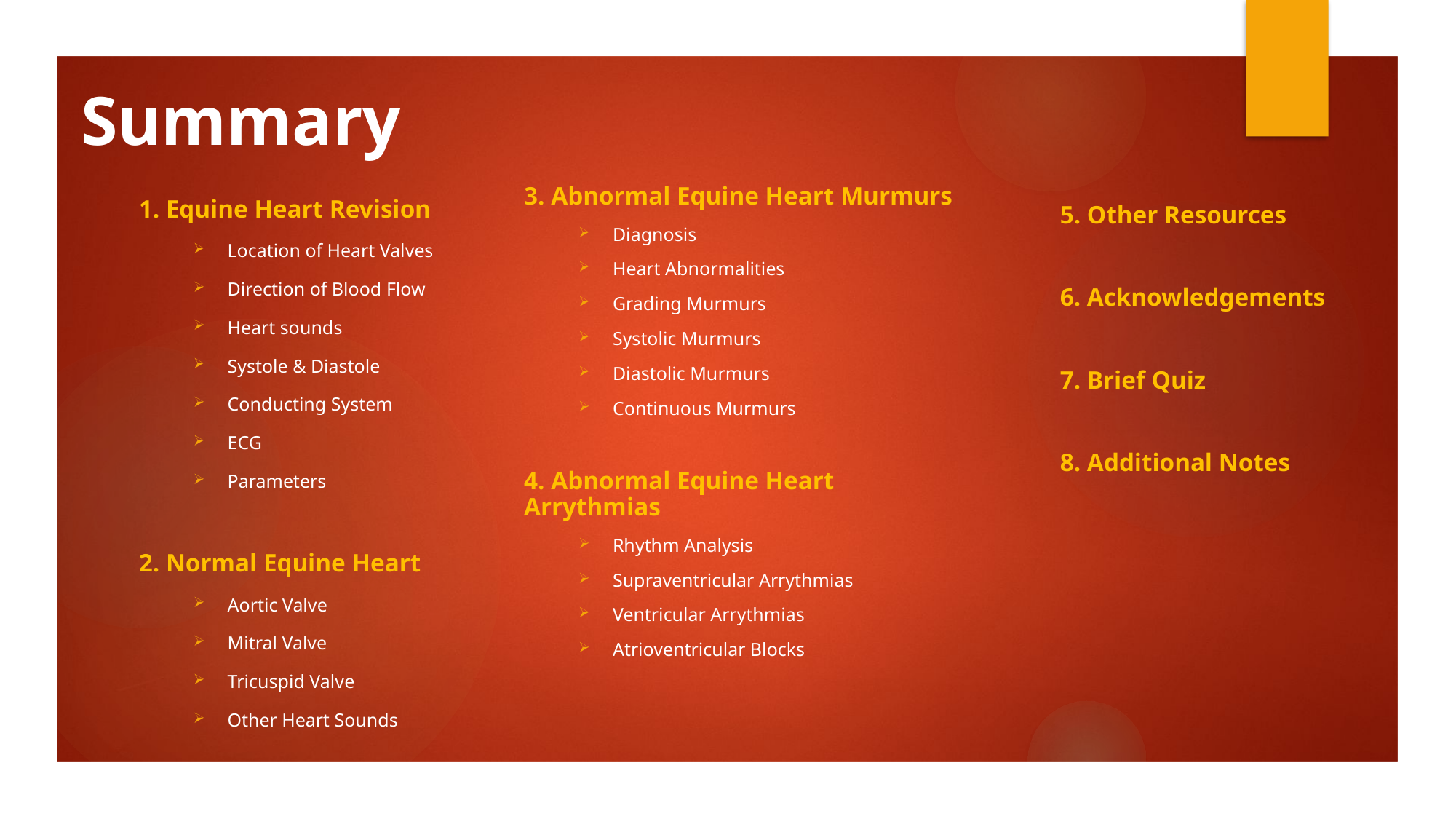

Summary
3. Abnormal Equine Heart Murmurs
Diagnosis
Heart Abnormalities
Grading Murmurs
Systolic Murmurs
Diastolic Murmurs
Continuous Murmurs
4. Abnormal Equine Heart Arrythmias
Rhythm Analysis
Supraventricular Arrythmias
Ventricular Arrythmias
Atrioventricular Blocks
1. Equine Heart Revision
Location of Heart Valves
Direction of Blood Flow
Heart sounds
Systole & Diastole
Conducting System
ECG
Parameters
2. Normal Equine Heart
Aortic Valve
Mitral Valve
Tricuspid Valve
Other Heart Sounds
5. Other Resources
6. Acknowledgements
7. Brief Quiz
8. Additional Notes

## Slide 4
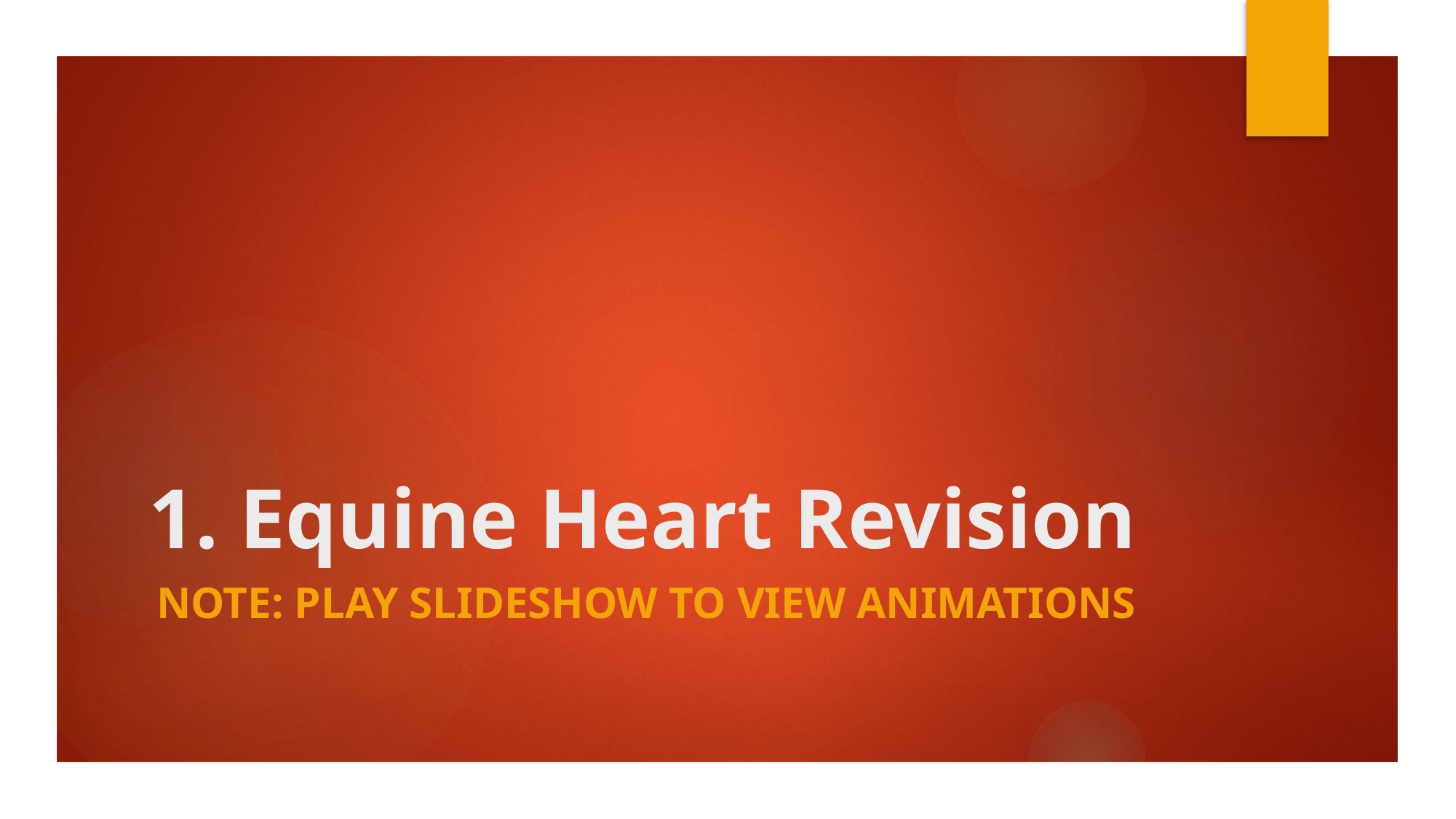

# 1. Equine Heart Revision
Note: Play Slideshow to view animations

## Slide 5
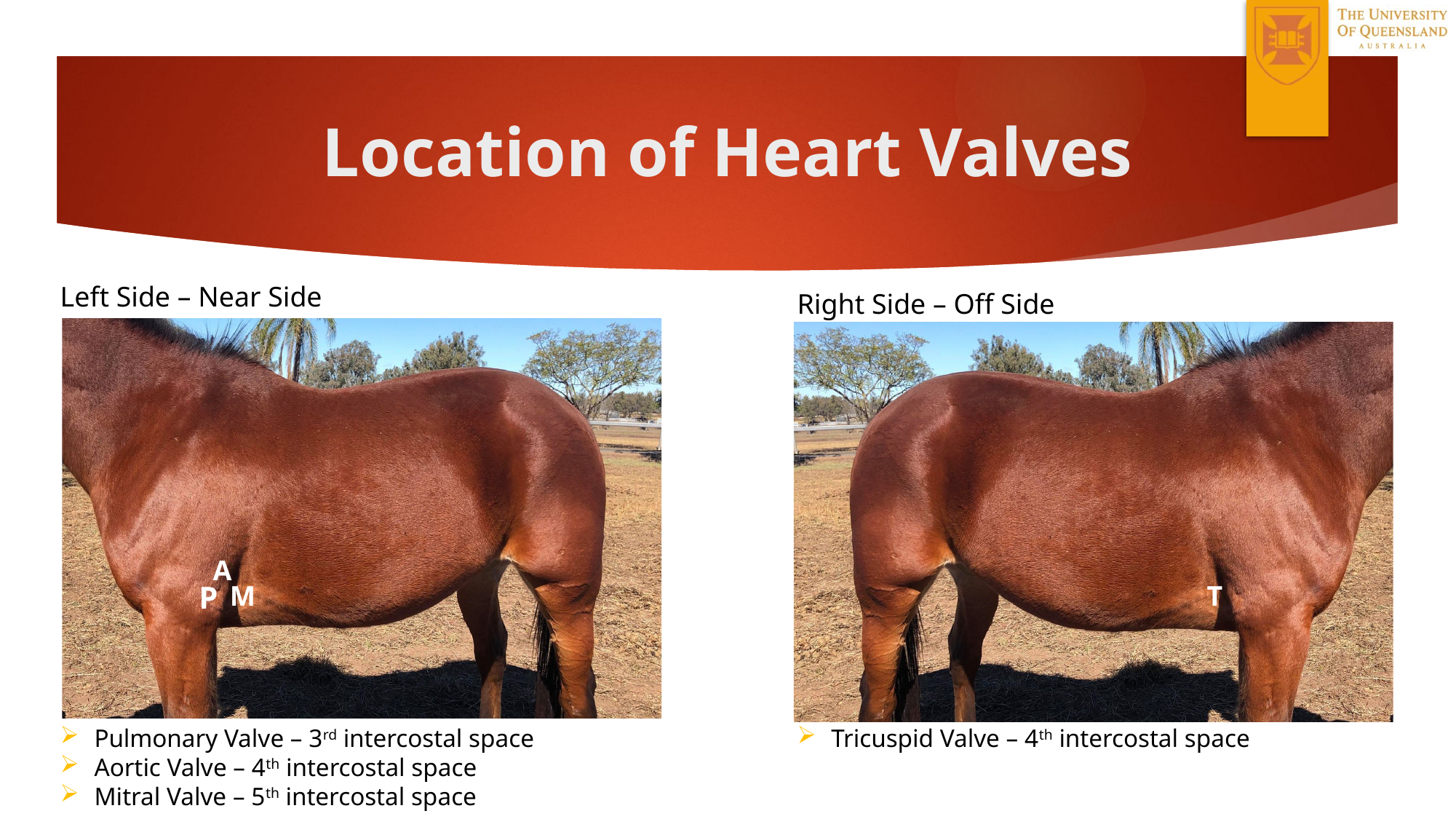

# Location of Heart Valves
Left Side – Near Side
Right Side – Off Side
A
P
M
T
Pulmonary Valve – 3rd intercostal space
Aortic Valve – 4th intercostal space
Mitral Valve – 5th intercostal space
Tricuspid Valve – 4th intercostal space

## Slide 6
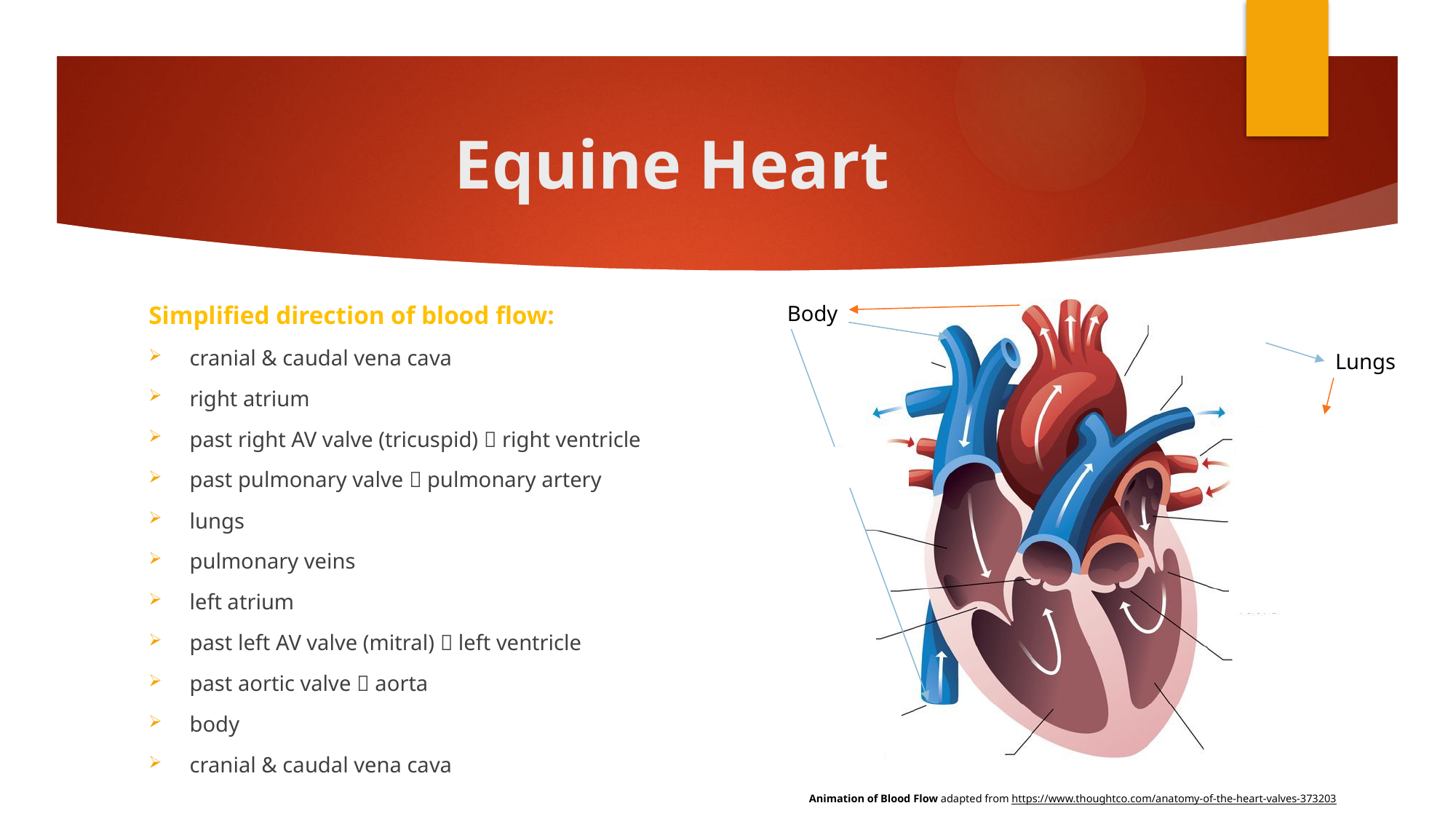

# Equine Heart
Simplified direction of blood flow:
cranial & caudal vena cava
right atrium
past right AV valve (tricuspid)  right ventricle
past pulmonary valve  pulmonary artery
lungs
pulmonary veins
left atrium
past left AV valve (mitral)  left ventricle
past aortic valve  aorta
body
cranial & caudal vena cava
Body
Cranial vena cava
Lungs
Caudal vena cava
Animation of Blood Flow adapted from https://www.thoughtco.com/anatomy-of-the-heart-valves-373203

## Slide 7
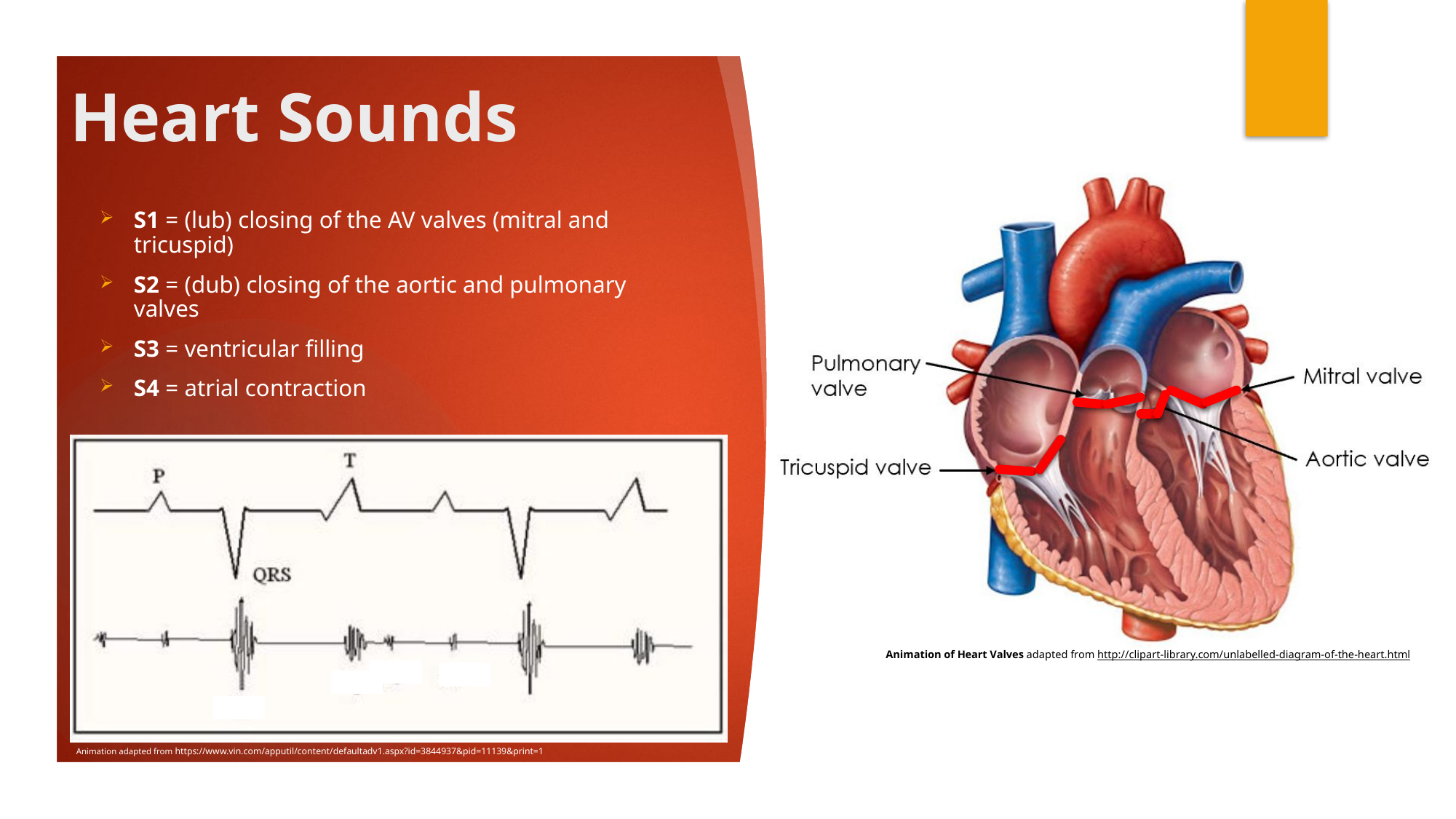

Heart Sounds
S1 = (lub) closing of the AV valves (mitral and tricuspid)
S2 = (dub) closing of the aortic and pulmonary valves
S3 = ventricular filling
S4 = atrial contraction
Animation of Heart Valves adapted from http://clipart-library.com/unlabelled-diagram-of-the-heart.html
Animation adapted from https://www.vin.com/apputil/content/defaultadv1.aspx?id=3844937&pid=11139&print=1

## Slide 8
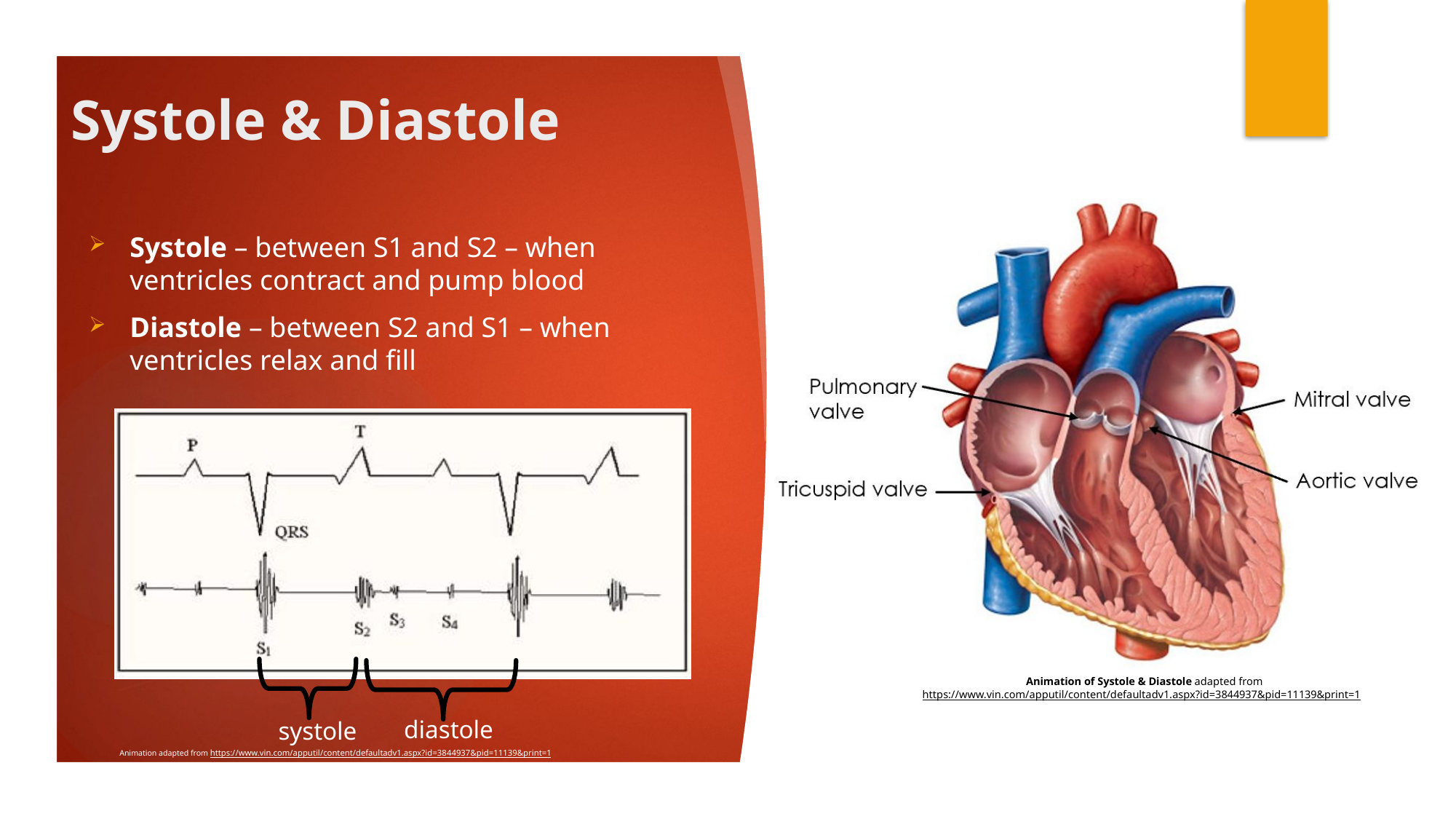

# Systole & Diastole
Systole – between S1 and S2 – when ventricles contract and pump blood
Diastole – between S2 and S1 – when ventricles relax and fill
Animation of Systole & Diastole adapted from https://www.vin.com/apputil/content/defaultadv1.aspx?id=3844937&pid=11139&print=1
diastole
systole
Animation adapted from https://www.vin.com/apputil/content/defaultadv1.aspx?id=3844937&pid=11139&print=1

## Slide 9
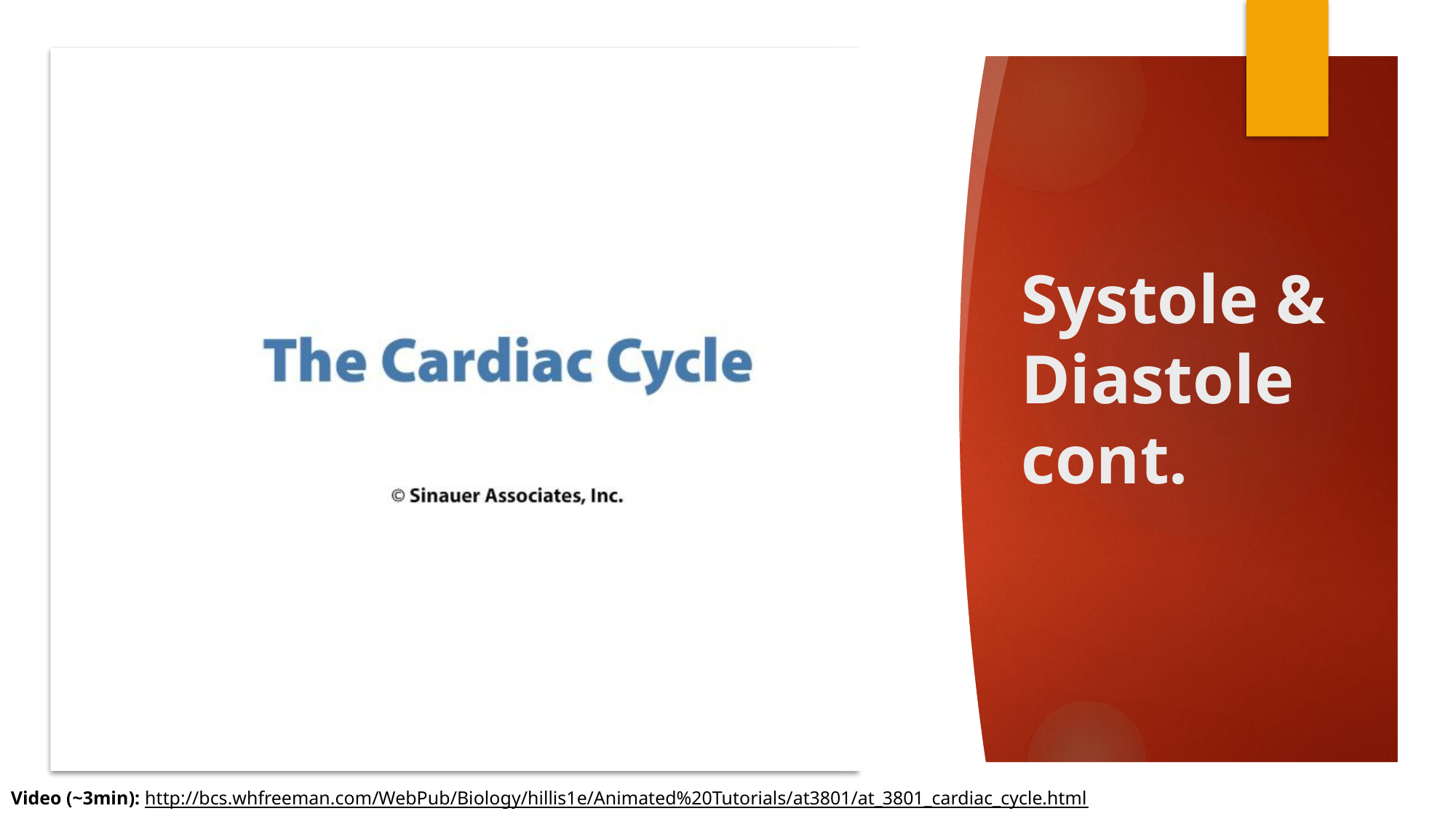

# Systole & Diastole cont.
Video (~3min): http://bcs.whfreeman.com/WebPub/Biology/hillis1e/Animated%20Tutorials/at3801/at_3801_cardiac_cycle.html

## Slide 10
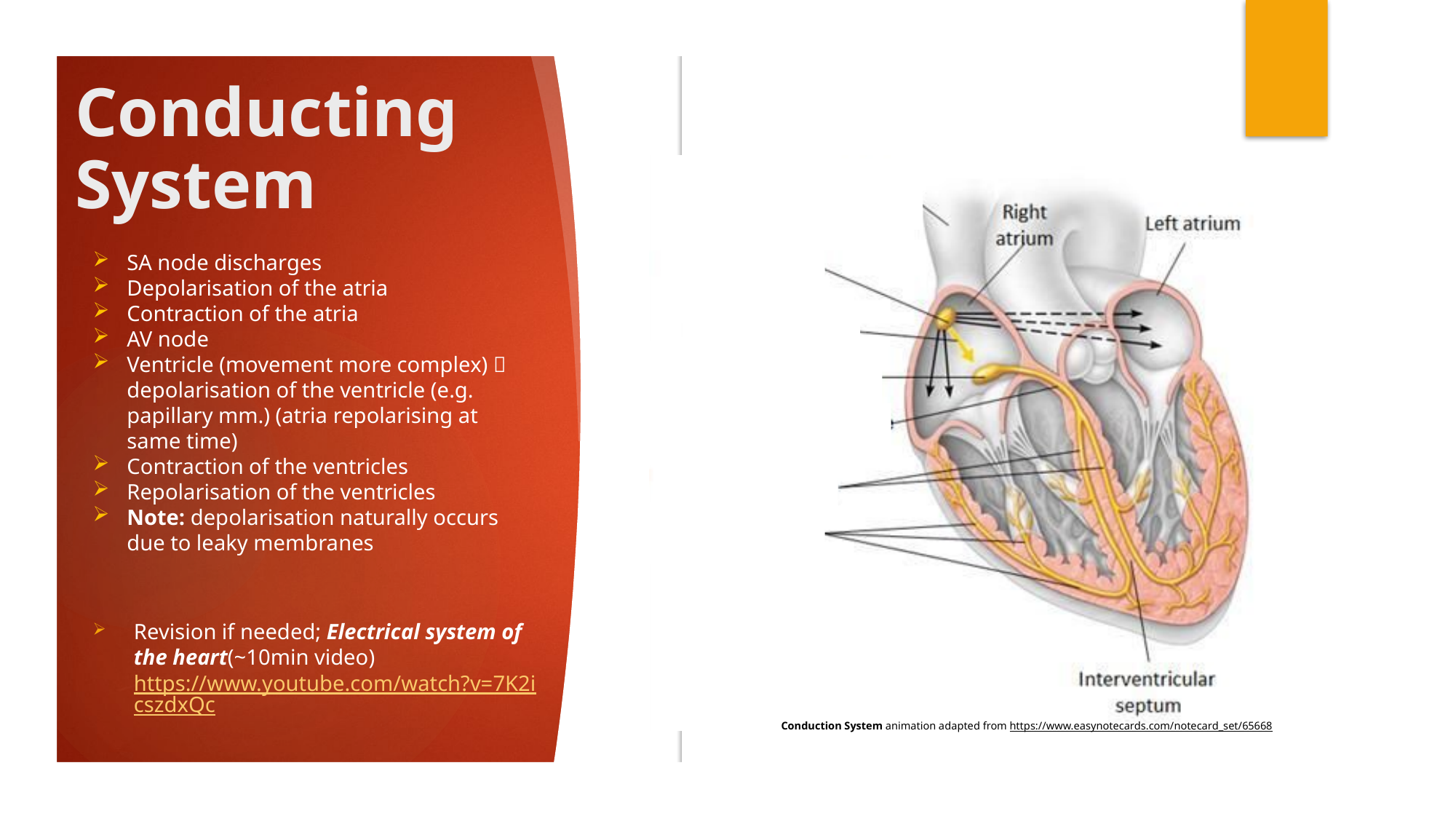

# Conducting System
SA node discharges
Depolarisation of the atria
Contraction of the atria
AV node
Ventricle (movement more complex)  depolarisation of the ventricle (e.g. papillary mm.) (atria repolarising at same time)
Contraction of the ventricles
Repolarisation of the ventricles
Note: depolarisation naturally occurs due to leaky membranes
Revision if needed; Electrical system of the heart(~10min video) https://www.youtube.com/watch?v=7K2icszdxQc
Conduction System animation adapted from https://www.easynotecards.com/notecard_set/65668

## Slide 11
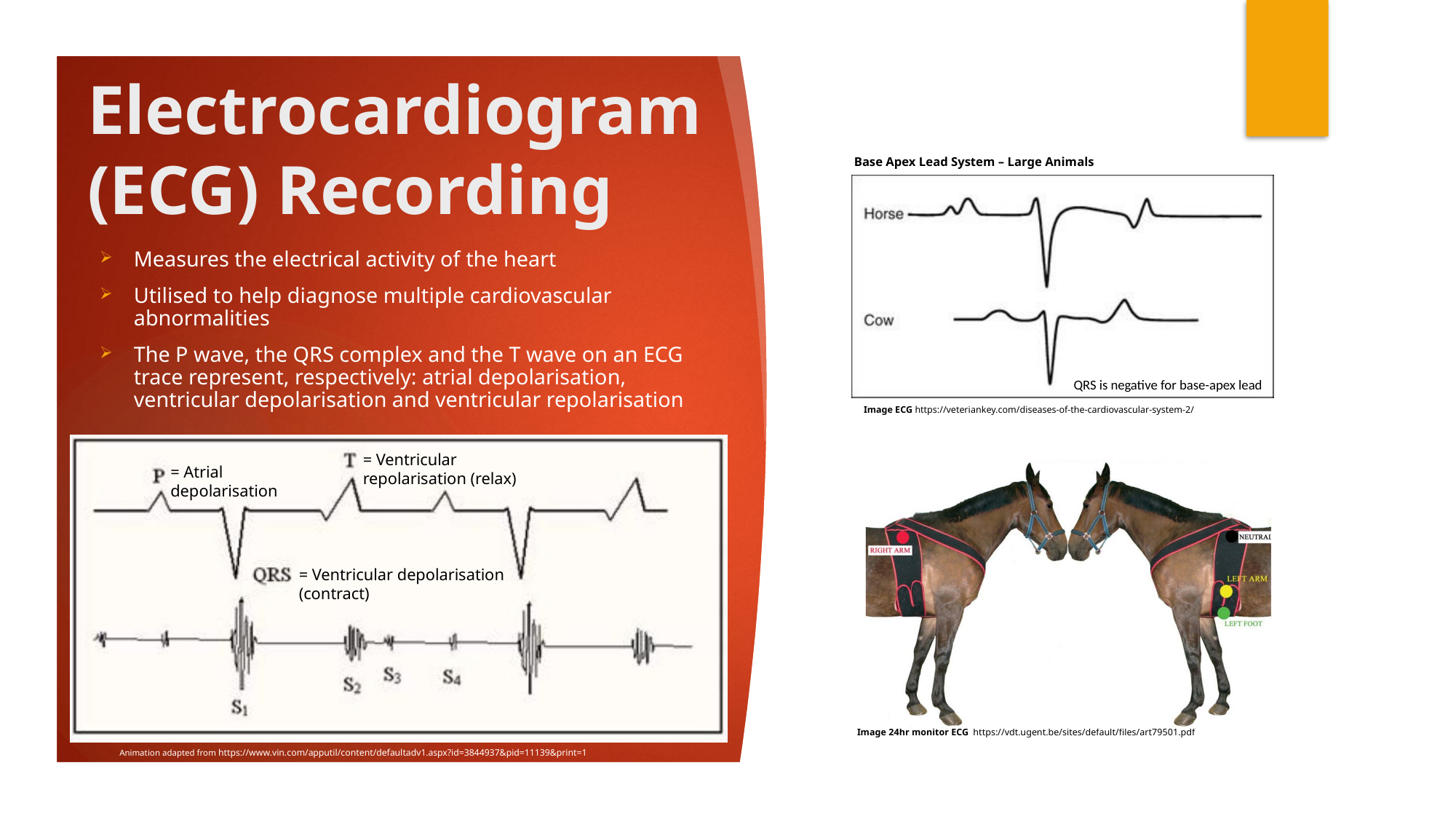

# Electrocardiogram (ECG) Recording
Base Apex Lead System – Large Animals
Measures the electrical activity of the heart
Utilised to help diagnose multiple cardiovascular abnormalities
The P wave, the QRS complex and the T wave on an ECG trace represent, respectively: atrial depolarisation, ventricular depolarisation and ventricular repolarisation
QRS is negative for base-apex lead
Image ECG https://veteriankey.com/diseases-of-the-cardiovascular-system-2/
= Ventricular repolarisation (relax)
= Atrial depolarisation
= Ventricular depolarisation (contract)
Image 24hr monitor ECG https://vdt.ugent.be/sites/default/files/art79501.pdf
Animation adapted from https://www.vin.com/apputil/content/defaultadv1.aspx?id=3844937&pid=11139&print=1

## Slide 12
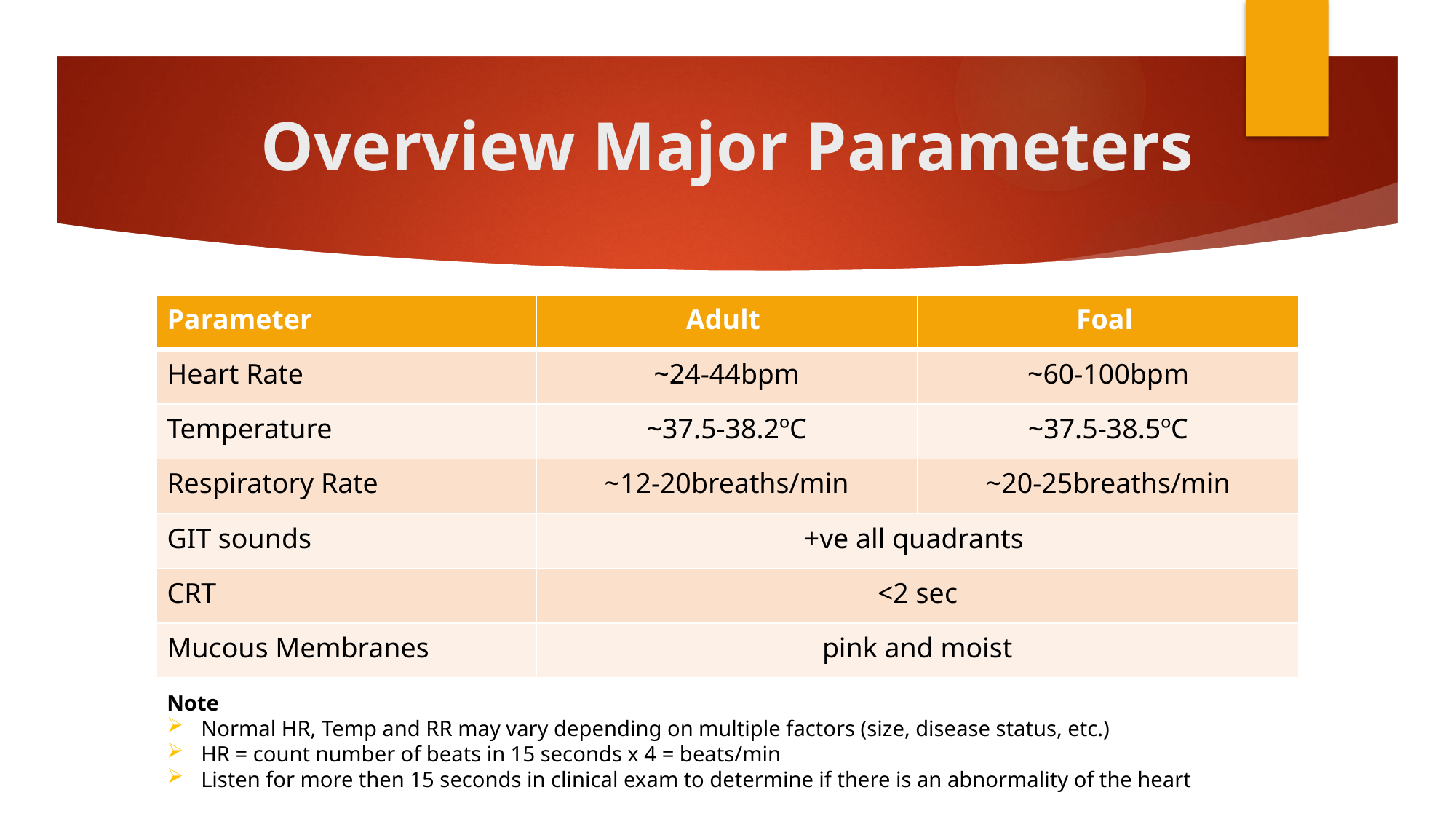

# Overview Major Parameters
| Parameter | Adult | Foal |
| --- | --- | --- |
| Heart Rate | ~24-44bpm | ~60-100bpm |
| Temperature | ~37.5-38.2ºC | ~37.5-38.5ºC |
| Respiratory Rate | ~12-20breaths/min | ~20-25breaths/min |
| GIT sounds | +ve all quadrants | |
| CRT | <2 sec | |
| Mucous Membranes | pink and moist | |
Note
Normal HR, Temp and RR may vary depending on multiple factors (size, disease status, etc.)
HR = count number of beats in 15 seconds x 4 = beats/min
Listen for more then 15 seconds in clinical exam to determine if there is an abnormality of the heart

## Slide 13
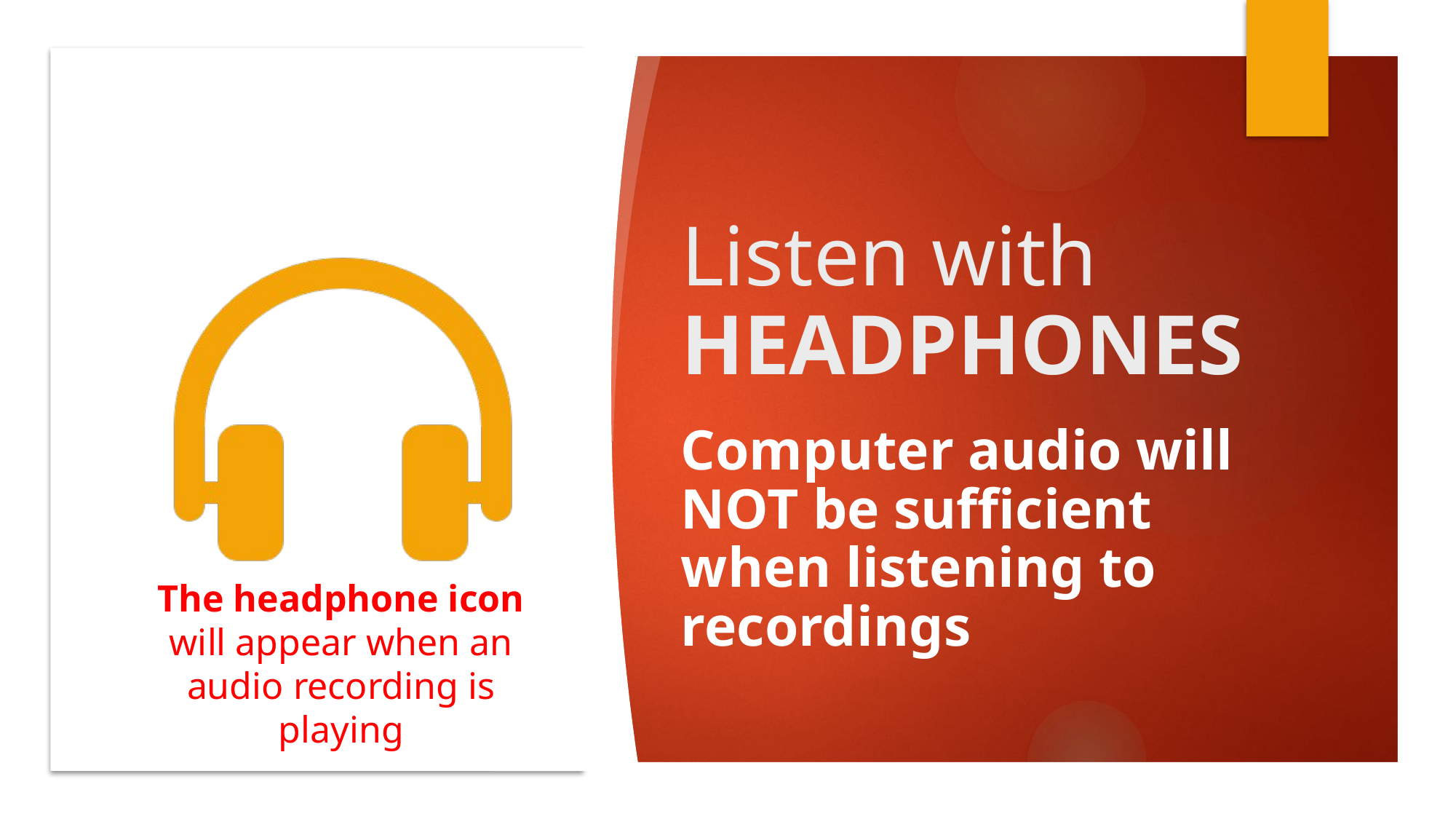

# Listen with HEADPHONES
Computer audio will NOT be sufficient when listening to recordings
The headphone icon will appear when an audio recording is playing

## Slide 14
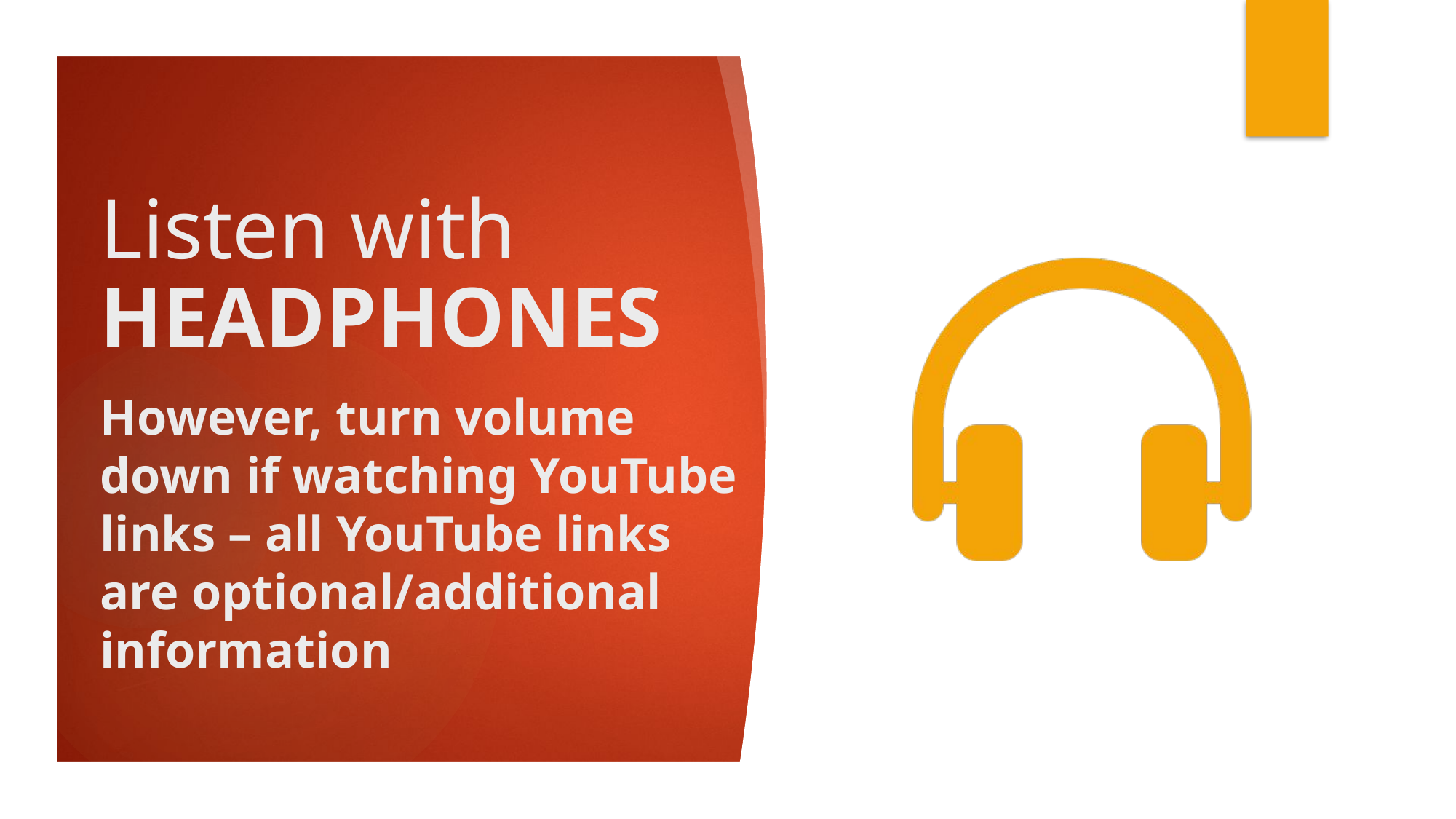

Listen with HEADPHONES
# However, turn volume down if watching YouTube links – all YouTube links are optional/additional information

## Slide 15
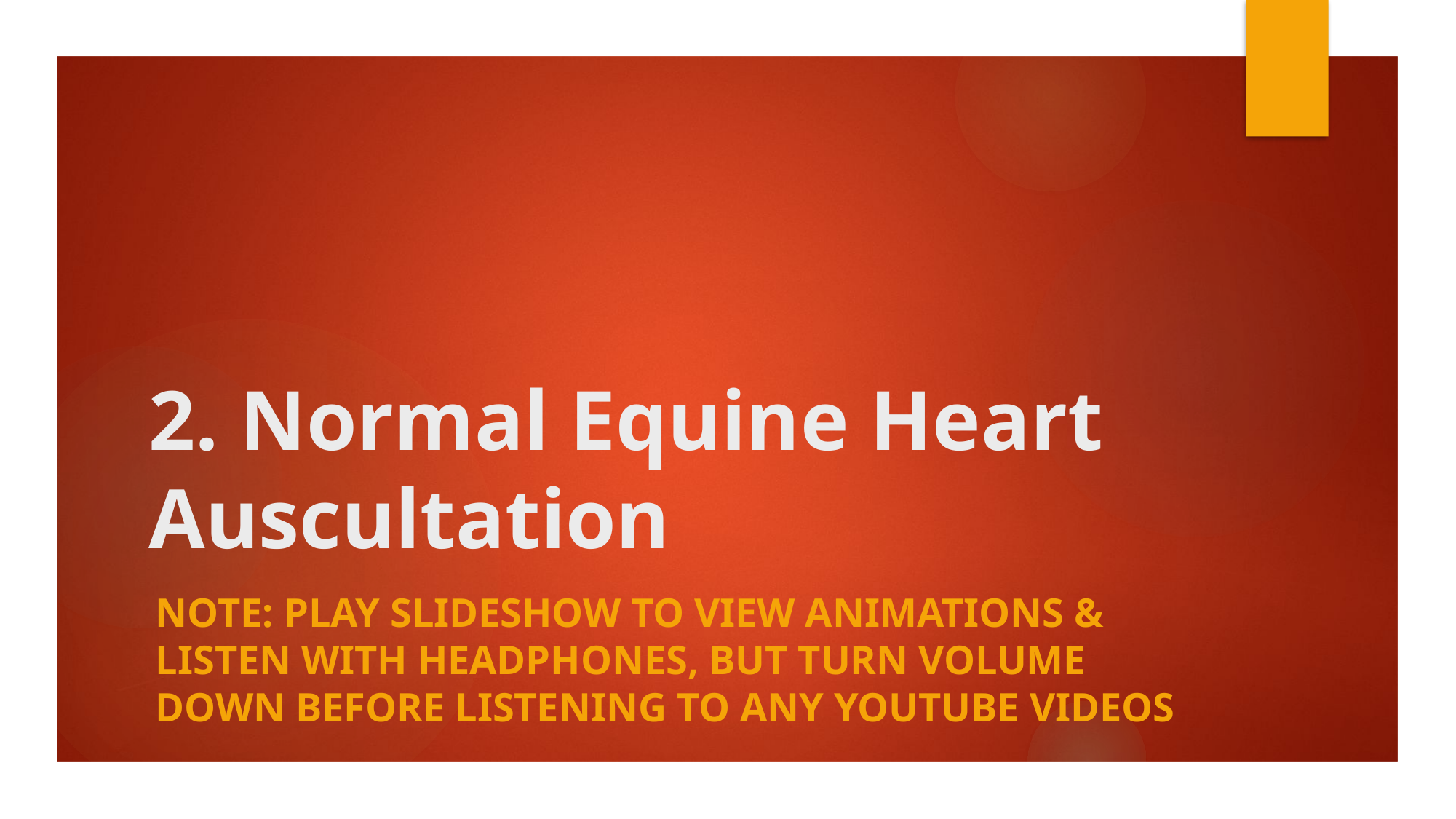

# 2. Normal Equine Heart Auscultation
Note: Play Slideshow to view animations & Listen with Headphones, but turn volume down before listening to any Youtube Videos

## Slide 16
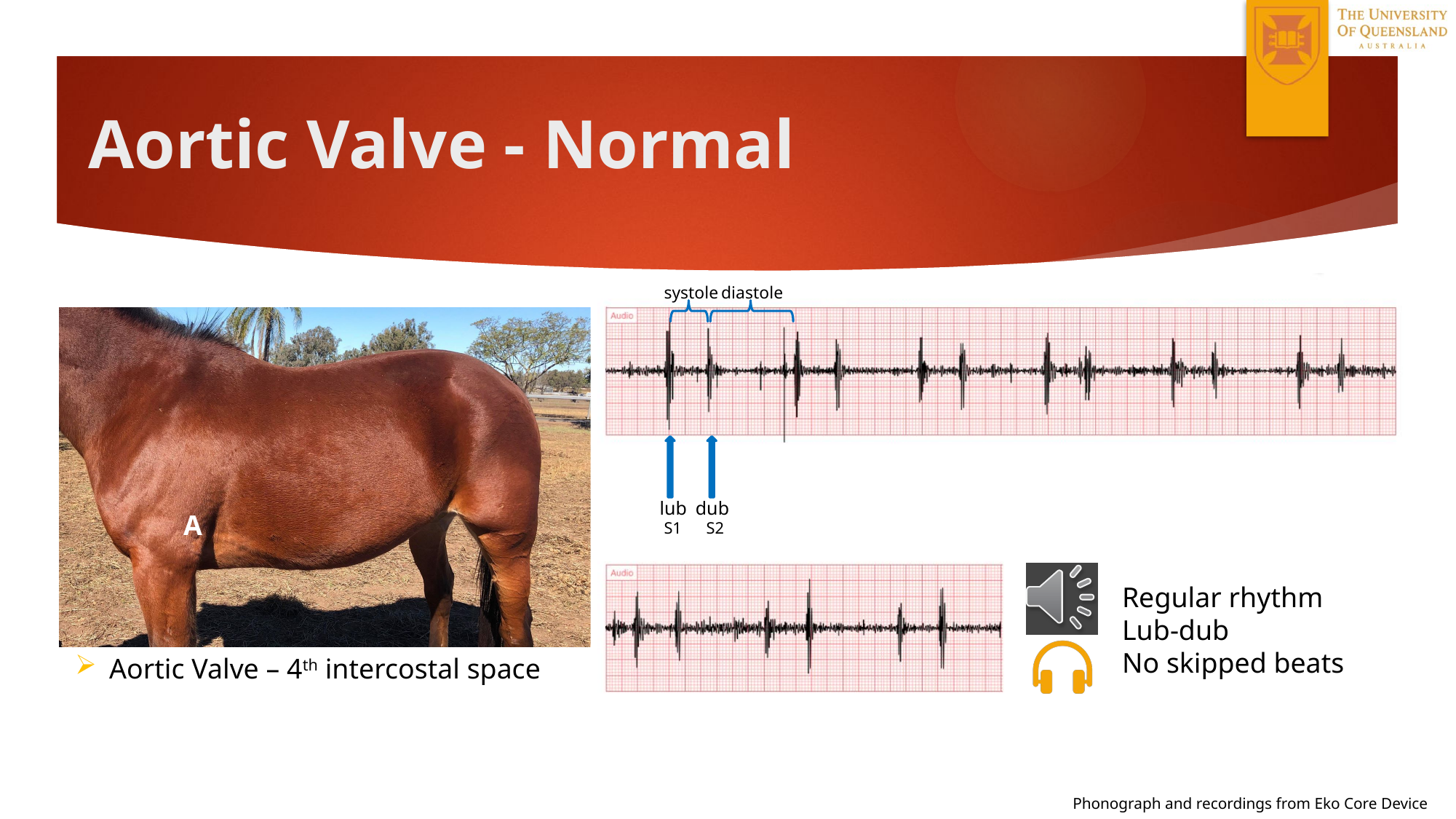

# Aortic Valve - Normal
diastole
systole
lub
dub
A
S1
S2
Regular rhythm
Lub-dub
No skipped beats
Aortic Valve – 4th intercostal space
Phonograph and recordings from Eko Core Device

## Slide 17
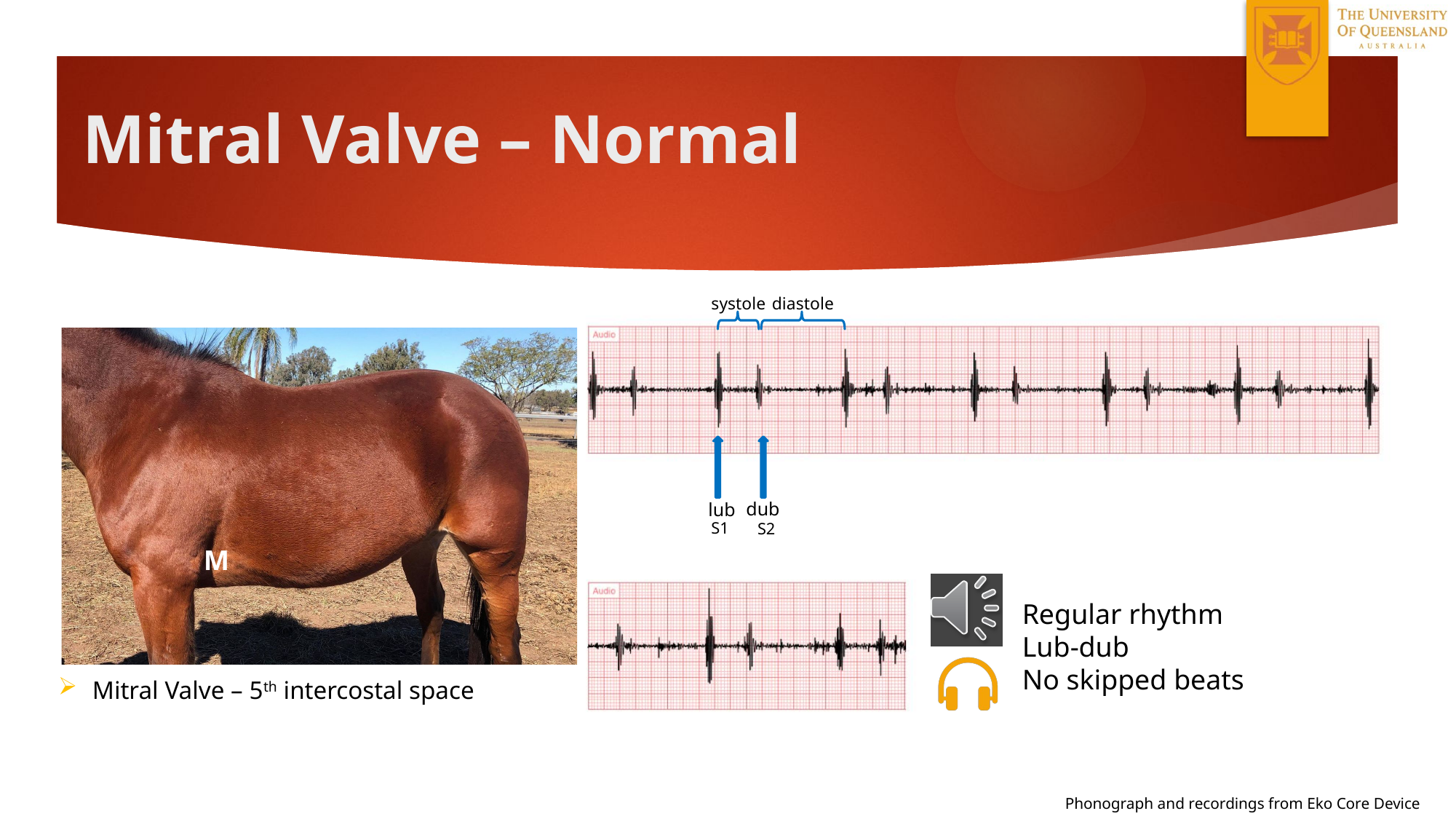

# Mitral Valve – Normal
systole
diastole
dub
lub
S1
S2
M
Regular rhythm
Lub-dub
No skipped beats
Mitral Valve – 5th intercostal space
Phonograph and recordings from Eko Core Device

## Slide 18
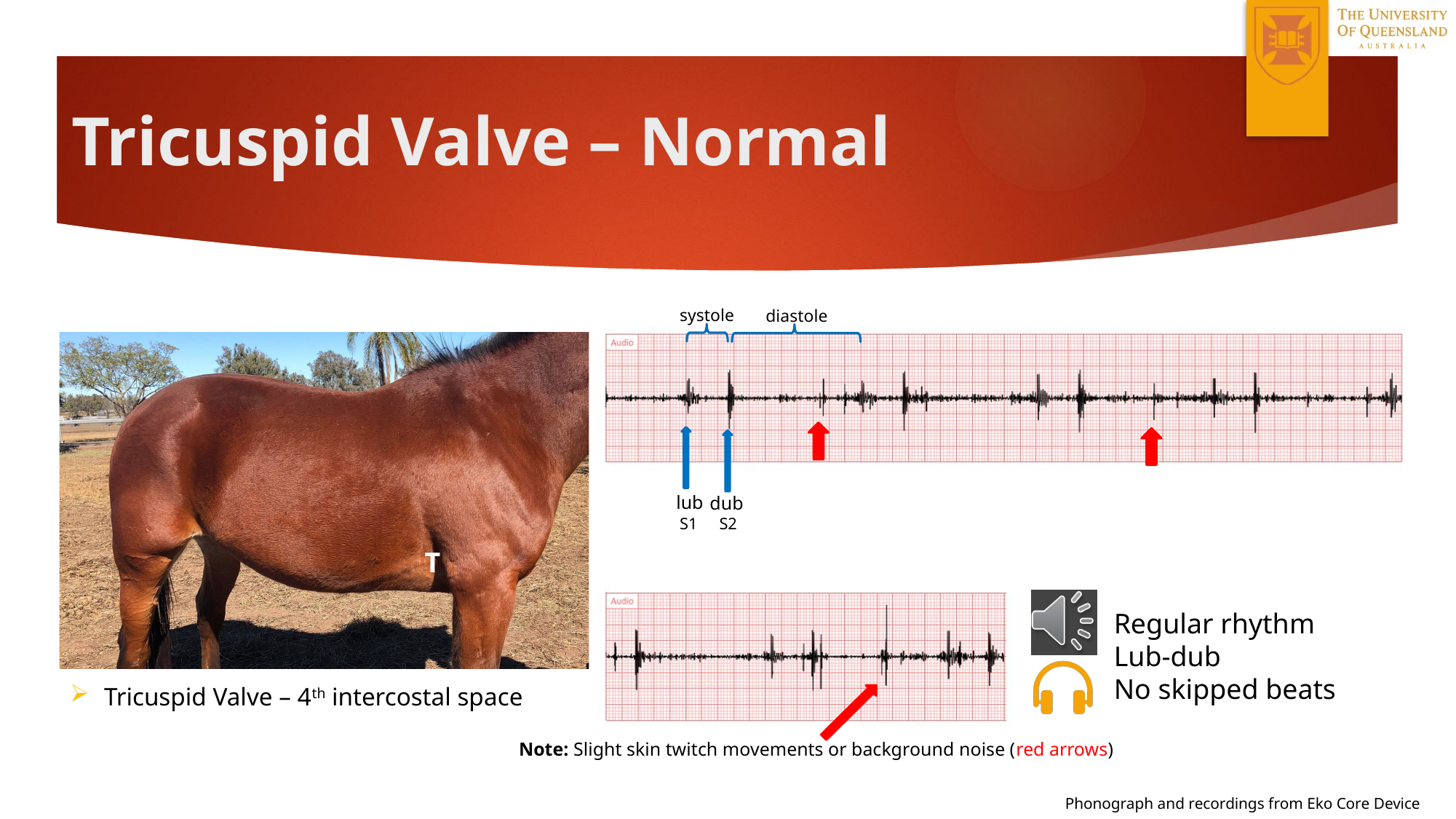

# Tricuspid Valve – Normal
systole
diastole
lub
dub
S1
S2
T
Regular rhythm
Lub-dub
No skipped beats
Tricuspid Valve – 4th intercostal space
Note: Slight skin twitch movements or background noise (red arrows)
Phonograph and recordings from Eko Core Device

## Slide 19
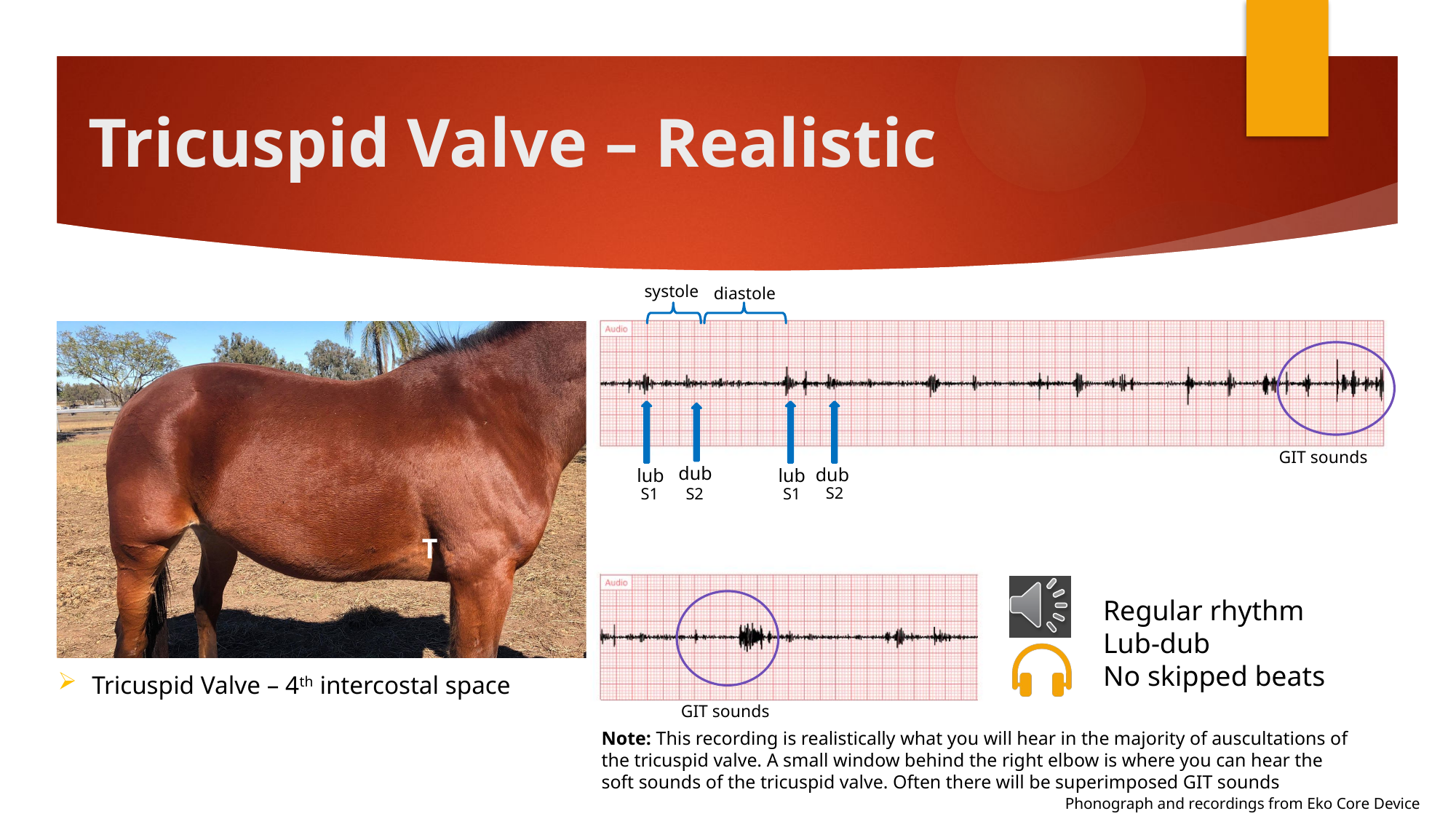

# Tricuspid Valve – Realistic
systole
diastole
GIT sounds
dub
dub
lub
lub
S2
S1
S2
S1
T
Regular rhythm
Lub-dub
No skipped beats
Tricuspid Valve – 4th intercostal space
GIT sounds
Note: This recording is realistically what you will hear in the majority of auscultations of the tricuspid valve. A small window behind the right elbow is where you can hear the soft sounds of the tricuspid valve. Often there will be superimposed GIT sounds
Phonograph and recordings from Eko Core Device

## Slide 20
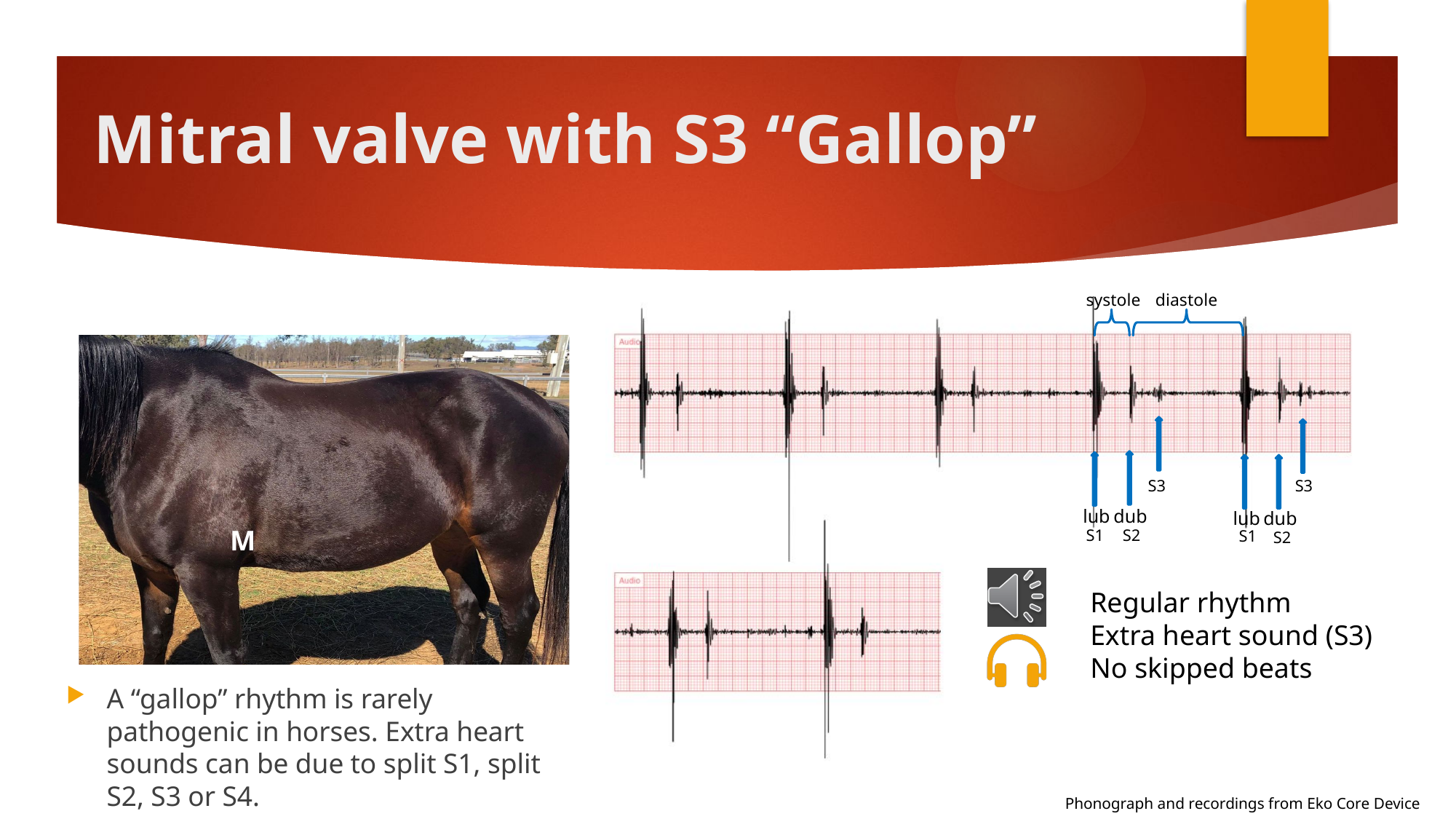

# Mitral valve with S3 “Gallop”
systole
diastole
S3
S3
lub
dub
dub
lub
M
S1
S2
S1
S2
Regular rhythm
Extra heart sound (S3)
No skipped beats
A “gallop” rhythm is rarely pathogenic in horses. Extra heart sounds can be due to split S1, split S2, S3 or S4.
Phonograph and recordings from Eko Core Device

## Slide 21
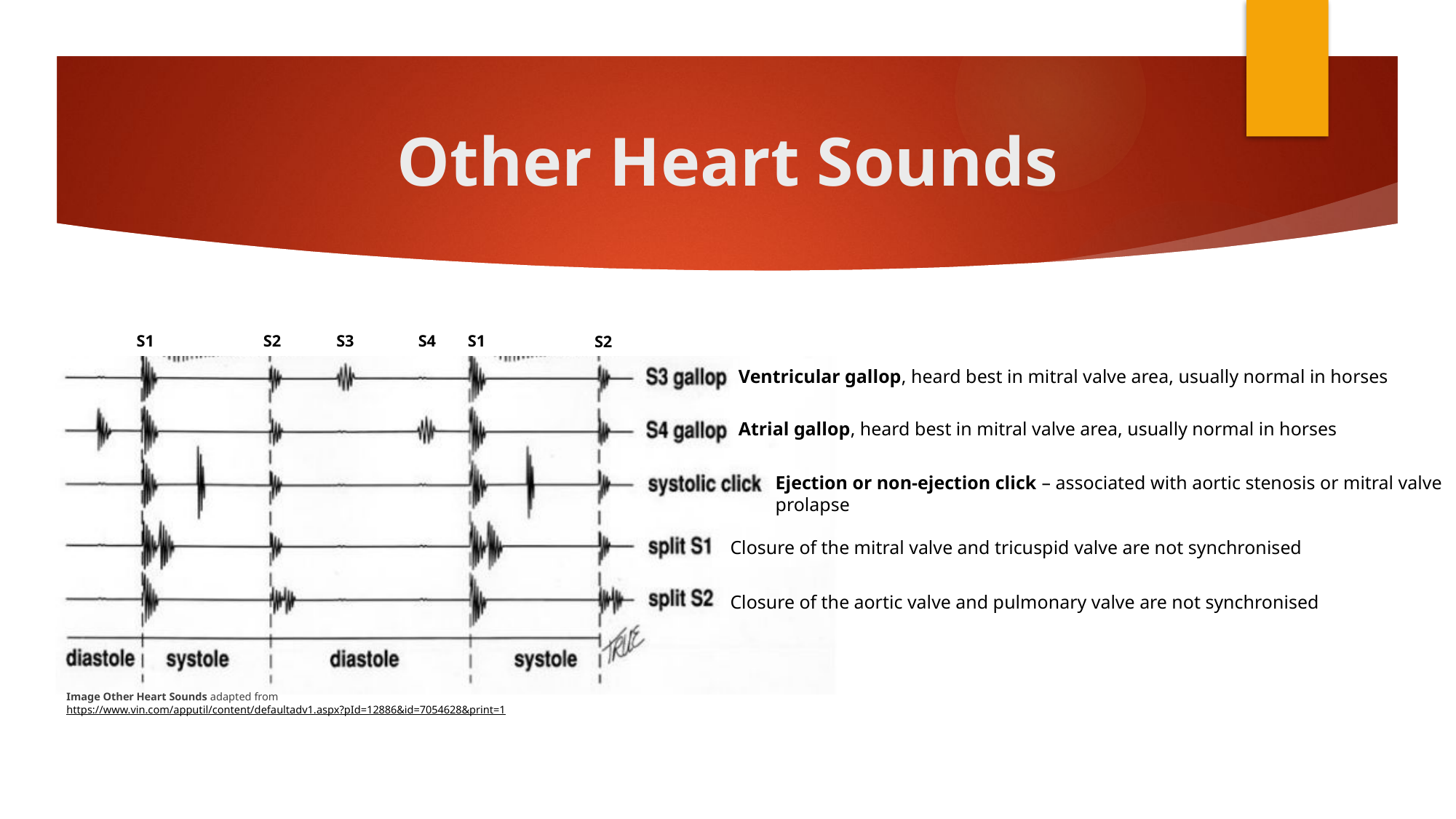

# Other Heart Sounds
S1
S2
S3
S4
S1
S2
Ventricular gallop, heard best in mitral valve area, usually normal in horses
Atrial gallop, heard best in mitral valve area, usually normal in horses
Ejection or non-ejection click – associated with aortic stenosis or mitral valve prolapse
Closure of the mitral valve and tricuspid valve are not synchronised
Closure of the aortic valve and pulmonary valve are not synchronised
Image Other Heart Sounds adapted from https://www.vin.com/apputil/content/defaultadv1.aspx?pId=12886&id=7054628&print=1

## Slide 22
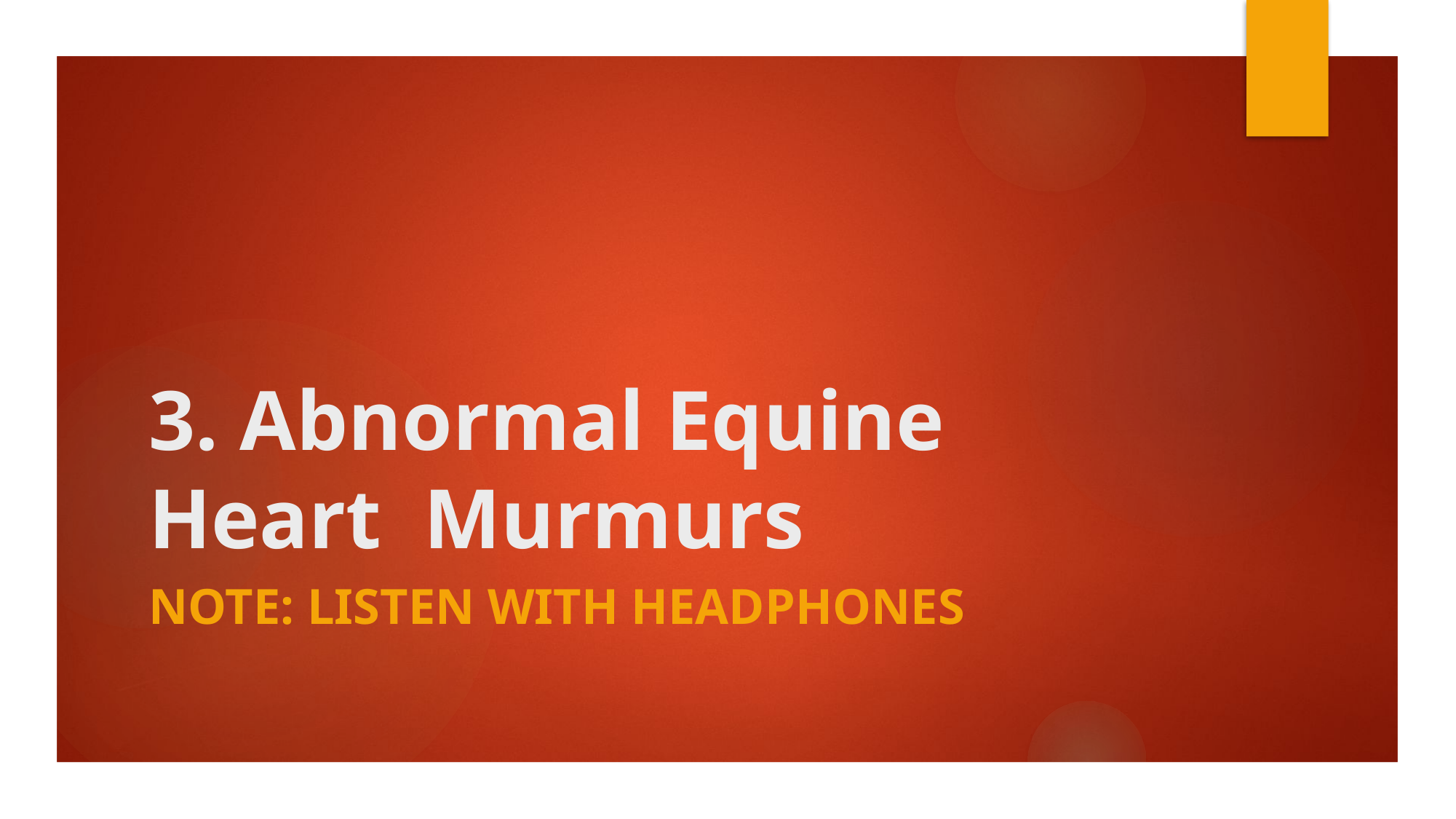

# 3. Abnormal Equine Heart Murmurs
Note: Listen with HeadPhones

## Slide 23
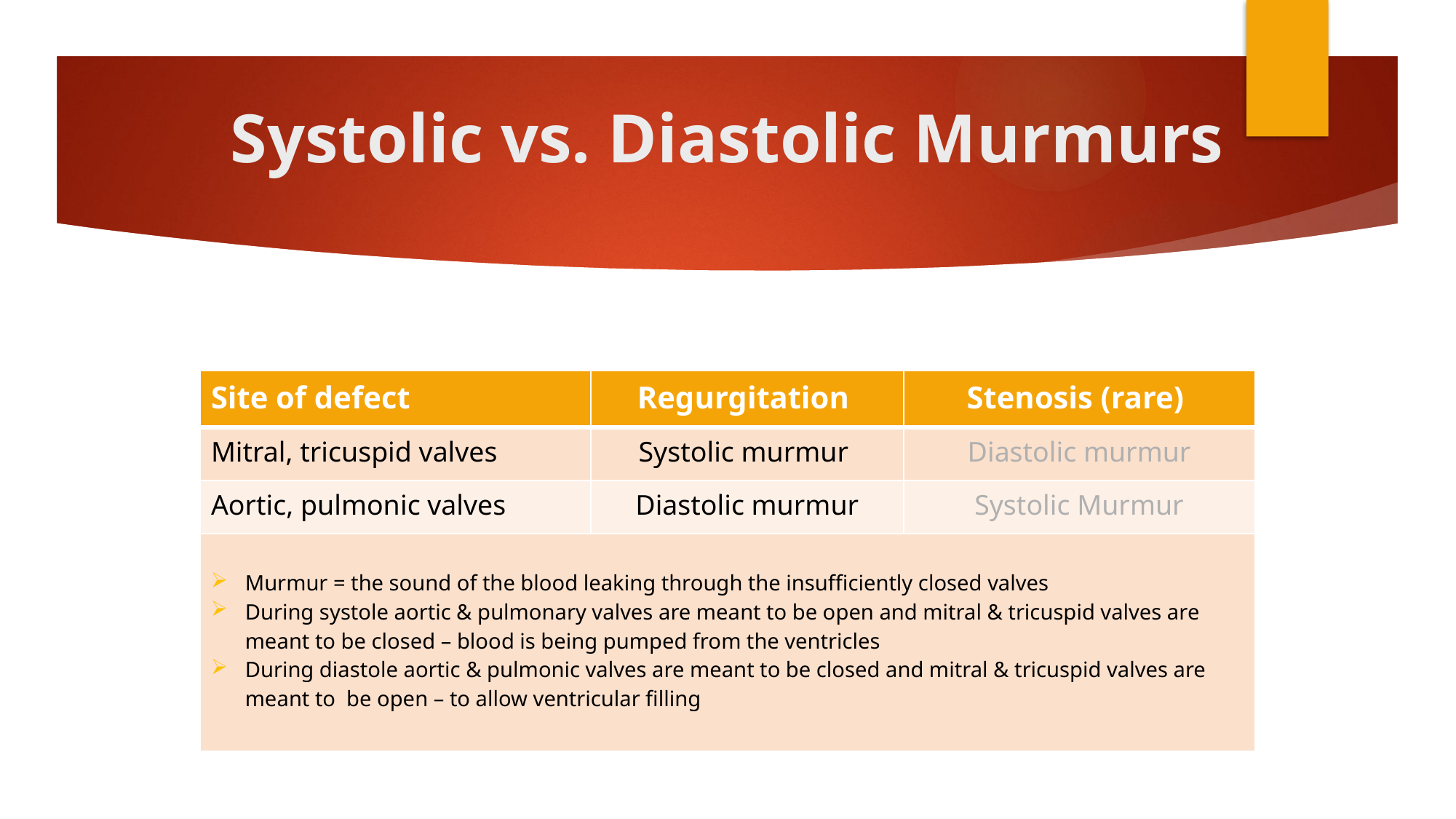

# Systolic vs. Diastolic Murmurs
| Site of defect | Regurgitation | Stenosis (rare) |
| --- | --- | --- |
| Mitral, tricuspid valves | Systolic murmur | Diastolic murmur |
| Aortic, pulmonic valves | Diastolic murmur | Systolic Murmur |
| Murmur = the sound of the blood leaking through the insufficiently closed valves During systole aortic & pulmonary valves are meant to be open and mitral & tricuspid valves are meant to be closed – blood is being pumped from the ventricles During diastole aortic & pulmonic valves are meant to be closed and mitral & tricuspid valves are meant to be open – to allow ventricular filling | | |

## Slide 24
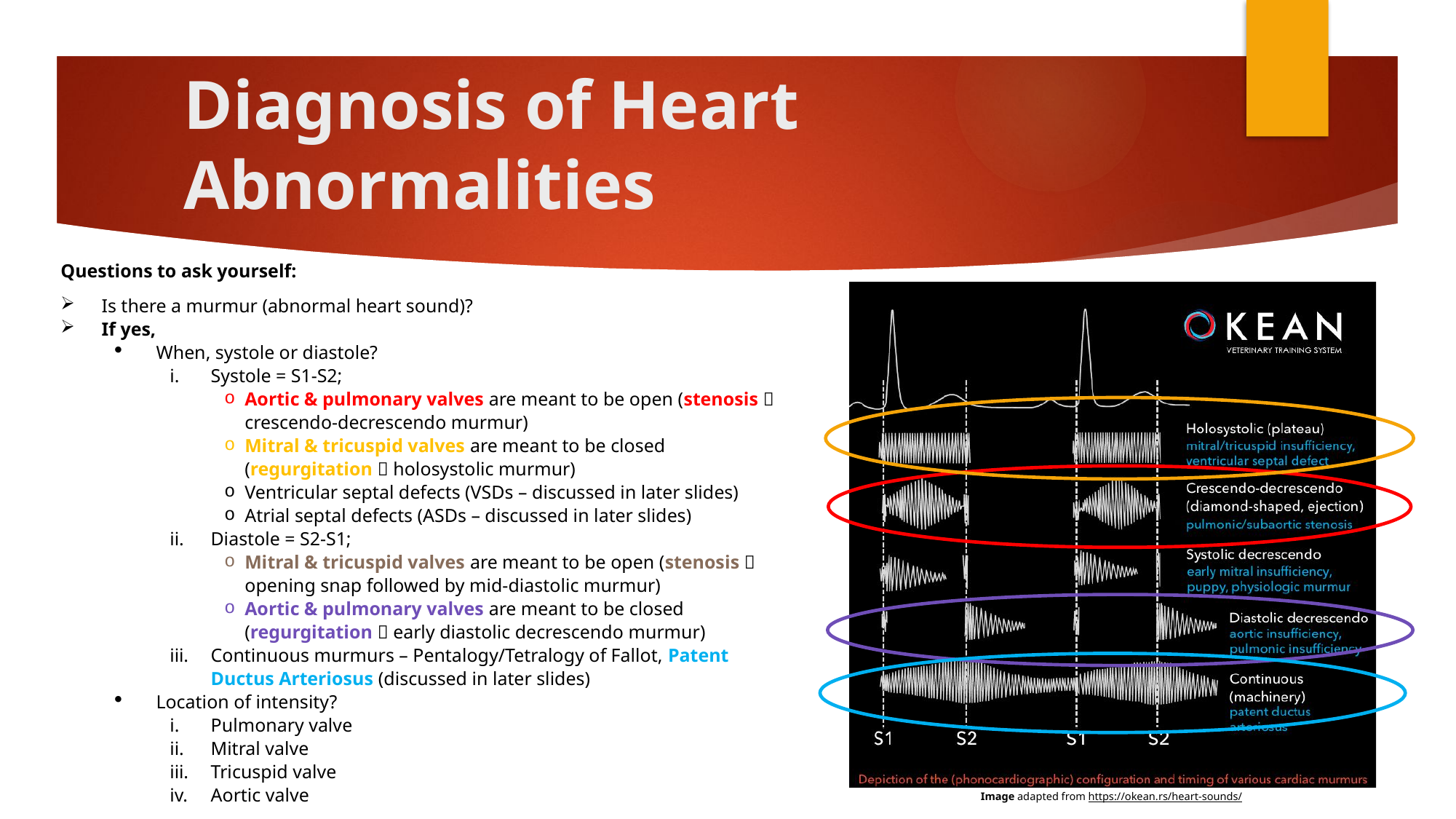

# Diagnosis of Heart Abnormalities
Questions to ask yourself:
Is there a murmur (abnormal heart sound)?
If yes,
When, systole or diastole?
Systole = S1-S2;
Aortic & pulmonary valves are meant to be open (stenosis  crescendo-decrescendo murmur)
Mitral & tricuspid valves are meant to be closed (regurgitation  holosystolic murmur)
Ventricular septal defects (VSDs – discussed in later slides)
Atrial septal defects (ASDs – discussed in later slides)
Diastole = S2-S1;
Mitral & tricuspid valves are meant to be open (stenosis  opening snap followed by mid-diastolic murmur)
Aortic & pulmonary valves are meant to be closed (regurgitation  early diastolic decrescendo murmur)
Continuous murmurs – Pentalogy/Tetralogy of Fallot, Patent Ductus Arteriosus (discussed in later slides)
Location of intensity?
Pulmonary valve
Mitral valve
Tricuspid valve
Aortic valve
Image adapted from https://okean.rs/heart-sounds/

## Slide 25
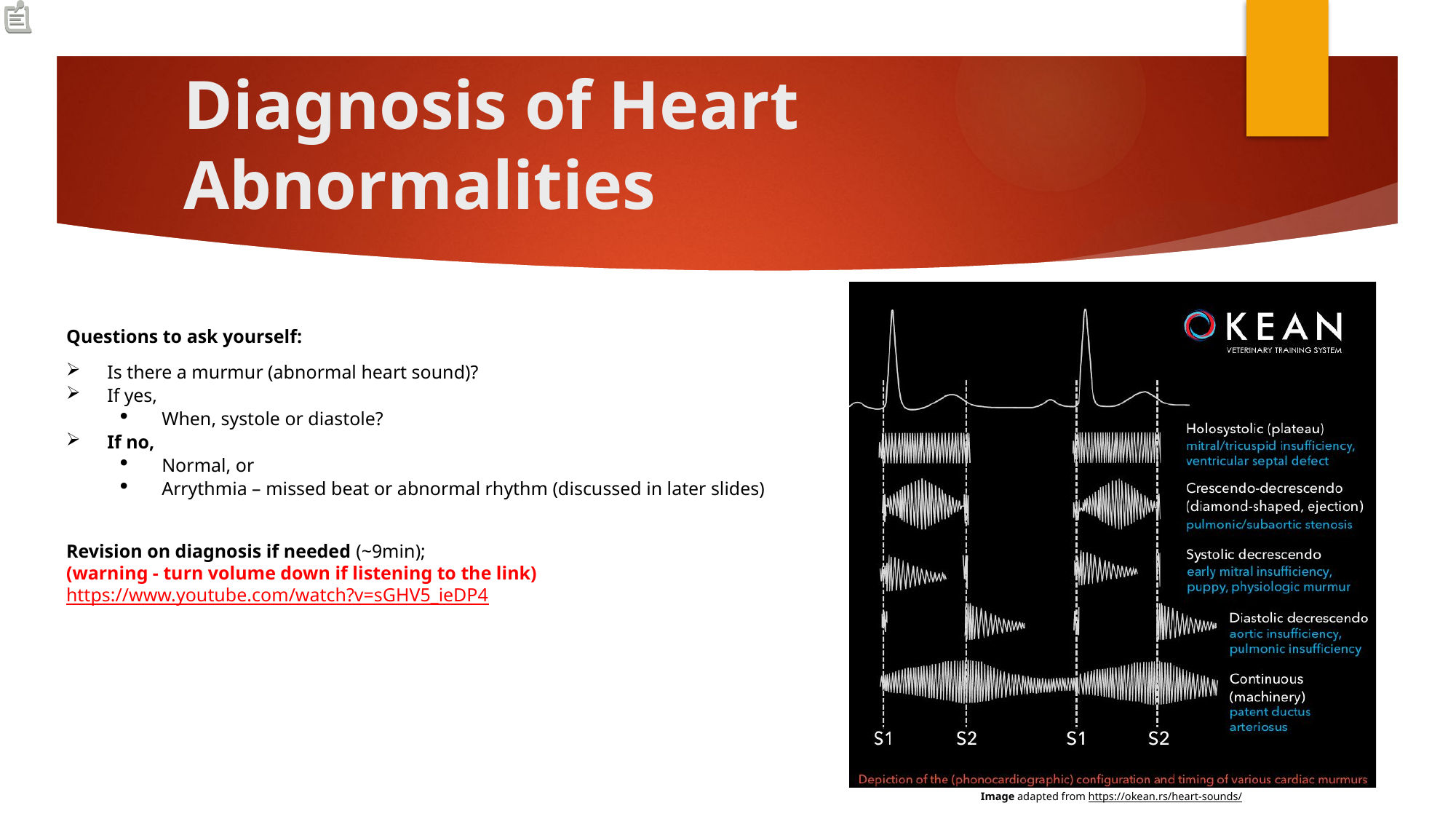

# Diagnosis of Heart Abnormalities
Questions to ask yourself:
Is there a murmur (abnormal heart sound)?
If yes,
When, systole or diastole?
If no,
Normal, or
Arrythmia – missed beat or abnormal rhythm (discussed in later slides)
Revision on diagnosis if needed (~9min);
(warning - turn volume down if listening to the link)
https://www.youtube.com/watch?v=sGHV5_ieDP4
Image adapted from https://okean.rs/heart-sounds/

## Slide 26
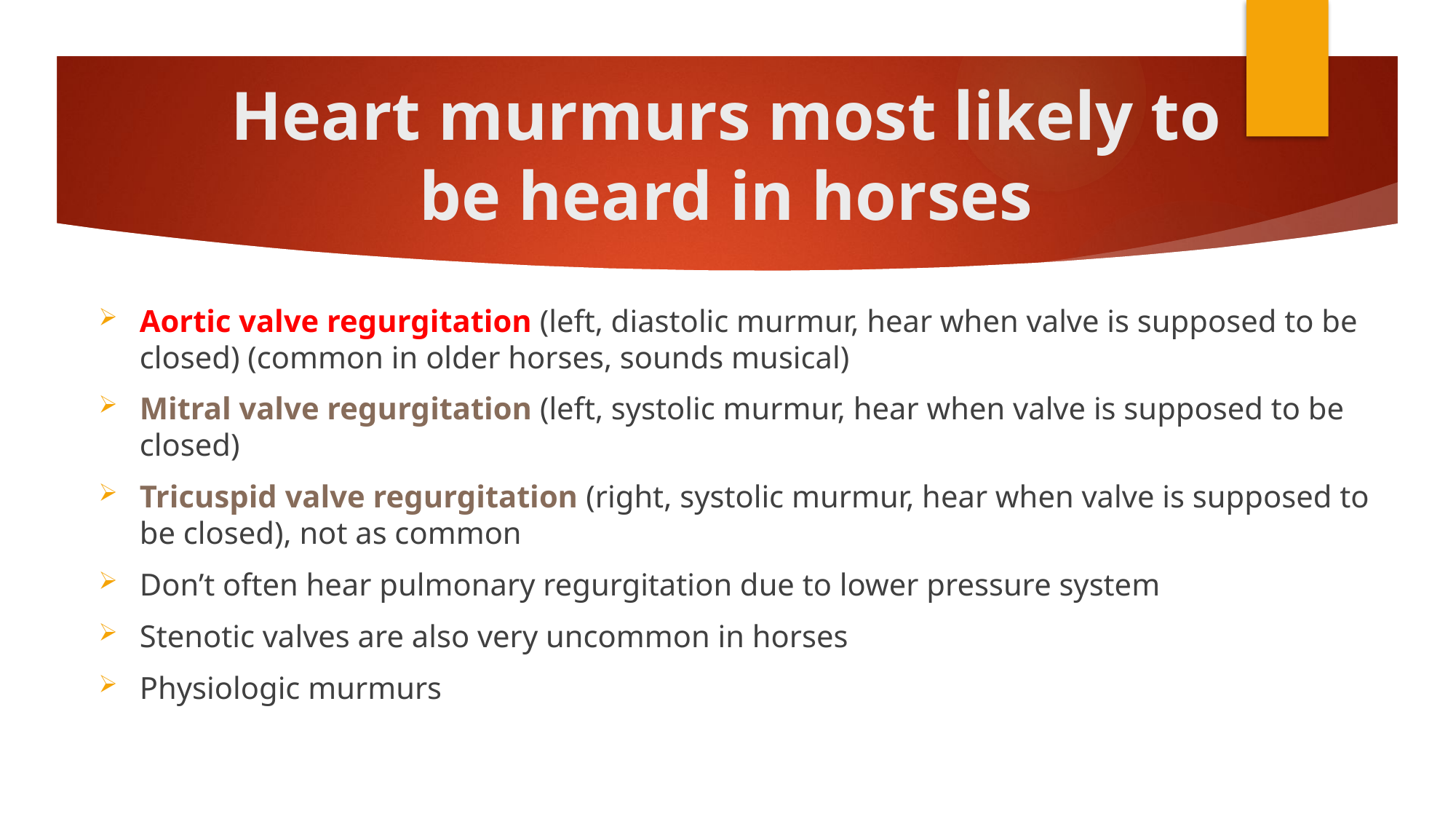

# Heart murmurs most likely to be heard in horses
Aortic valve regurgitation (left, diastolic murmur, hear when valve is supposed to be closed) (common in older horses, sounds musical)
Mitral valve regurgitation (left, systolic murmur, hear when valve is supposed to be closed)
Tricuspid valve regurgitation (right, systolic murmur, hear when valve is supposed to be closed), not as common
Don’t often hear pulmonary regurgitation due to lower pressure system
Stenotic valves are also very uncommon in horses
Physiologic murmurs

## Slide 27
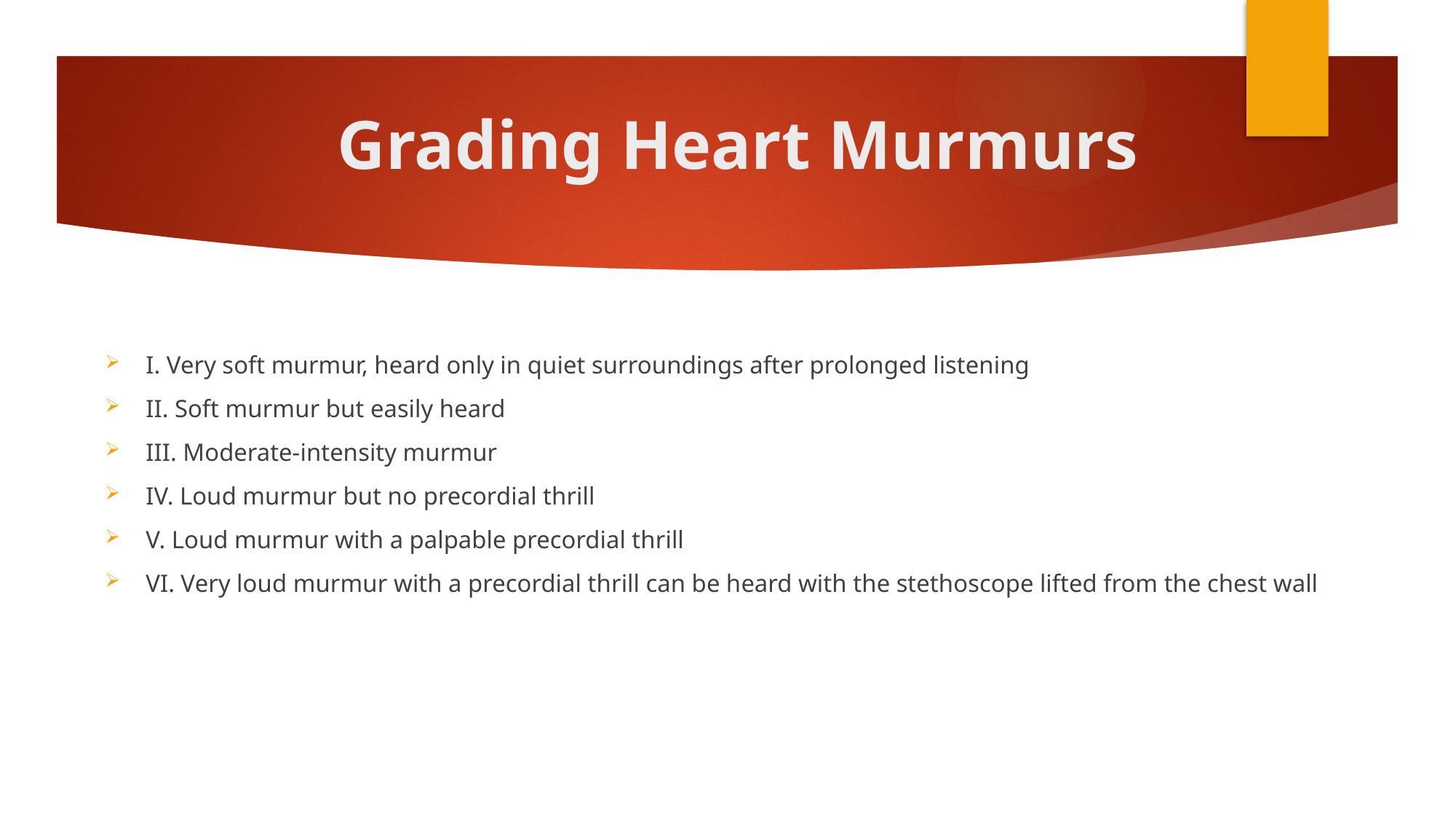

# Grading Heart Murmurs
I. Very soft murmur, heard only in quiet surroundings after prolonged listening
II. Soft murmur but easily heard
III. Moderate-intensity murmur
IV. Loud murmur but no precordial thrill
V. Loud murmur with a palpable precordial thrill
VI. Very loud murmur with a precordial thrill can be heard with the stethoscope lifted from the chest wall

## Slide 28
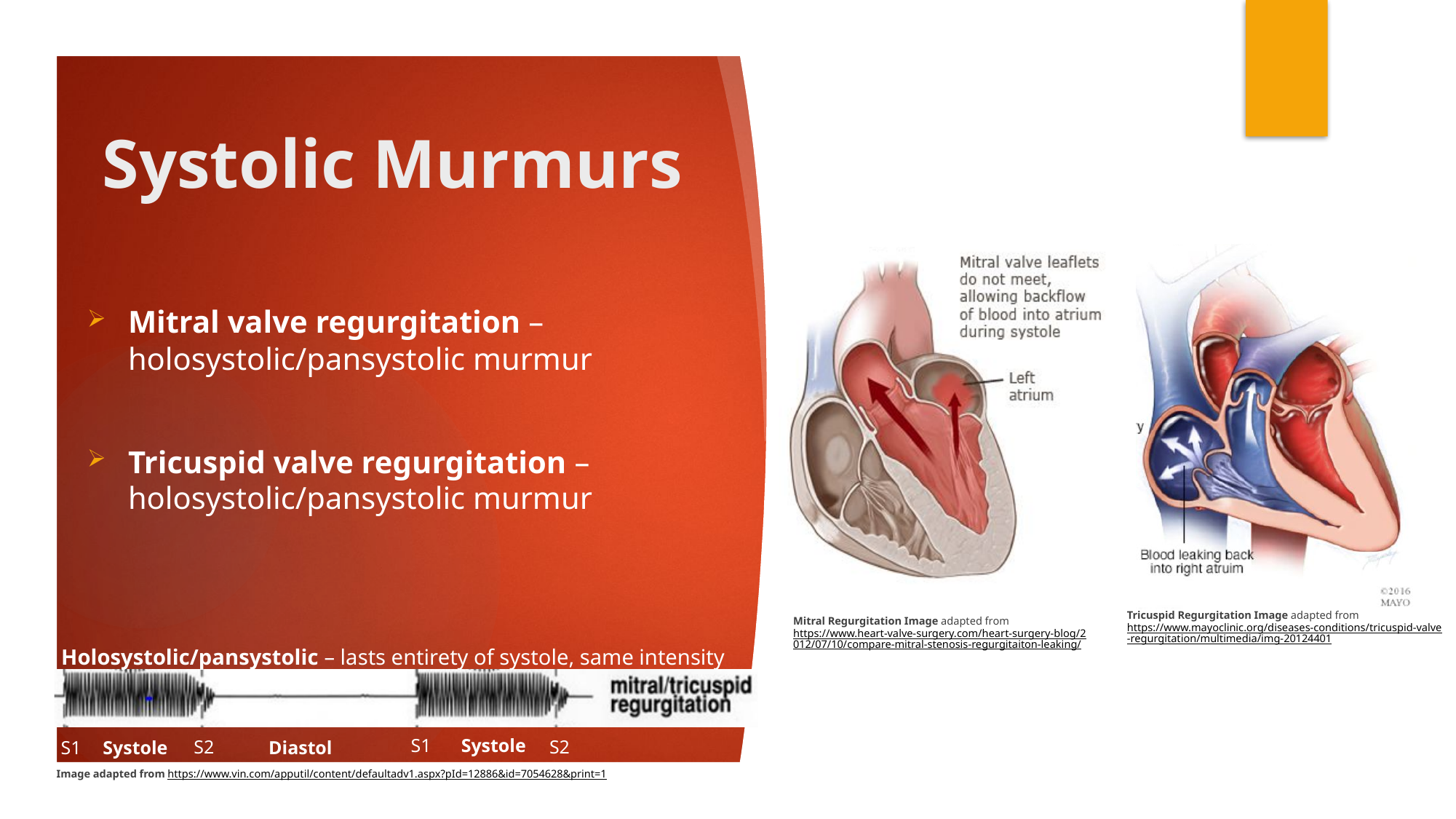

# Systolic Murmurs
Mitral valve regurgitation – holosystolic/pansystolic murmur
Tricuspid valve regurgitation – holosystolic/pansystolic murmur
Tricuspid Regurgitation Image adapted from https://www.mayoclinic.org/diseases-conditions/tricuspid-valve-regurgitation/multimedia/img-20124401
Mitral Regurgitation Image adapted from https://www.heart-valve-surgery.com/heart-surgery-blog/2012/07/10/compare-mitral-stenosis-regurgitaiton-leaking/
Holosystolic/pansystolic – lasts entirety of systole, same intensity
S1
Systole
S2
S2
S1
Systole
Diastole
Image adapted from https://www.vin.com/apputil/content/defaultadv1.aspx?pId=12886&id=7054628&print=1

## Slide 29
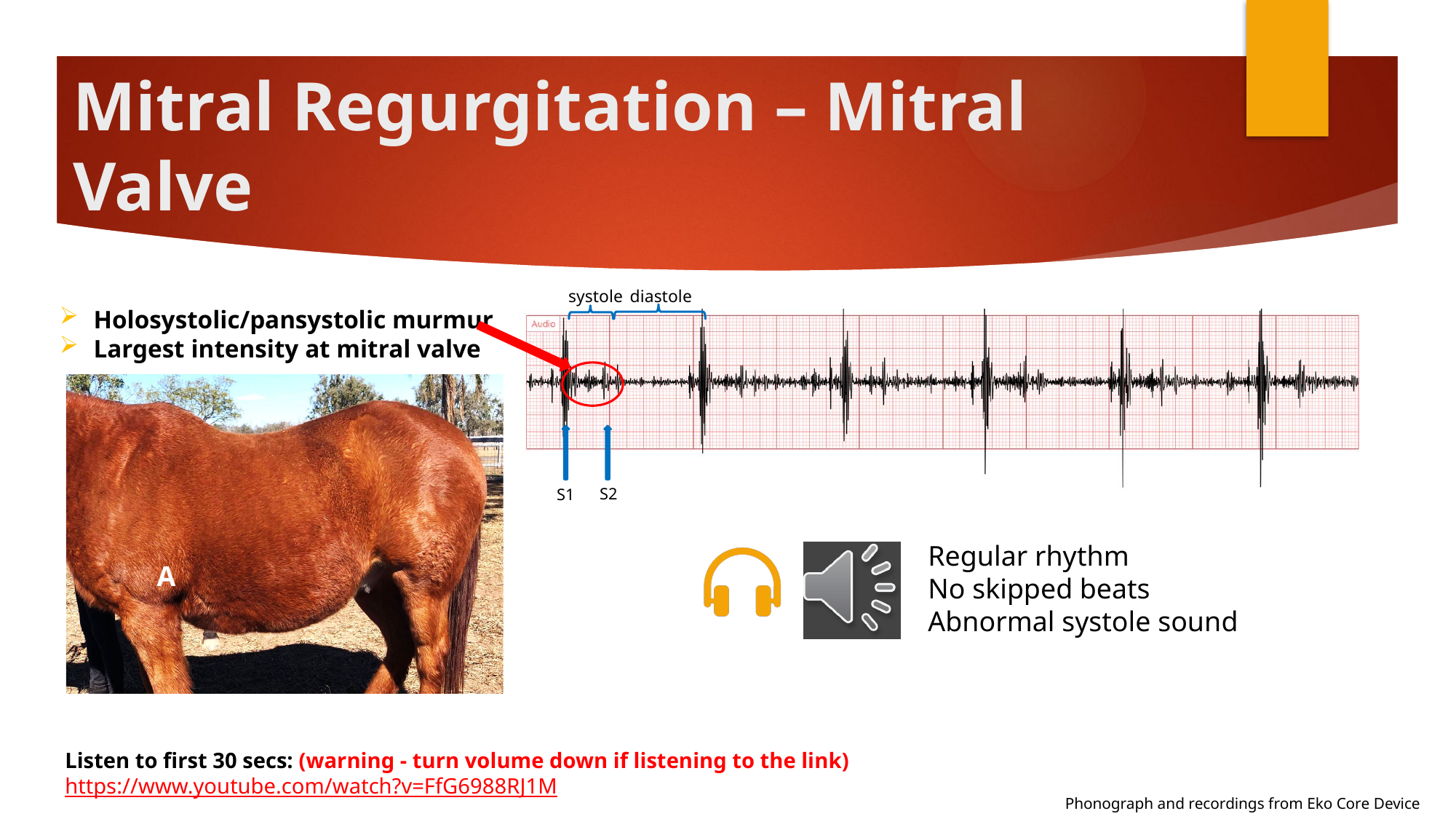

# Mitral Regurgitation – Mitral Valve
systole
diastole
Holosystolic/pansystolic murmur
Largest intensity at mitral valve
S2
S1
Regular rhythm
No skipped beats
Abnormal systole sound
A
Listen to first 30 secs: (warning - turn volume down if listening to the link)
https://www.youtube.com/watch?v=FfG6988RJ1M
Phonograph and recordings from Eko Core Device

## Slide 30
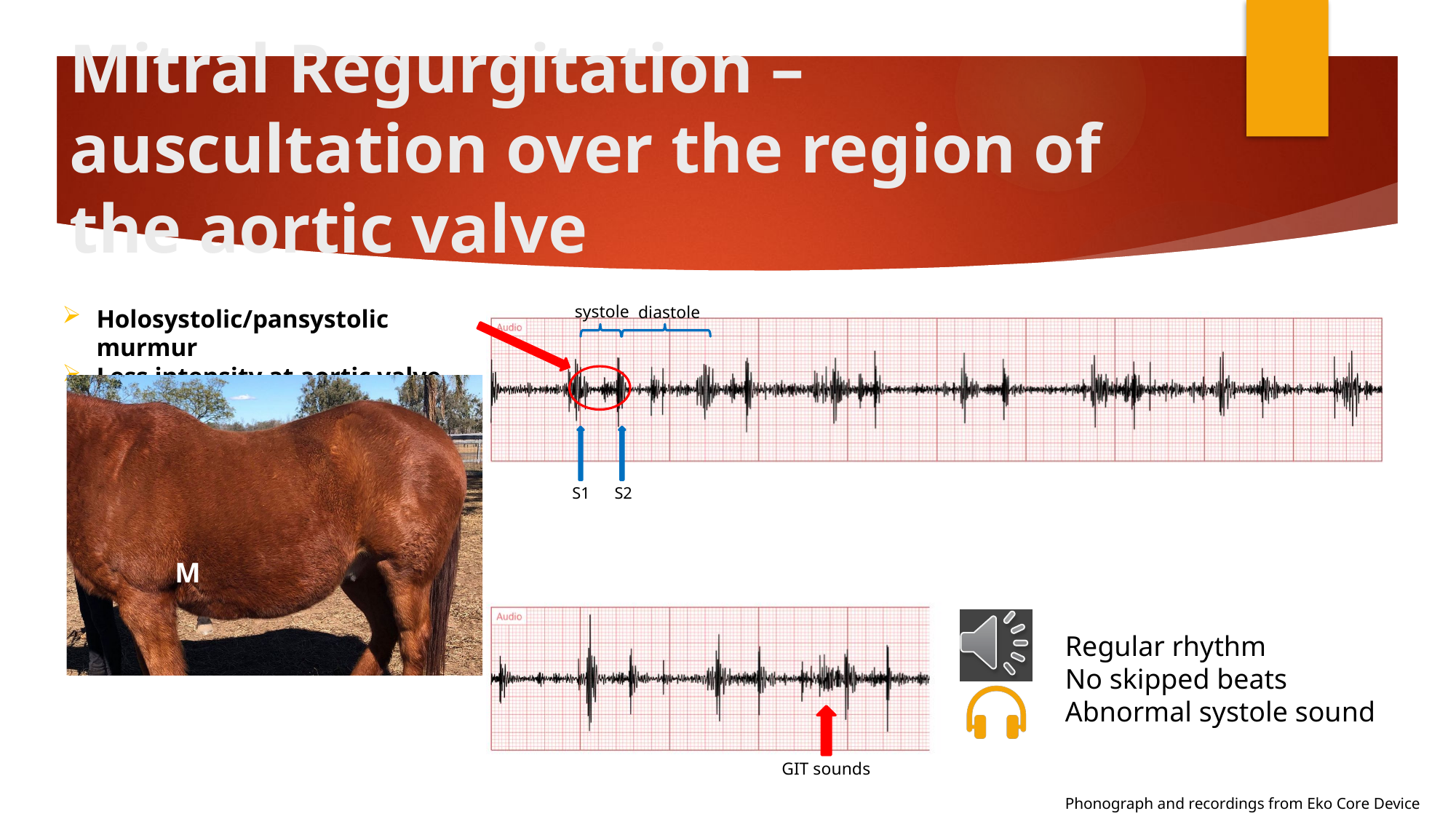

# Mitral Regurgitation – auscultation over the region of the aortic valve
systole
diastole
Holosystolic/pansystolic murmur
Less intensity at aortic valve
S1
S2
M
Regular rhythm
No skipped beats
Abnormal systole sound
GIT sounds
Phonograph and recordings from Eko Core Device

## Slide 31
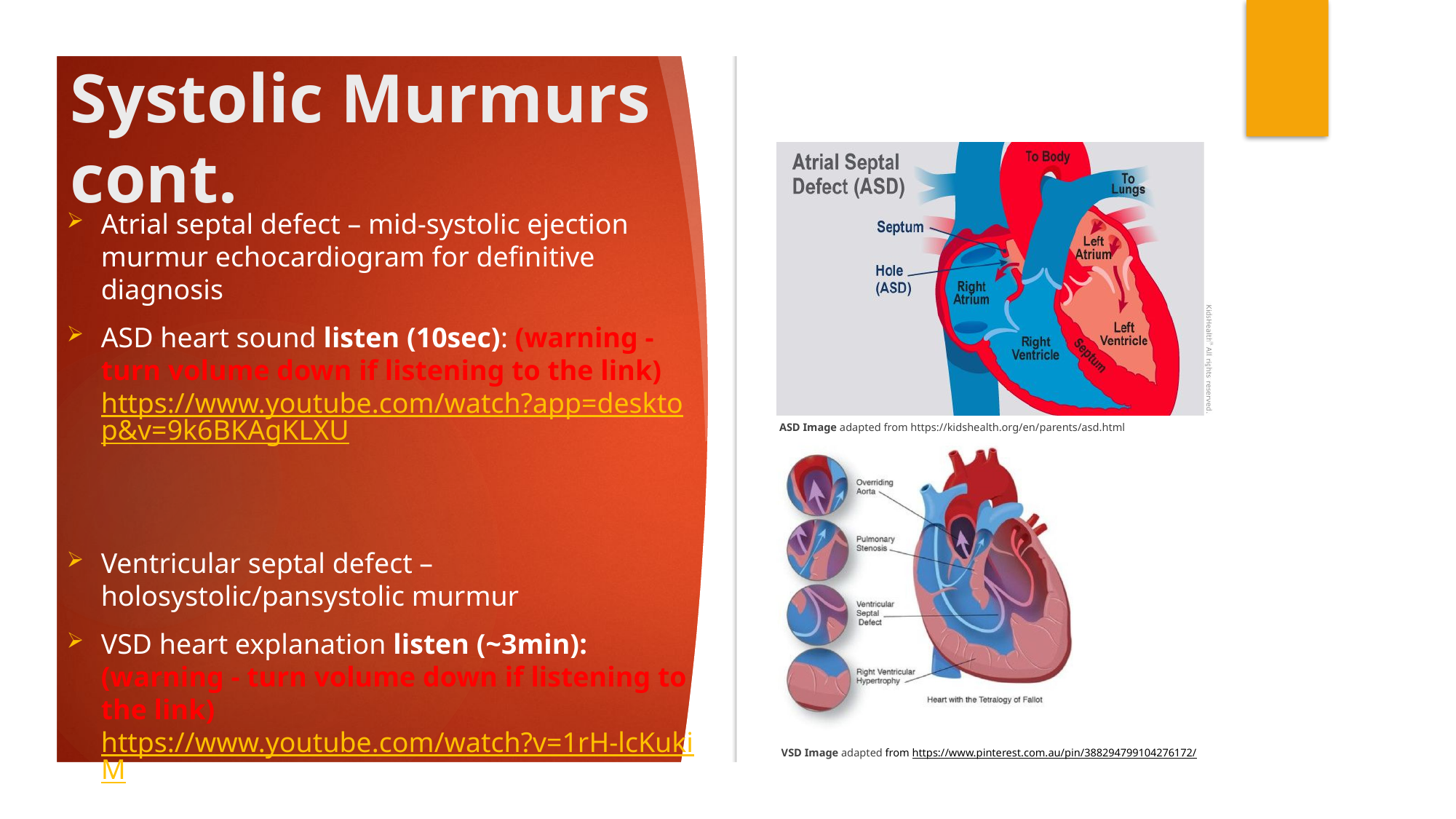

# Systolic Murmurs cont.
Atrial septal defect – mid-systolic ejection murmur echocardiogram for definitive diagnosis
ASD heart sound listen (10sec): (warning - turn volume down if listening to the link) https://www.youtube.com/watch?app=desktop&v=9k6BKAgKLXU
Ventricular septal defect – holosystolic/pansystolic murmur
VSD heart explanation listen (~3min): (warning - turn volume down if listening to the link) https://www.youtube.com/watch?v=1rH-lcKukiM
ASD Image adapted from https://kidshealth.org/en/parents/asd.html
VSD Image adapted from https://www.pinterest.com.au/pin/388294799104276172/

## Slide 32
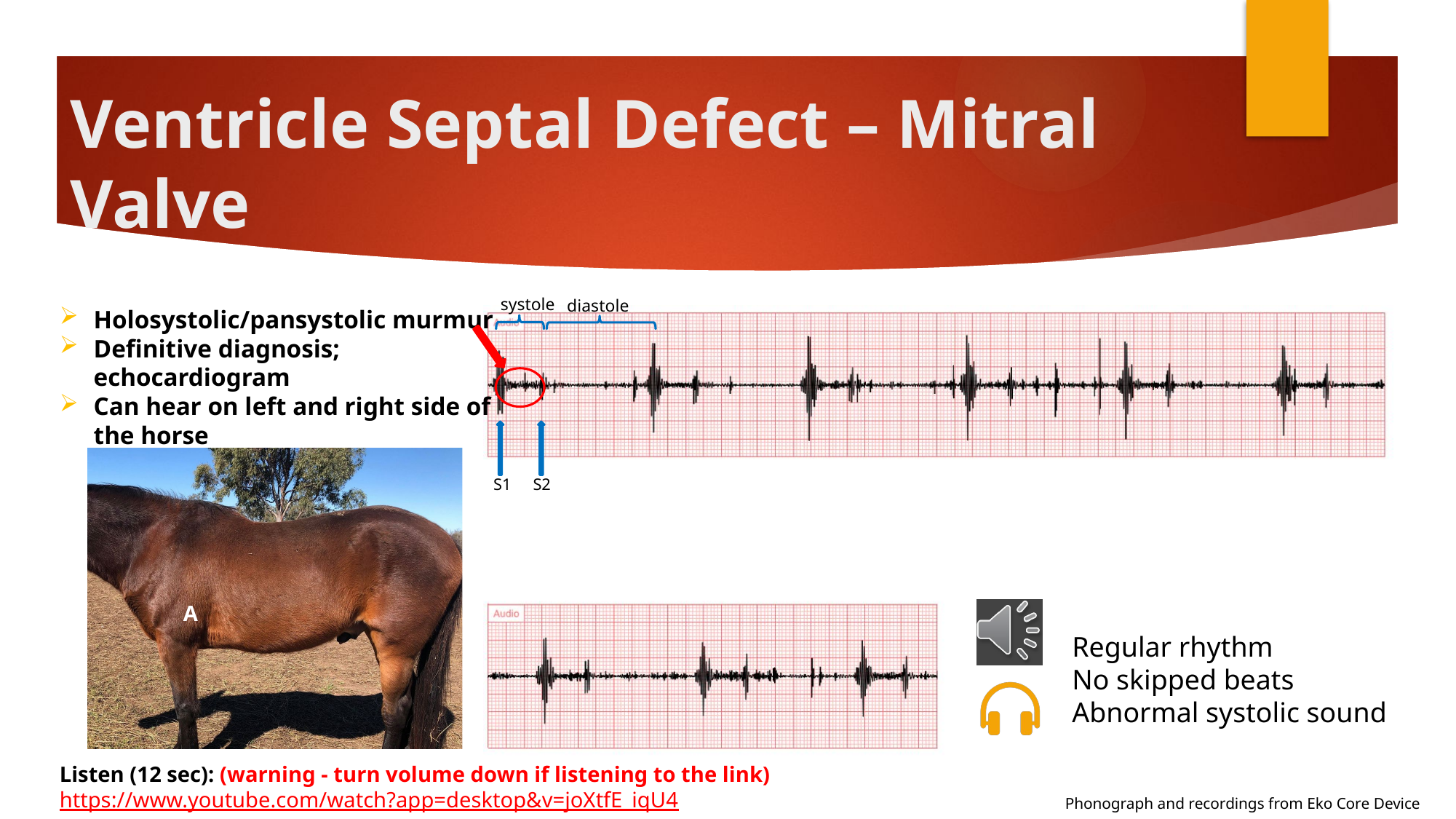

# Ventricle Septal Defect – Mitral Valve
systole
diastole
Holosystolic/pansystolic murmur
Definitive diagnosis; echocardiogram
Can hear on left and right side of the horse
S2
S1
A
Regular rhythm
No skipped beats
Abnormal systolic sound
Listen (12 sec): (warning - turn volume down if listening to the link)
https://www.youtube.com/watch?app=desktop&v=joXtfE_iqU4
Phonograph and recordings from Eko Core Device

## Slide 33
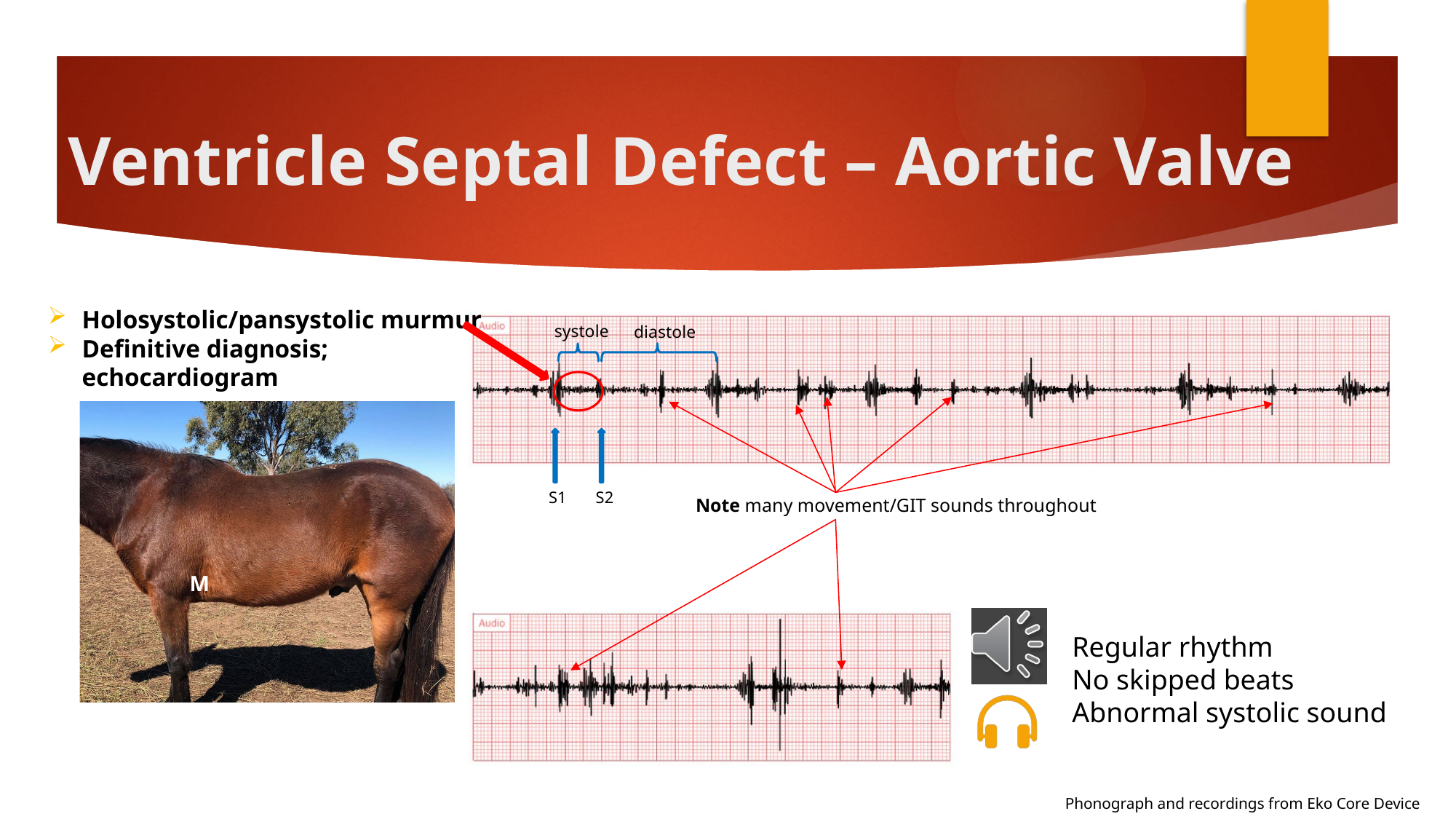

# Ventricle Septal Defect – Aortic Valve
Holosystolic/pansystolic murmur
Definitive diagnosis; echocardiogram
systole
diastole
S1
S2
Note many movement/GIT sounds throughout
M
Regular rhythm
No skipped beats
Abnormal systolic sound
Phonograph and recordings from Eko Core Device

## Slide 34
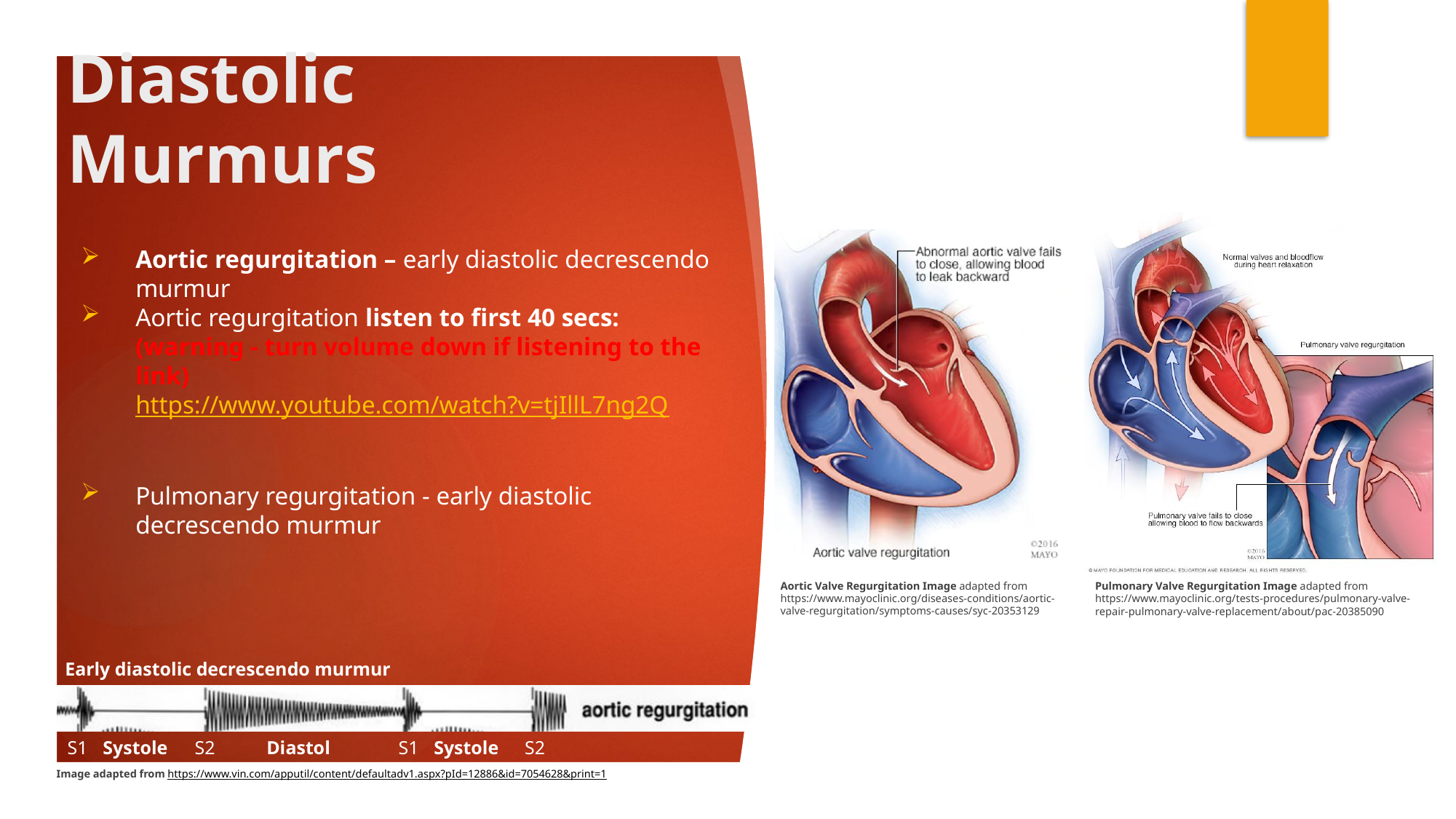

# Diastolic Murmurs
Aortic regurgitation – early diastolic decrescendo murmur
Aortic regurgitation listen to first 40 secs: (warning - turn volume down if listening to the link) https://www.youtube.com/watch?v=tjIllL7ng2Q
Pulmonary regurgitation - early diastolic decrescendo murmur
Aortic Valve Regurgitation Image adapted from https://www.mayoclinic.org/diseases-conditions/aortic-valve-regurgitation/symptoms-causes/syc-20353129
Pulmonary Valve Regurgitation Image adapted from https://www.mayoclinic.org/tests-procedures/pulmonary-valve-repair-pulmonary-valve-replacement/about/pac-20385090
Early diastolic decrescendo murmur
Diastole
S2
S2
Systole
Systole
S1
S1
Image adapted from https://www.vin.com/apputil/content/defaultadv1.aspx?pId=12886&id=7054628&print=1

## Slide 35
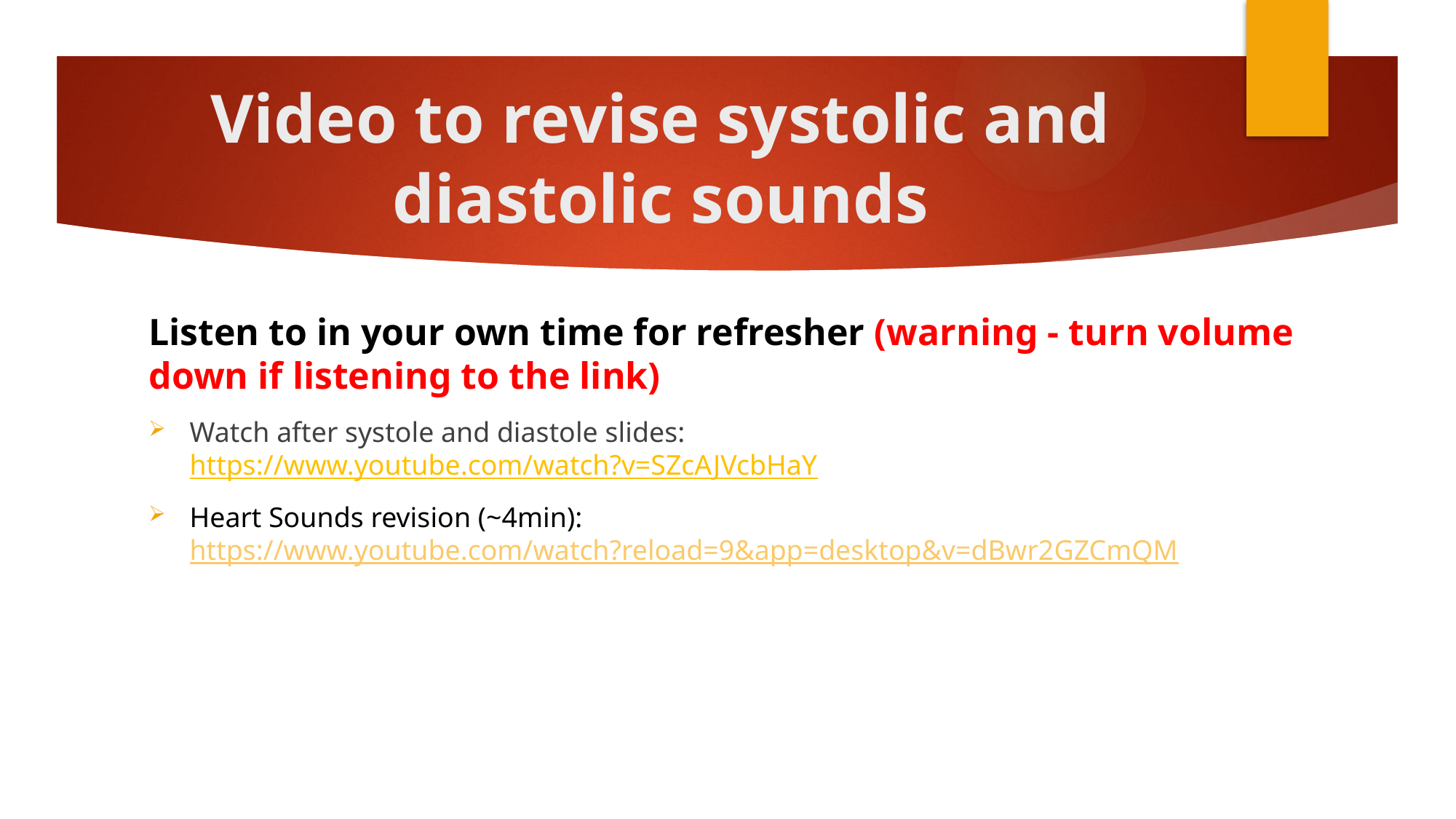

# Video to revise systolic and diastolic sounds
Listen to in your own time for refresher (warning - turn volume down if listening to the link)
Watch after systole and diastole slides: https://www.youtube.com/watch?v=SZcAJVcbHaY
Heart Sounds revision (~4min): https://www.youtube.com/watch?reload=9&app=desktop&v=dBwr2GZCmQM

## Slide 36
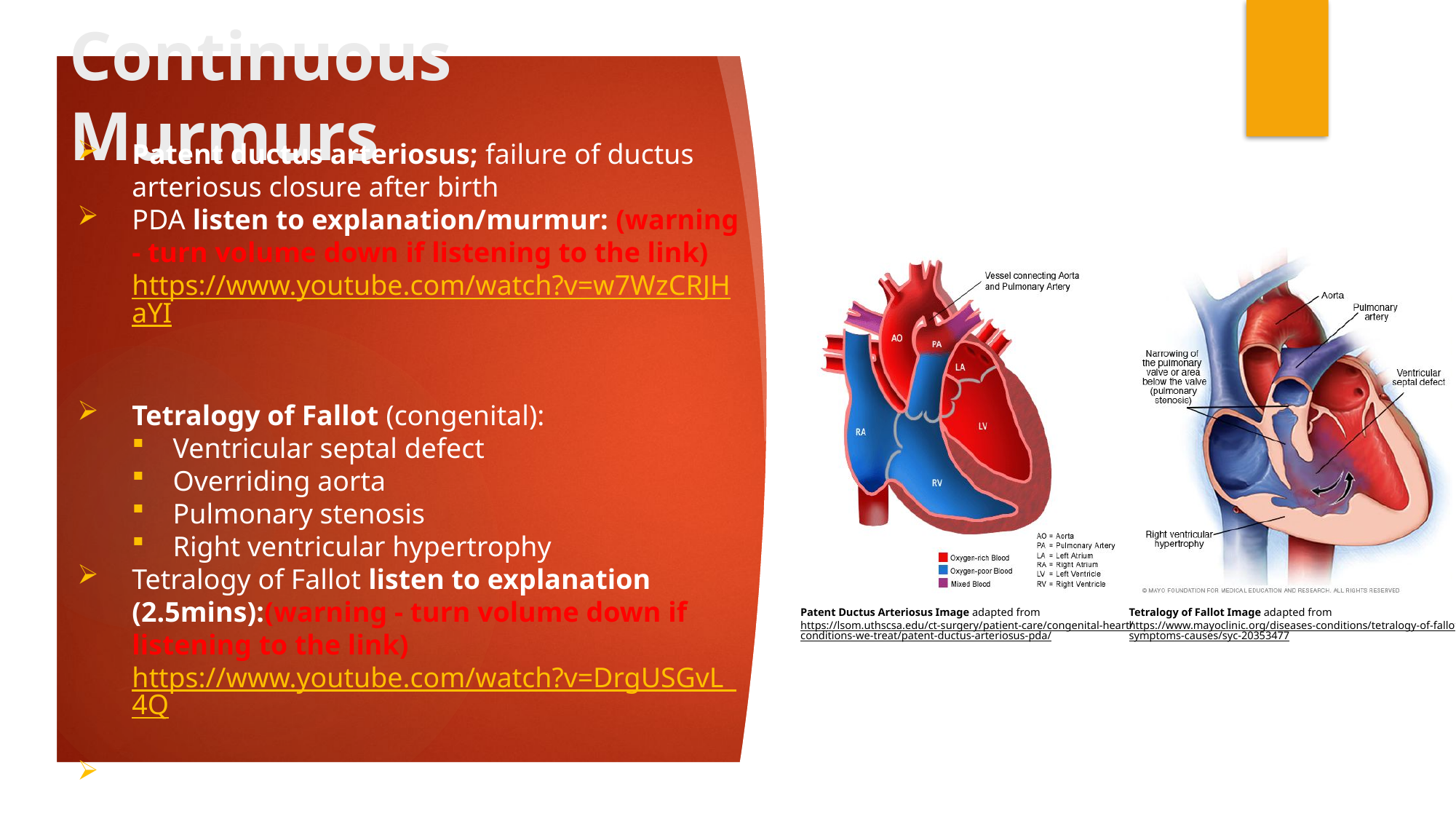

# Continuous Murmurs
Patent ductus arteriosus; failure of ductus arteriosus closure after birth
PDA listen to explanation/murmur: (warning - turn volume down if listening to the link) https://www.youtube.com/watch?v=w7WzCRJHaYI
Tetralogy of Fallot (congenital):
Ventricular septal defect
Overriding aorta
Pulmonary stenosis
Right ventricular hypertrophy
Tetralogy of Fallot listen to explanation (2.5mins):(warning - turn volume down if listening to the link) https://www.youtube.com/watch?v=DrgUSGvL_4Q
Pentalogy of Fallot = Tetralogy of Fallot features + atrial septal defect or PDA
Tetralogy of Fallot Image adapted from https://www.mayoclinic.org/diseases-conditions/tetralogy-of-fallot/symptoms-causes/syc-20353477
Patent Ductus Arteriosus Image adapted from https://lsom.uthscsa.edu/ct-surgery/patient-care/congenital-heart/conditions-we-treat/patent-ductus-arteriosus-pda/

## Slide 37
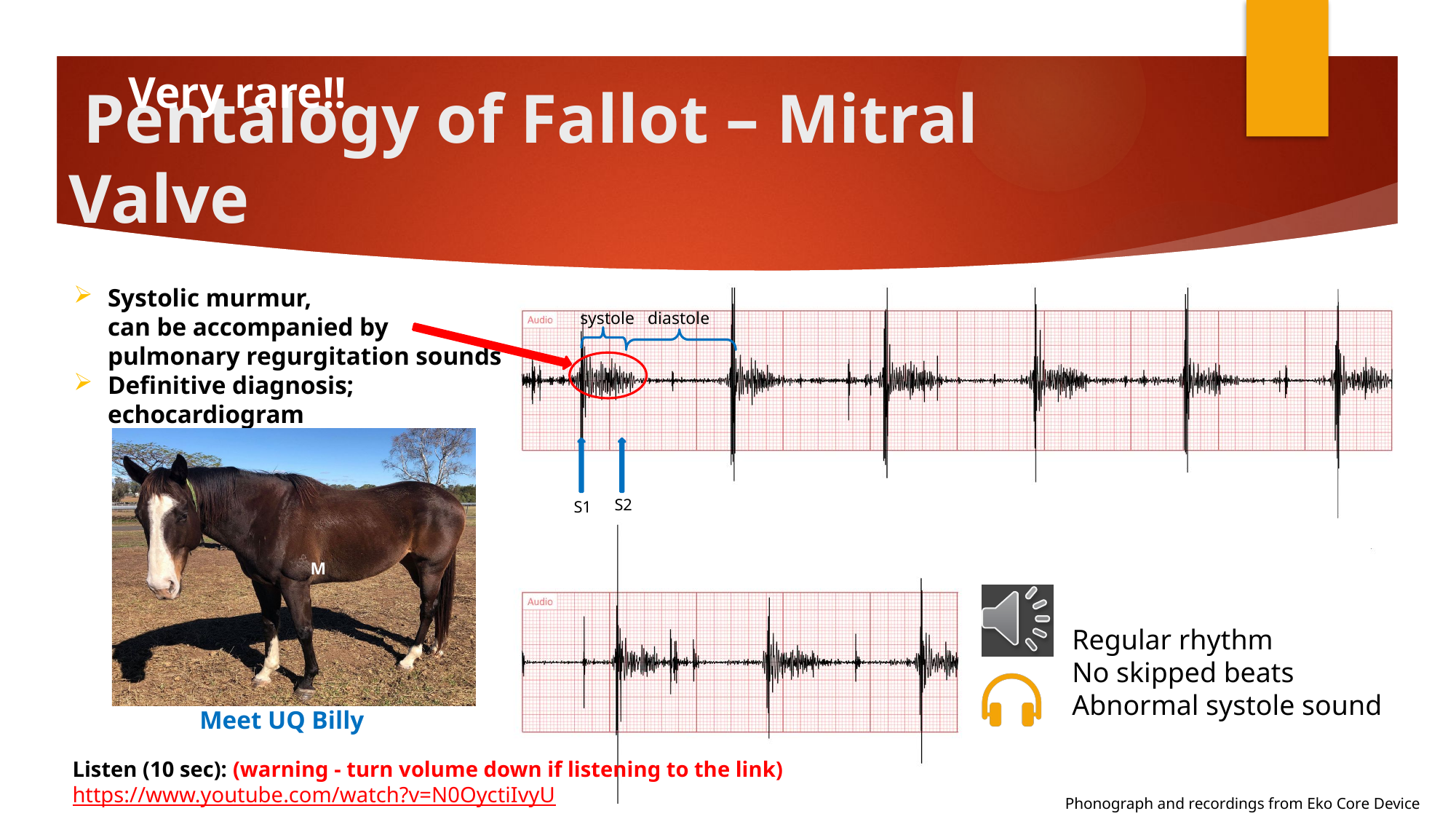

Very rare!!
# Pentalogy of Fallot – Mitral Valve
Systolic murmur,
can be accompanied by pulmonary regurgitation sounds
Definitive diagnosis; echocardiogram
systole
diastole
S2
S1
M
Regular rhythm
No skipped beats
Abnormal systole sound
Meet UQ Billy
Listen (10 sec): (warning - turn volume down if listening to the link)
https://www.youtube.com/watch?v=N0OyctiIvyU
Phonograph and recordings from Eko Core Device

## Slide 38
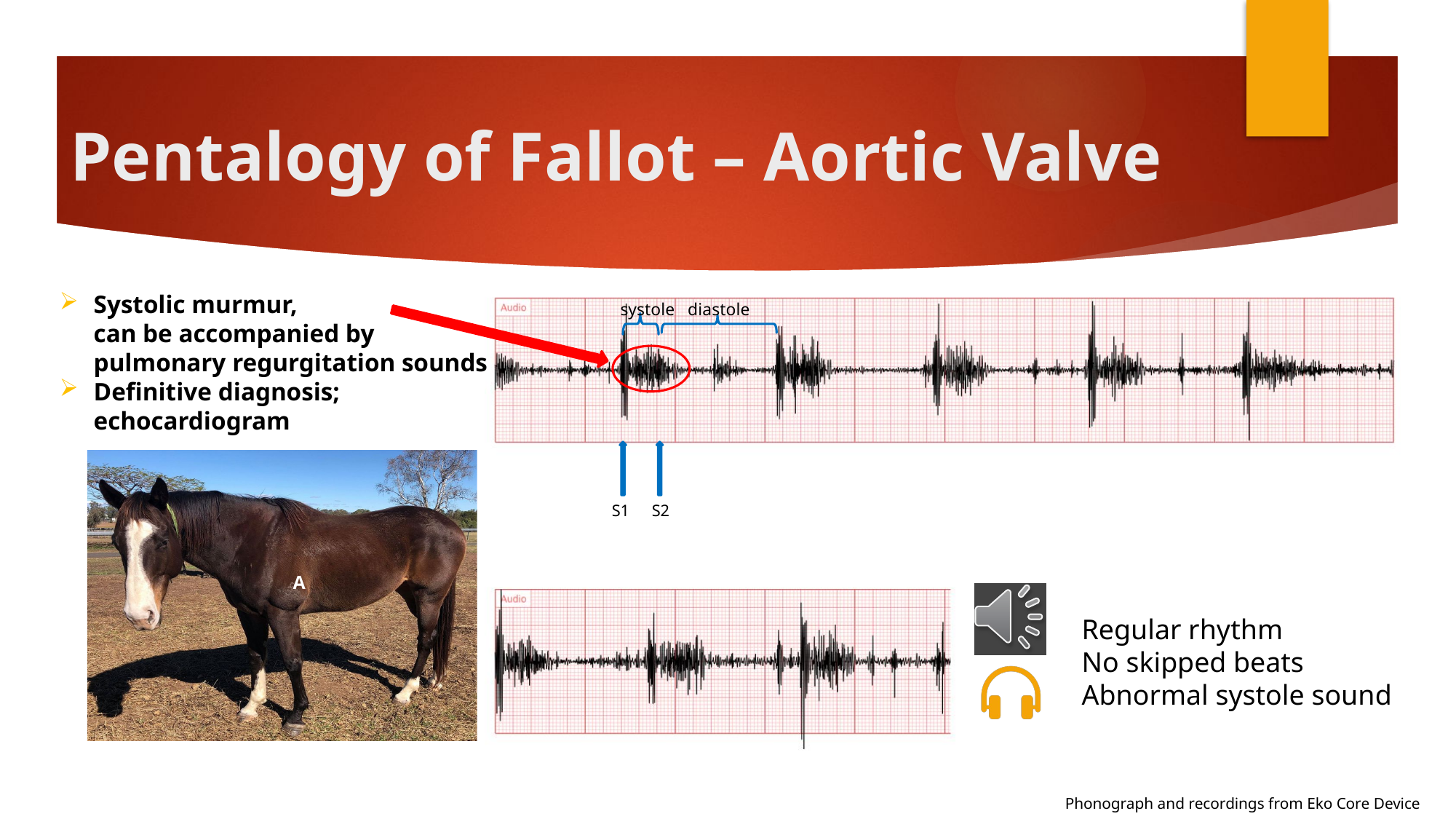

# Pentalogy of Fallot – Aortic Valve
Systolic murmur,
can be accompanied by pulmonary regurgitation sounds
Definitive diagnosis; echocardiogram
systole
diastole
S1
S2
A
Regular rhythm
No skipped beats
Abnormal systole sound
Phonograph and recordings from Eko Core Device

## Slide 39
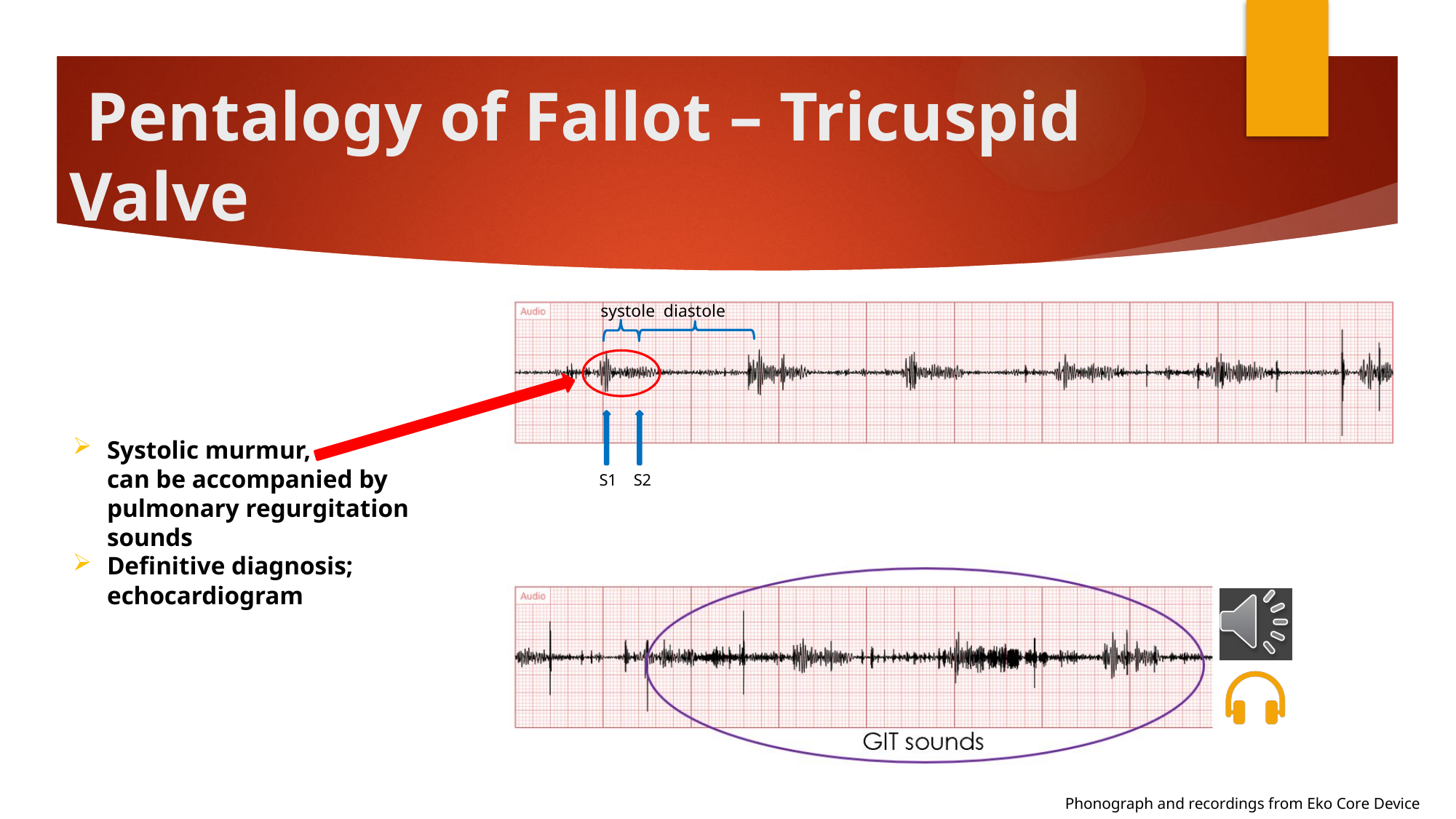

# Pentalogy of Fallot – Tricuspid Valve
systole
diastole
Systolic murmur,
can be accompanied by pulmonary regurgitation sounds
Definitive diagnosis; echocardiogram
S1
S2
Phonograph and recordings from Eko Core Device

## Slide 40
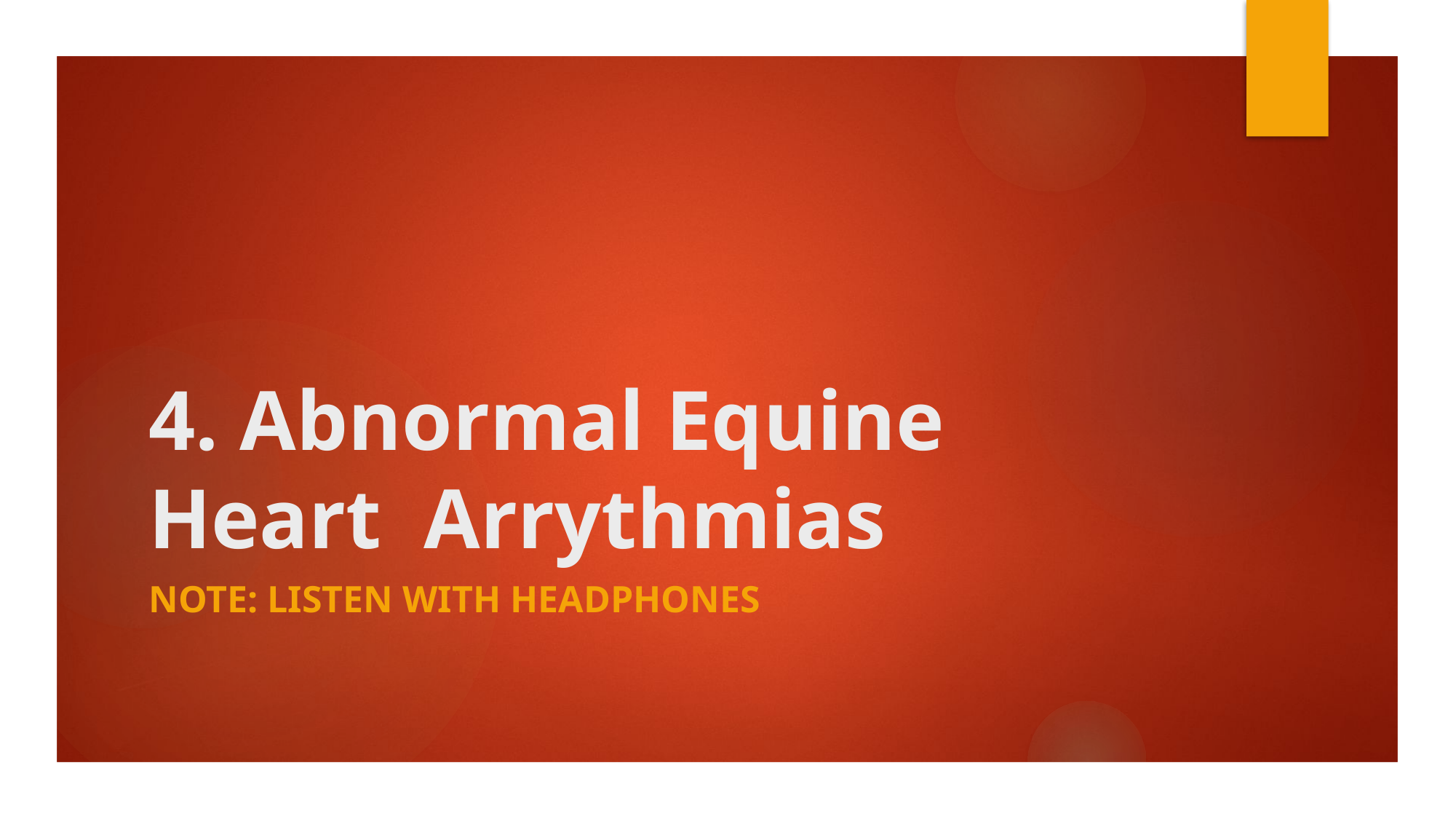

# 4. Abnormal Equine Heart Arrythmias
Note: Listen with HeadPhones

## Slide 41
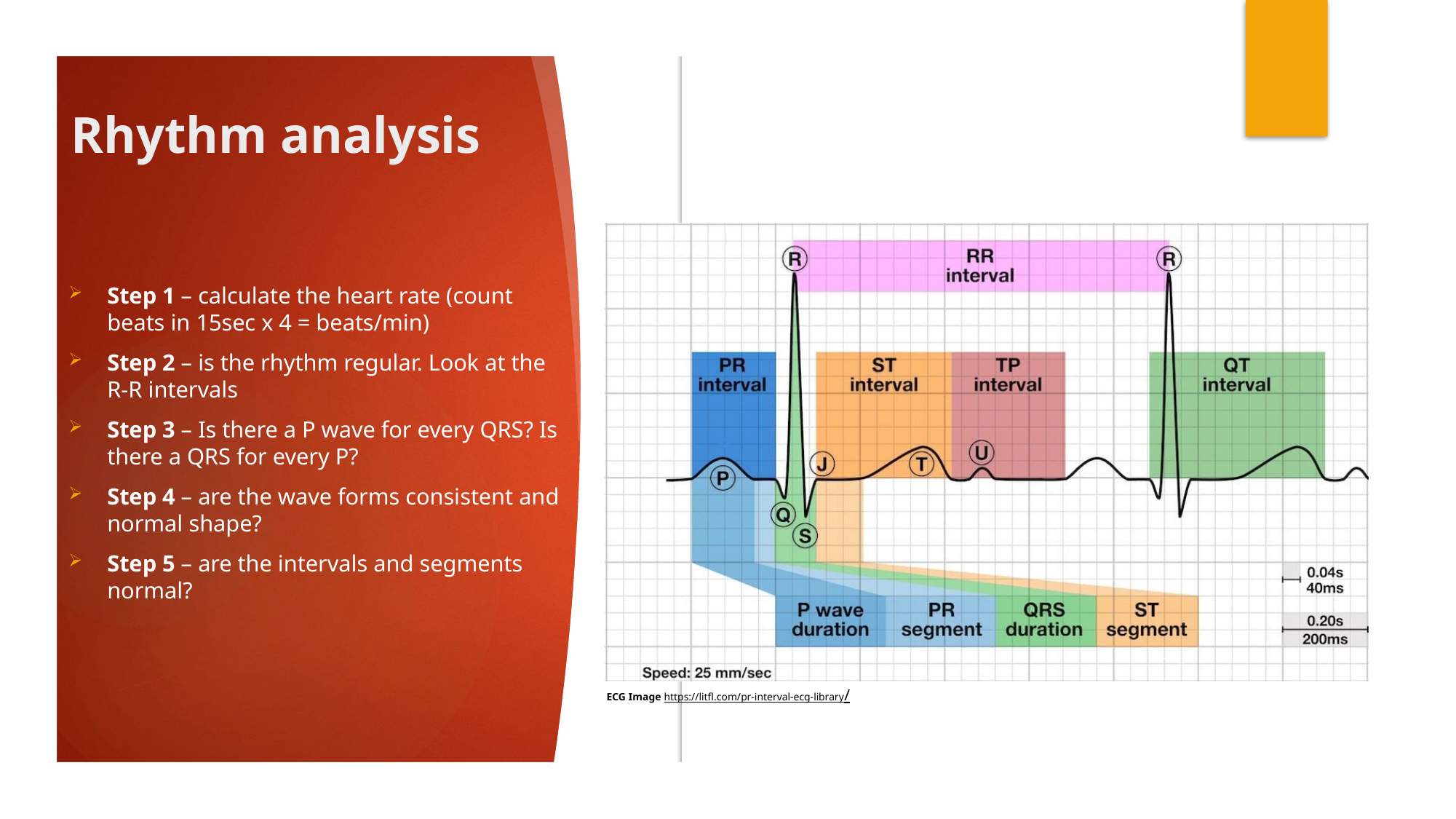

# Rhythm analysis
Step 1 – calculate the heart rate (count beats in 15sec x 4 = beats/min)
Step 2 – is the rhythm regular. Look at the R-R intervals
Step 3 – Is there a P wave for every QRS? Is there a QRS for every P?
Step 4 – are the wave forms consistent and normal shape?
Step 5 – are the intervals and segments normal?
ECG Image https://litfl.com/pr-interval-ecg-library/

## Slide 42
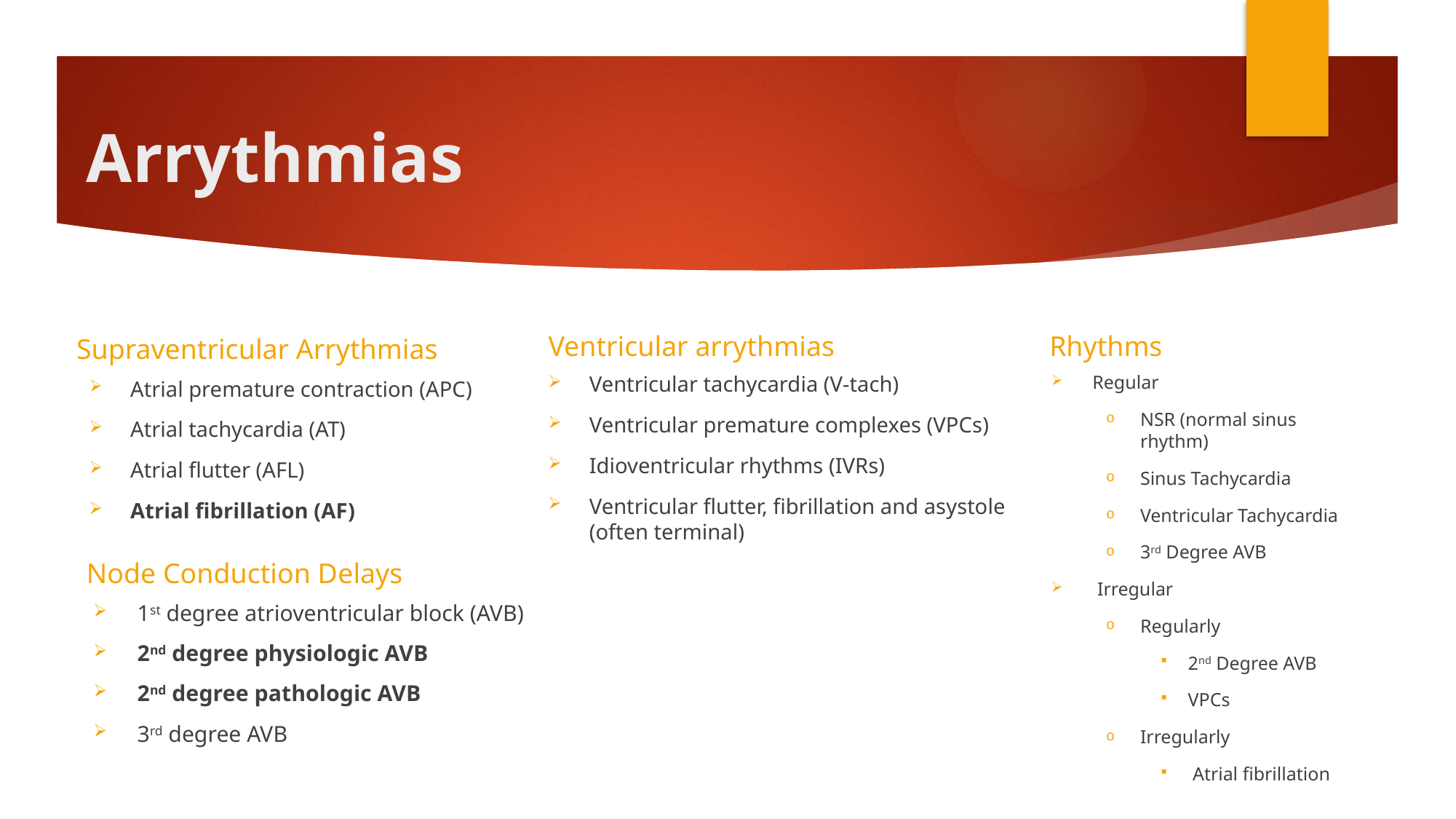

# Arrythmias
Ventricular arrythmias
Rhythms
Supraventricular Arrythmias
Ventricular tachycardia (V-tach)
Ventricular premature complexes (VPCs)
Idioventricular rhythms (IVRs)
Ventricular flutter, fibrillation and asystole (often terminal)
Regular
NSR (normal sinus rhythm)
Sinus Tachycardia
Ventricular Tachycardia
3rd Degree AVB
 Irregular
Regularly
2nd Degree AVB
VPCs
Irregularly
 Atrial fibrillation
Atrial premature contraction (APC)
Atrial tachycardia (AT)
Atrial flutter (AFL)
Atrial fibrillation (AF)
Node Conduction Delays
1st degree atrioventricular block (AVB)
2nd degree physiologic AVB
2nd degree pathologic AVB
3rd degree AVB

## Slide 43
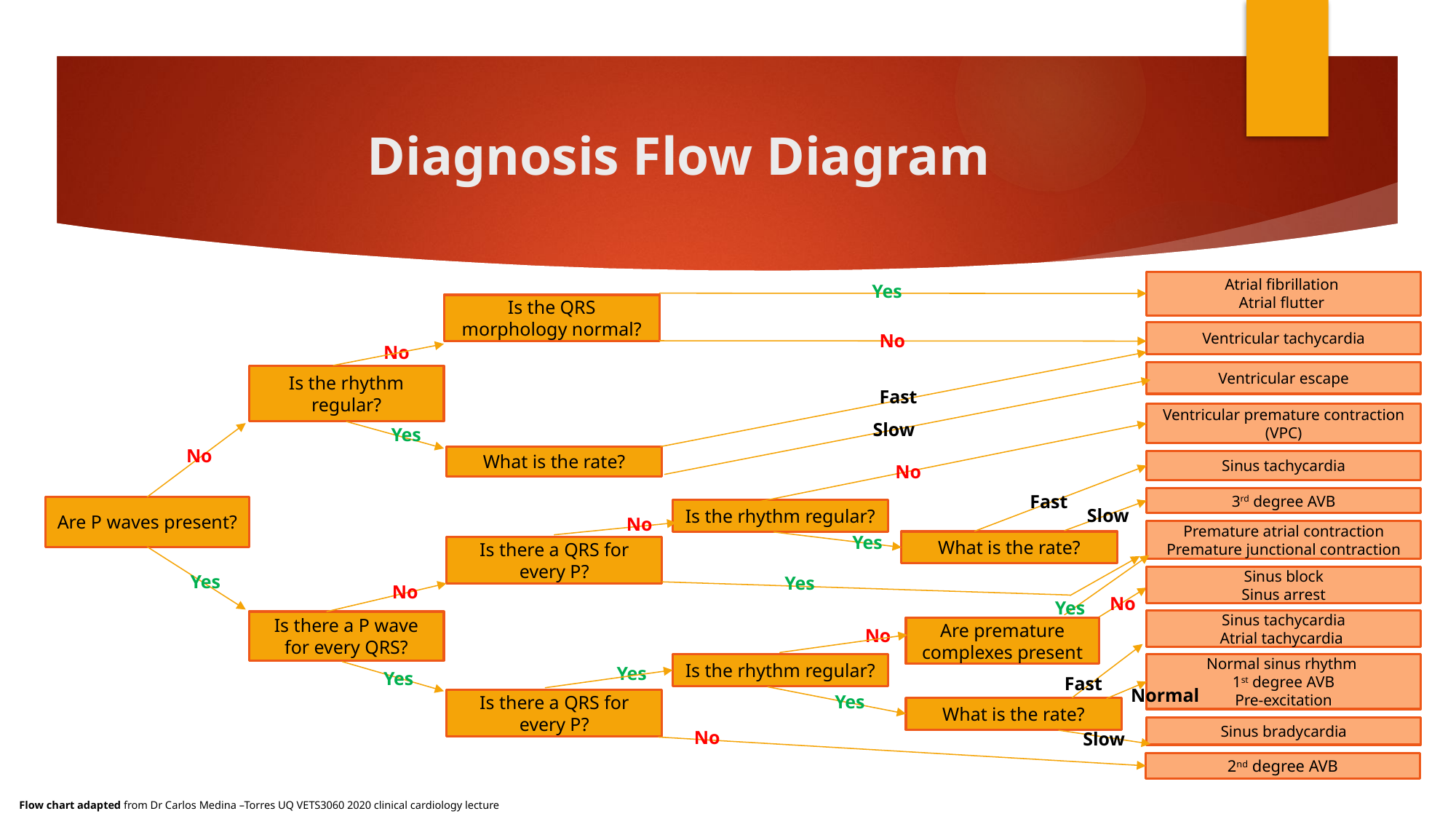

# Diagnosis Flow Diagram
Atrial fibrillation
Atrial flutter
Yes
Is the QRS morphology normal?
Ventricular tachycardia
No
No
Ventricular escape
Is the rhythm regular?
Fast
Ventricular premature contraction (VPC)
Slow
Yes
No
What is the rate?
Sinus tachycardia
No
Fast
3rd degree AVB
Are P waves present?
Slow
Is the rhythm regular?
No
Premature atrial contraction
Premature junctional contraction
Yes
What is the rate?
Is there a QRS for every P?
Yes
Yes
Sinus block
Sinus arrest
No
No
Yes
Sinus tachycardia
Atrial tachycardia
Is there a P wave for every QRS?
Are premature complexes present
No
Is the rhythm regular?
Normal sinus rhythm
1st degree AVB
Pre-excitation
Yes
Yes
Fast
Normal
Yes
Is there a QRS for every P?
What is the rate?
Sinus bradycardia
No
Slow
2nd degree AVB
Flow chart adapted from Dr Carlos Medina –Torres UQ VETS3060 2020 clinical cardiology lecture

## Slide 44
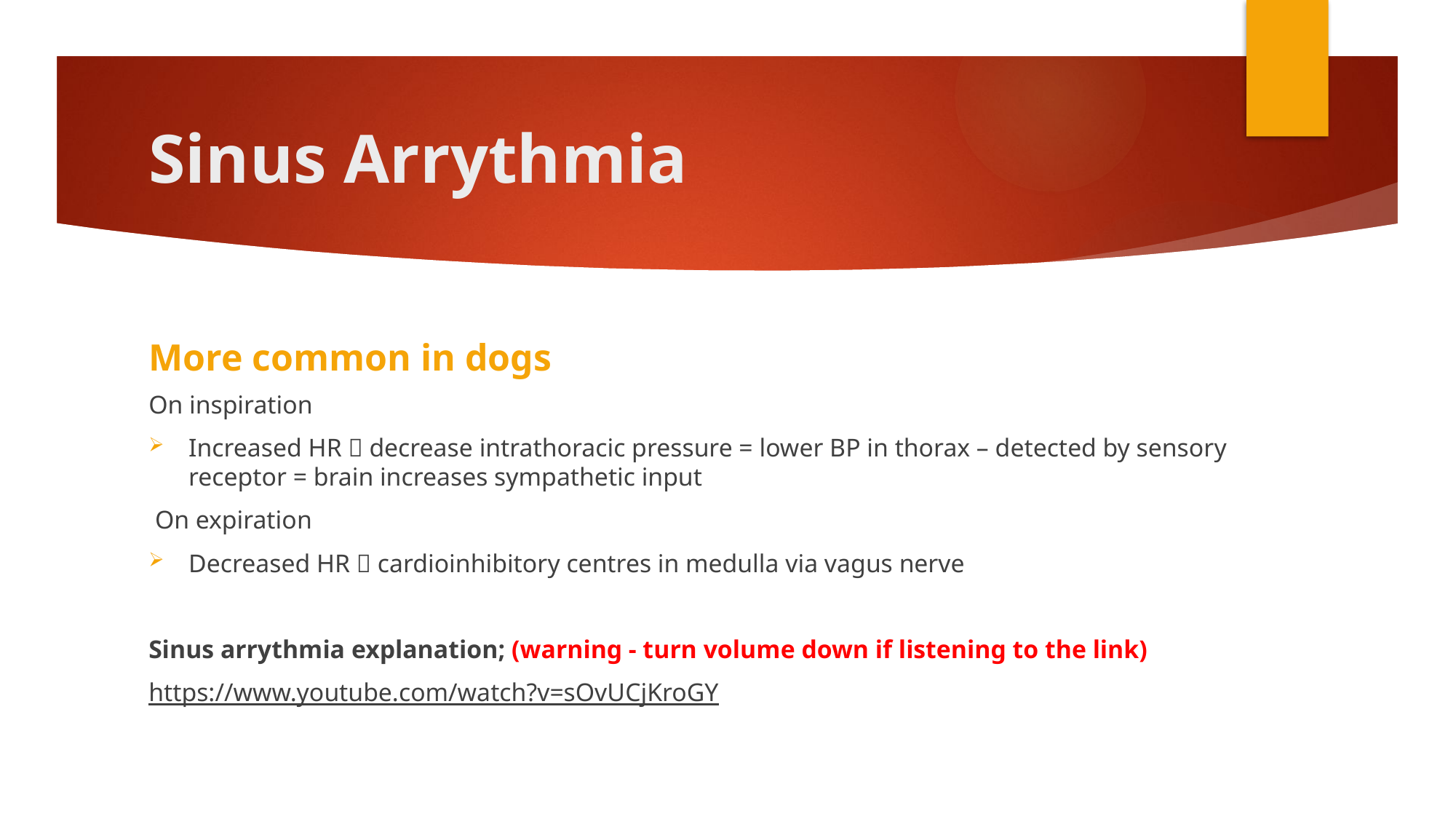

# Sinus Arrythmia
More common in dogs
On inspiration
Increased HR  decrease intrathoracic pressure = lower BP in thorax – detected by sensory receptor = brain increases sympathetic input
 On expiration
Decreased HR  cardioinhibitory centres in medulla via vagus nerve
Sinus arrythmia explanation; (warning - turn volume down if listening to the link)
https://www.youtube.com/watch?v=sOvUCjKroGY

## Slide 45
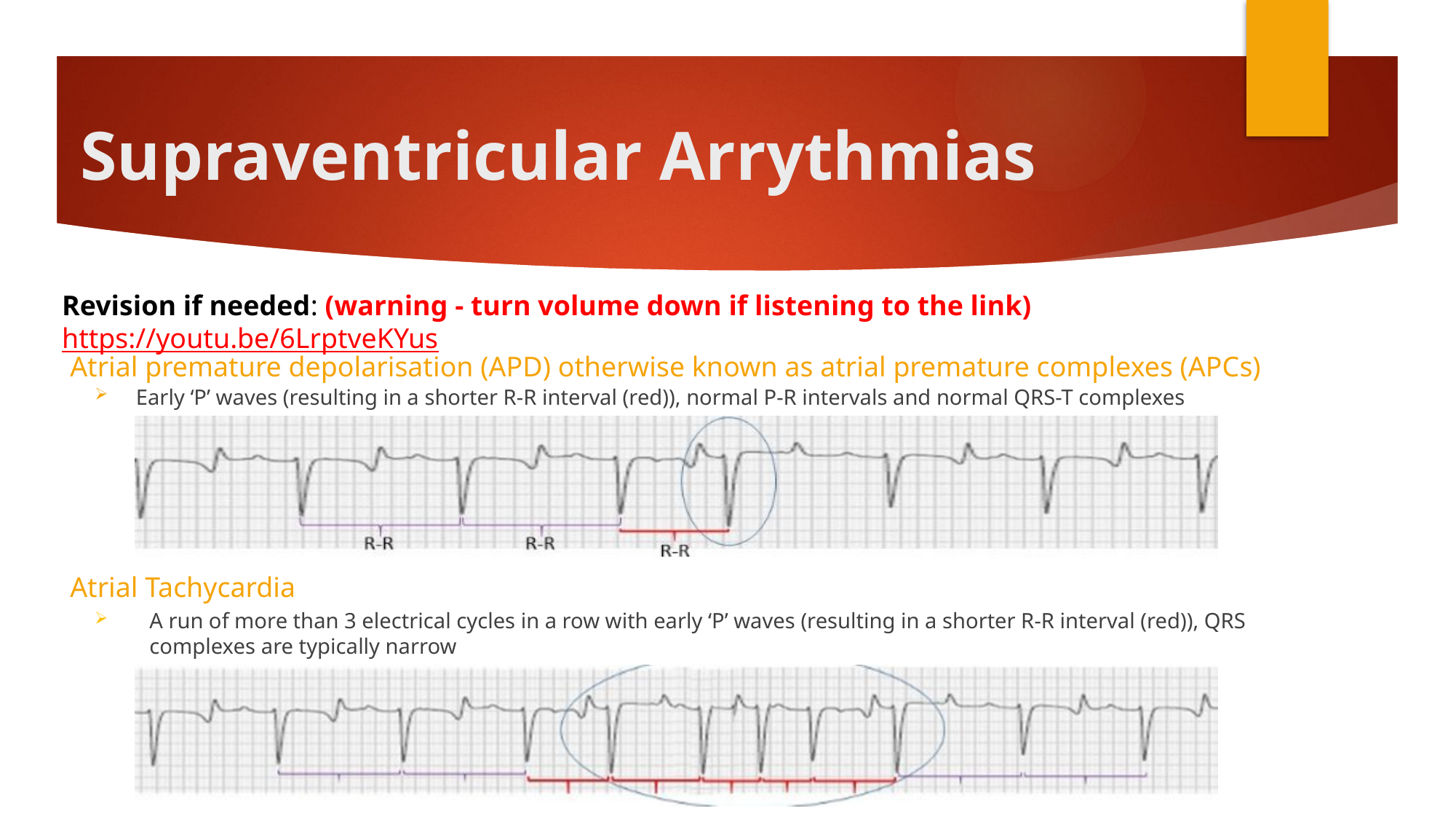

# Supraventricular Arrythmias
Revision if needed: (warning - turn volume down if listening to the link) https://youtu.be/6LrptveKYus
Atrial premature depolarisation (APD) otherwise known as atrial premature complexes (APCs)
Early ‘P’ waves (resulting in a shorter R-R interval (red)), normal P-R intervals and normal QRS-T complexes
Atrial Tachycardia
A run of more than 3 electrical cycles in a row with early ‘P’ waves (resulting in a shorter R-R interval (red)), QRS complexes are typically narrow

## Slide 46
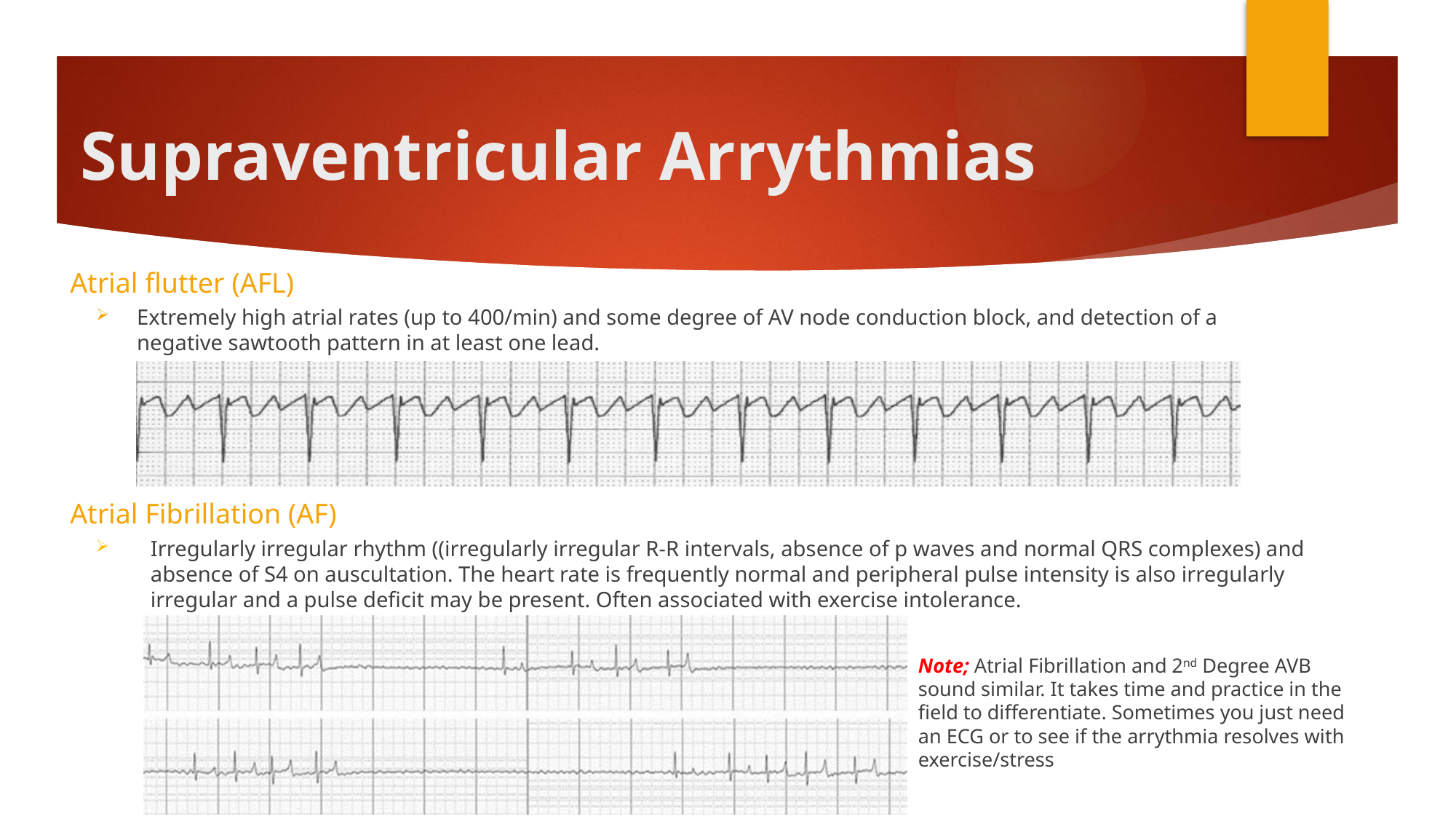

# Supraventricular Arrythmias
Atrial flutter (AFL)
Extremely high atrial rates (up to 400/min) and some degree of AV node conduction block, and detection of a negative sawtooth pattern in at least one lead.
Atrial Fibrillation (AF)
Irregularly irregular rhythm ((irregularly irregular R-R intervals, absence of p waves and normal QRS complexes) and absence of S4 on auscultation. The heart rate is frequently normal and peripheral pulse intensity is also irregularly irregular and a pulse deficit may be present. Often associated with exercise intolerance.
Note; Atrial Fibrillation and 2nd Degree AVB sound similar. It takes time and practice in the field to differentiate. Sometimes you just need an ECG or to see if the arrythmia resolves with exercise/stress

## Slide 47
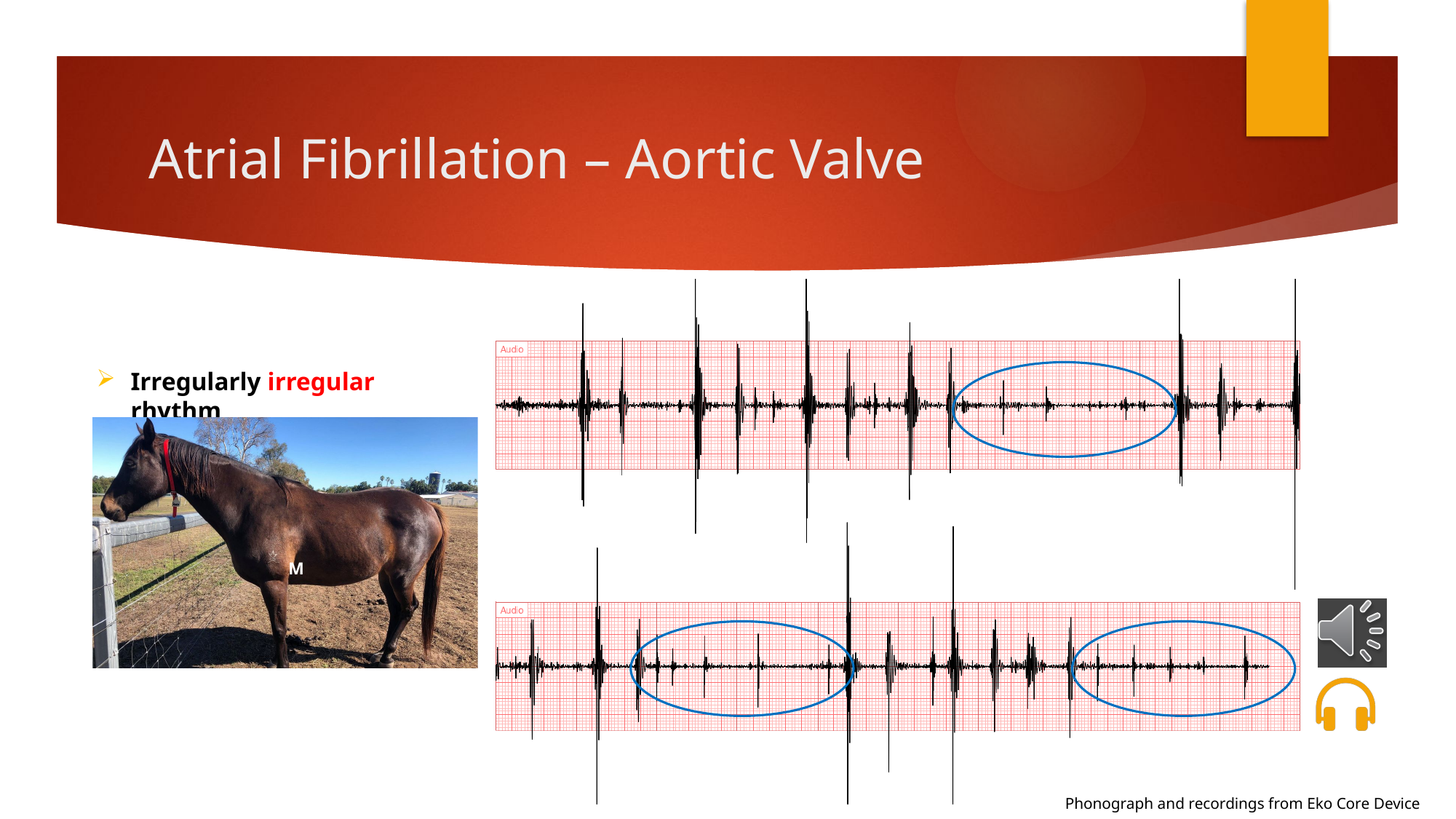

# Atrial Fibrillation – Aortic Valve
Irregularly irregular rhythm
M
Phonograph and recordings from Eko Core Device

## Slide 48
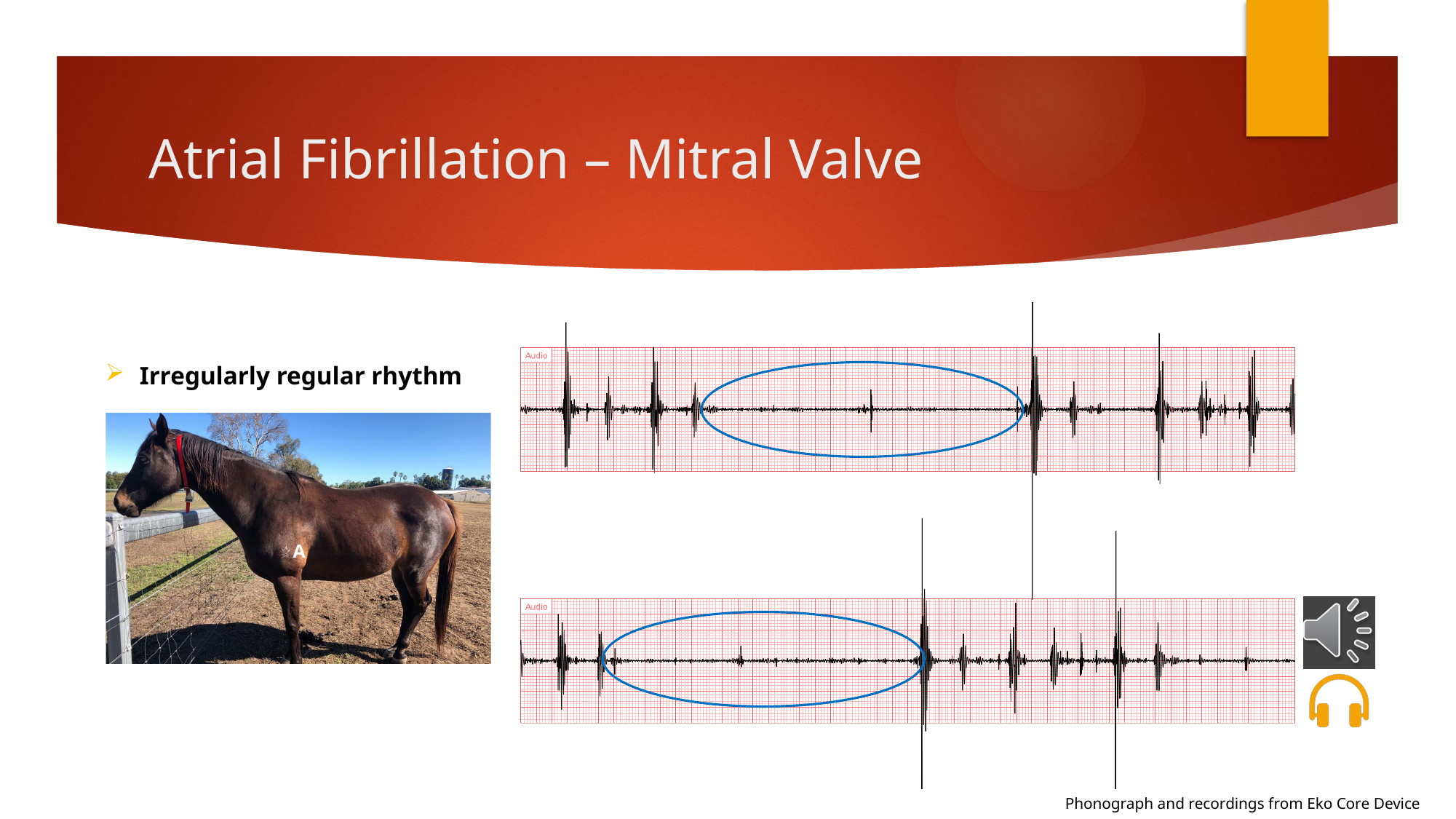

# Atrial Fibrillation – Mitral Valve
Irregularly regular rhythm
A
Phonograph and recordings from Eko Core Device

## Slide 49
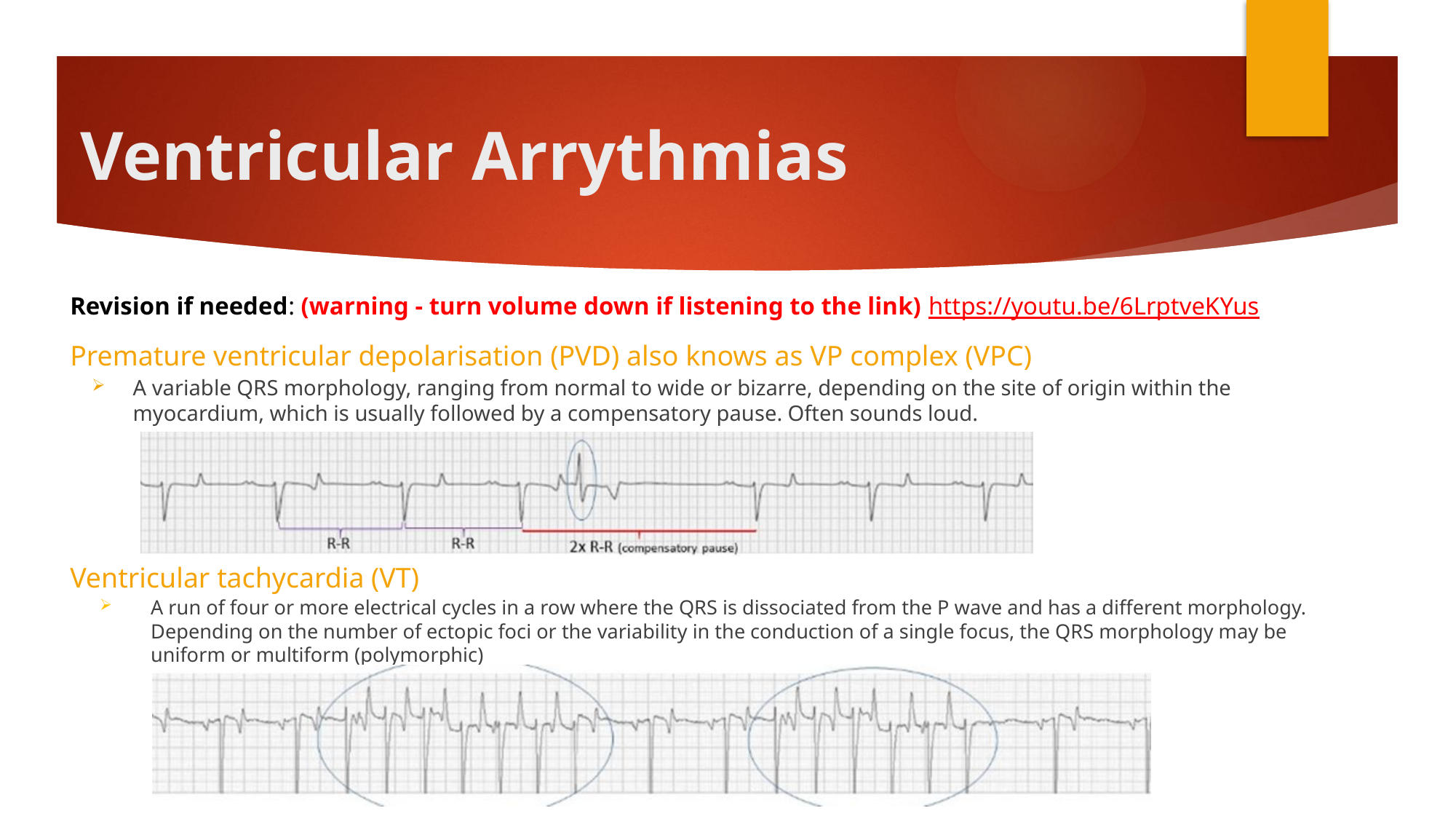

# Ventricular Arrythmias
Revision if needed: (warning - turn volume down if listening to the link) https://youtu.be/6LrptveKYus
Premature ventricular depolarisation (PVD) also knows as VP complex (VPC)
A variable QRS morphology, ranging from normal to wide or bizarre, depending on the site of origin within the myocardium, which is usually followed by a compensatory pause. Often sounds loud.
Ventricular tachycardia (VT)
A run of four or more electrical cycles in a row where the QRS is dissociated from the P wave and has a different morphology. Depending on the number of ectopic foci or the variability in the conduction of a single focus, the QRS morphology may be uniform or multiform (polymorphic)

## Slide 50
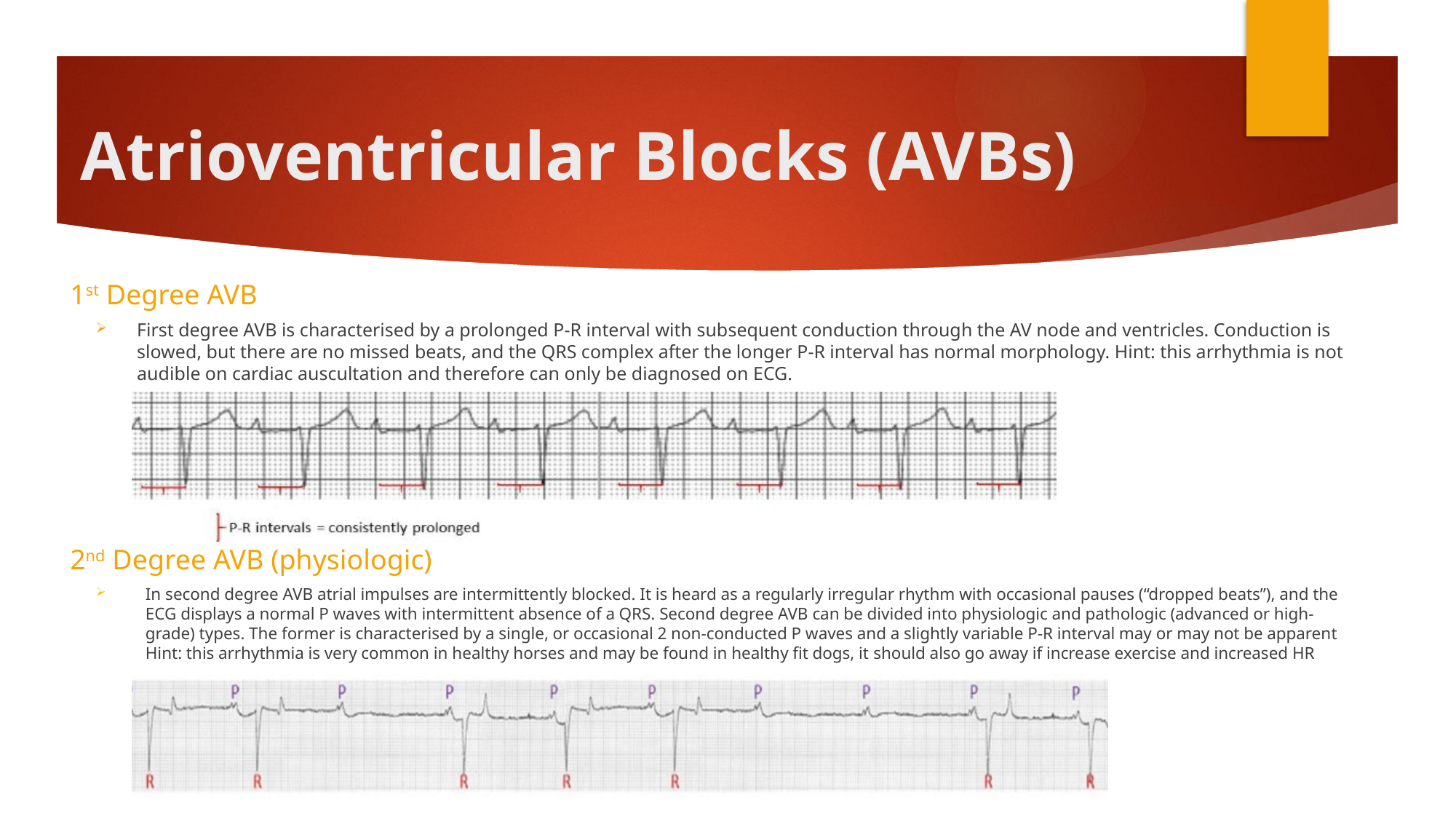

# Atrioventricular Blocks (AVBs)
1st Degree AVB
First degree AVB is characterised by a prolonged P-R interval with subsequent conduction through the AV node and ventricles. Conduction is slowed, but there are no missed beats, and the QRS complex after the longer P-R interval has normal morphology. Hint: this arrhythmia is not audible on cardiac auscultation and therefore can only be diagnosed on ECG.
2nd Degree AVB (physiologic)
In second degree AVB atrial impulses are intermittently blocked. It is heard as a regularly irregular rhythm with occasional pauses (“dropped beats”), and the ECG displays a normal P waves with intermittent absence of a QRS. Second degree AVB can be divided into physiologic and pathologic (advanced or high-grade) types. The former is characterised by a single, or occasional 2 non-conducted P waves and a slightly variable P-R interval may or may not be apparent Hint: this arrhythmia is very common in healthy horses and may be found in healthy fit dogs, it should also go away if increase exercise and increased HR

## Slide 51
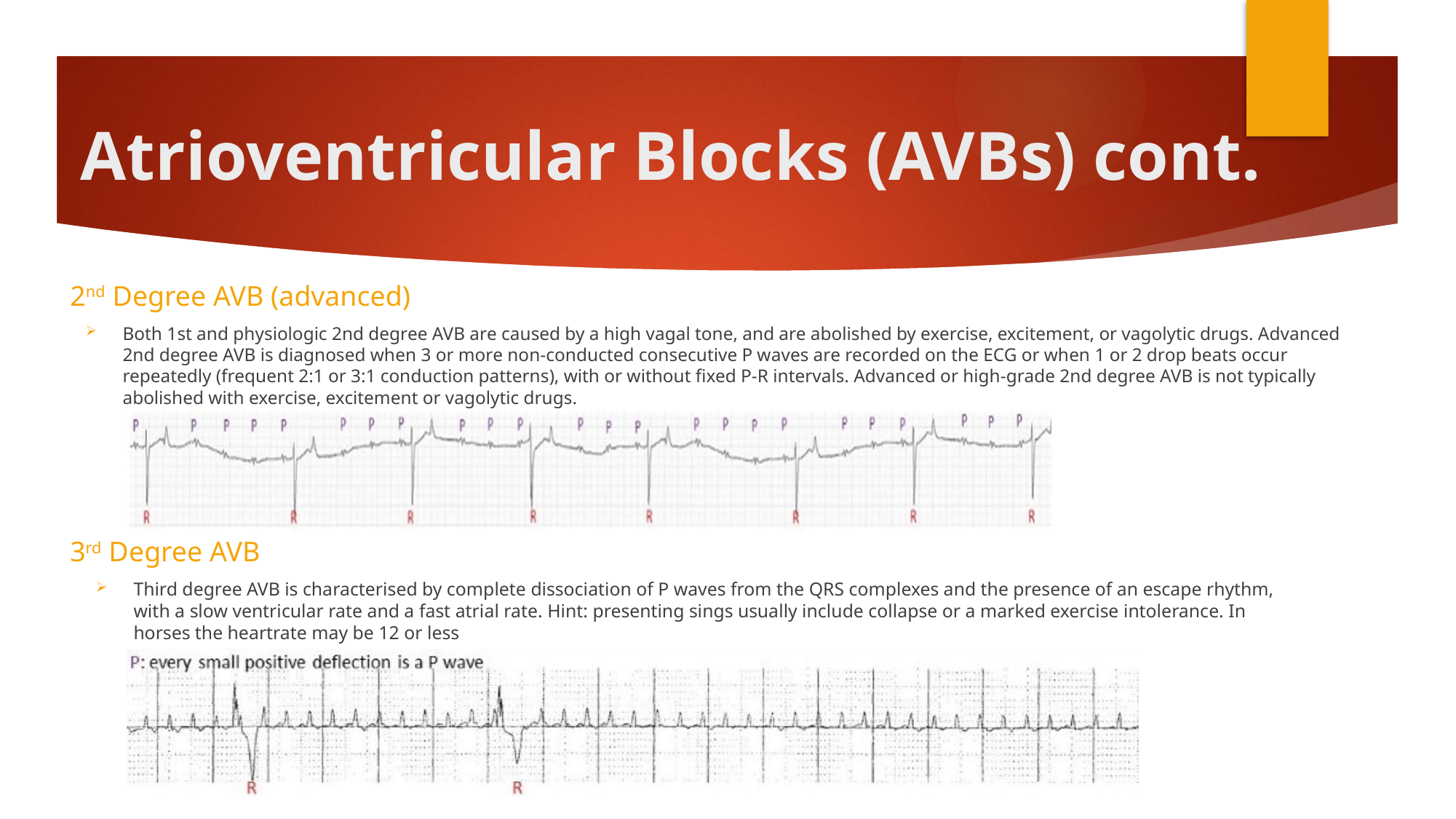

# Atrioventricular Blocks (AVBs) cont.
2nd Degree AVB (advanced)
Both 1st and physiologic 2nd degree AVB are caused by a high vagal tone, and are abolished by exercise, excitement, or vagolytic drugs. Advanced 2nd degree AVB is diagnosed when 3 or more non-conducted consecutive P waves are recorded on the ECG or when 1 or 2 drop beats occur repeatedly (frequent 2:1 or 3:1 conduction patterns), with or without fixed P-R intervals. Advanced or high-grade 2nd degree AVB is not typically abolished with exercise, excitement or vagolytic drugs.
3rd Degree AVB
Third degree AVB is characterised by complete dissociation of P waves from the QRS complexes and the presence of an escape rhythm, with a slow ventricular rate and a fast atrial rate. Hint: presenting sings usually include collapse or a marked exercise intolerance. In horses the heartrate may be 12 or less

## Slide 52
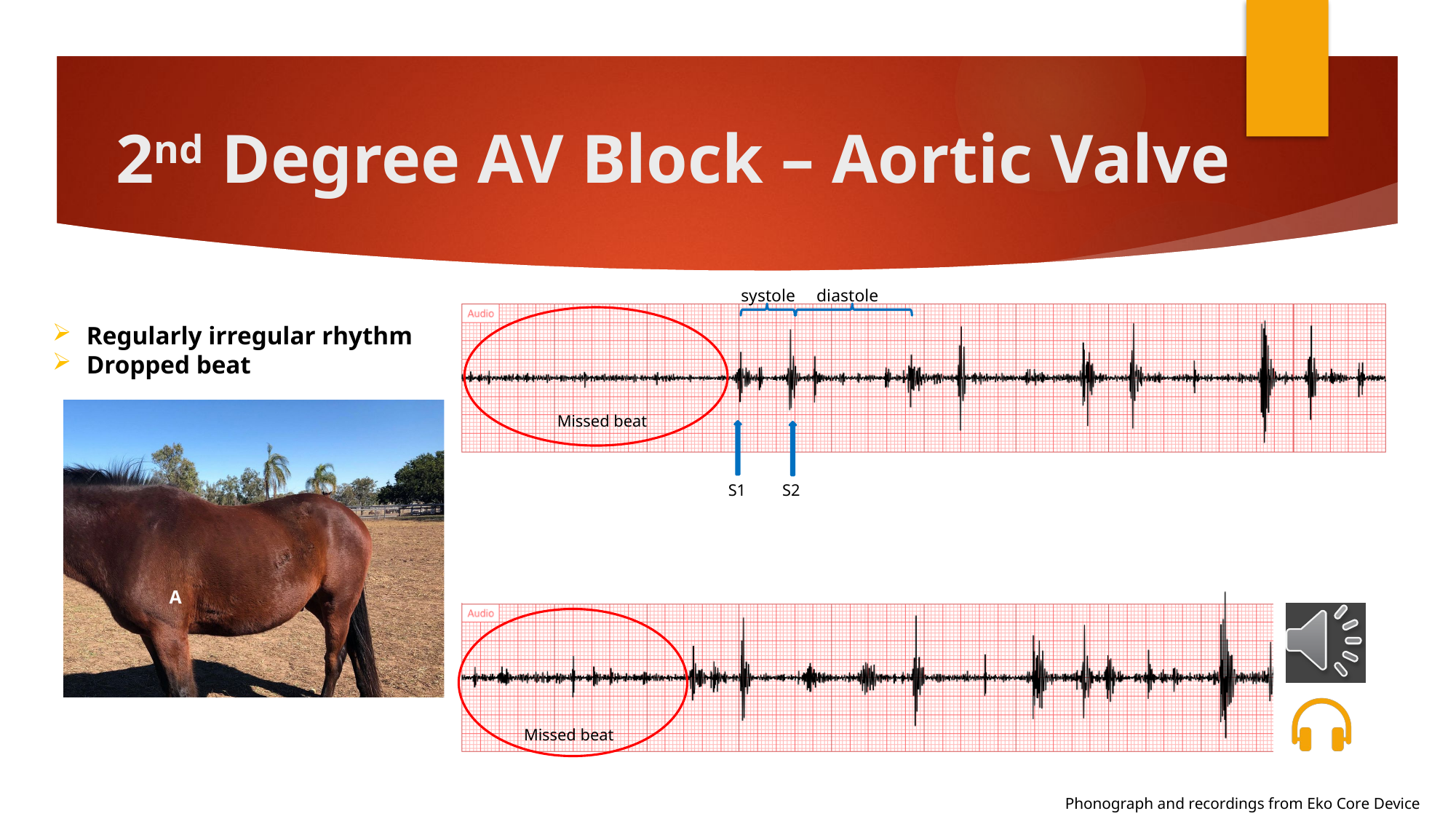

# 2nd Degree AV Block – Aortic Valve
systole
diastole
Regularly irregular rhythm
Dropped beat
Missed beat
S1
S2
A
Missed beat
Phonograph and recordings from Eko Core Device

## Slide 53
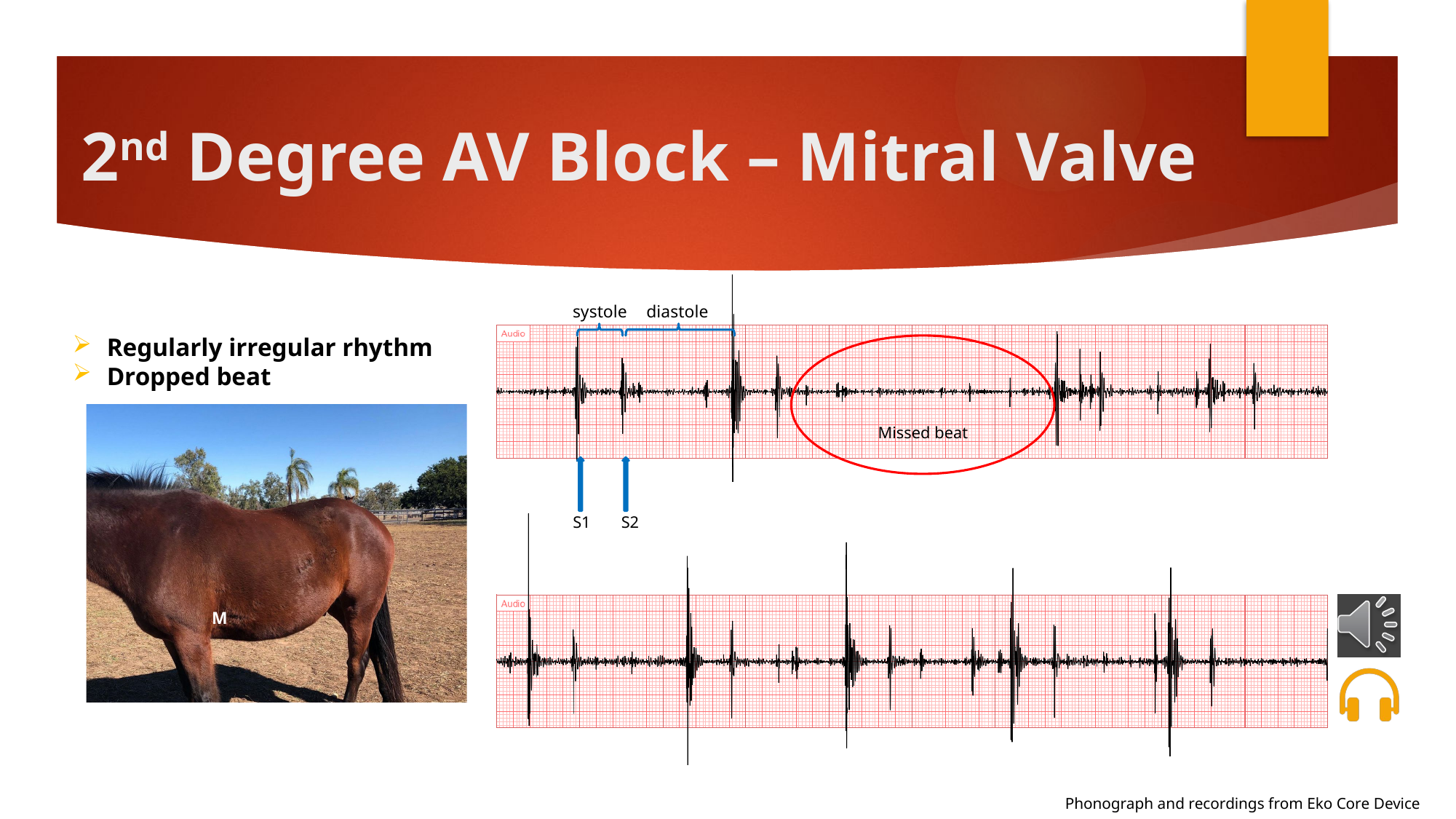

# 2nd Degree AV Block – Mitral Valve
systole
diastole
Regularly irregular rhythm
Dropped beat
Missed beat
S1
S2
M
Phonograph and recordings from Eko Core Device

## Slide 54
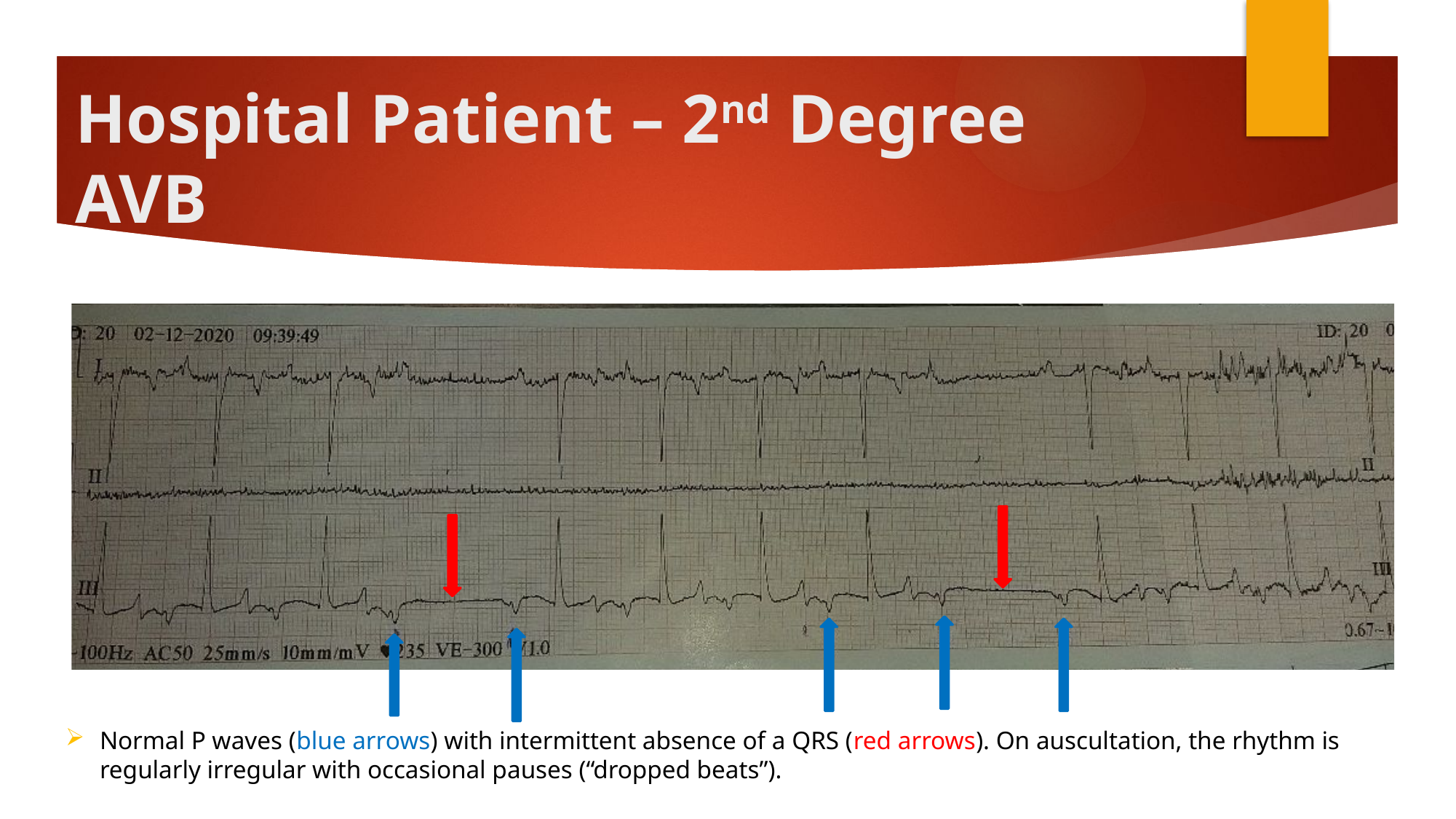

# Hospital Patient – 2nd Degree AVB
Normal P waves (blue arrows) with intermittent absence of a QRS (red arrows). On auscultation, the rhythm is regularly irregular with occasional pauses (“dropped beats”).

## Slide 55
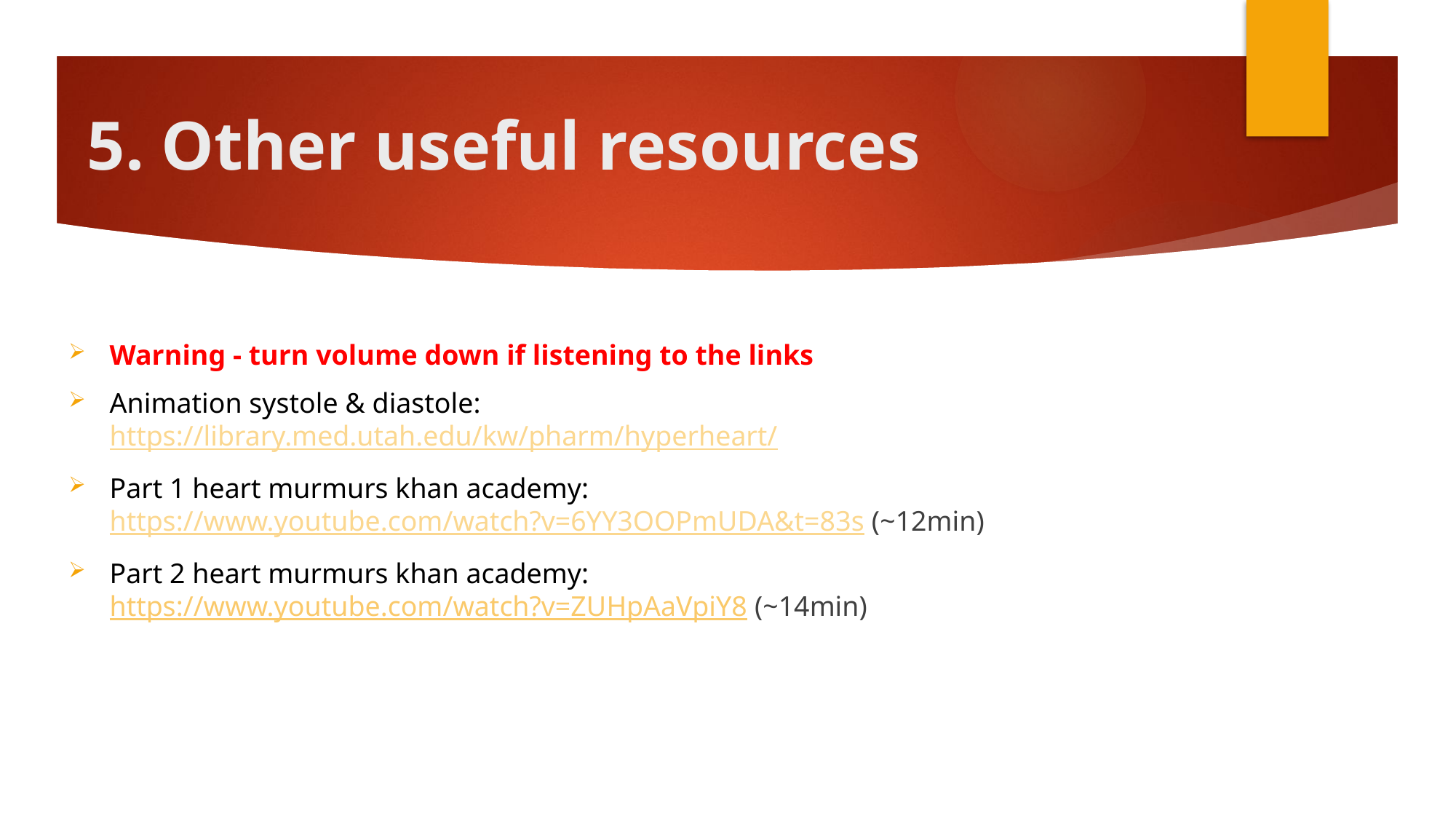

# 5. Other useful resources
Warning - turn volume down if listening to the links
Animation systole & diastole: https://library.med.utah.edu/kw/pharm/hyperheart/
Part 1 heart murmurs khan academy: https://www.youtube.com/watch?v=6YY3OOPmUDA&t=83s (~12min)
Part 2 heart murmurs khan academy: https://www.youtube.com/watch?v=ZUHpAaVpiY8 (~14min)

## Slide 56
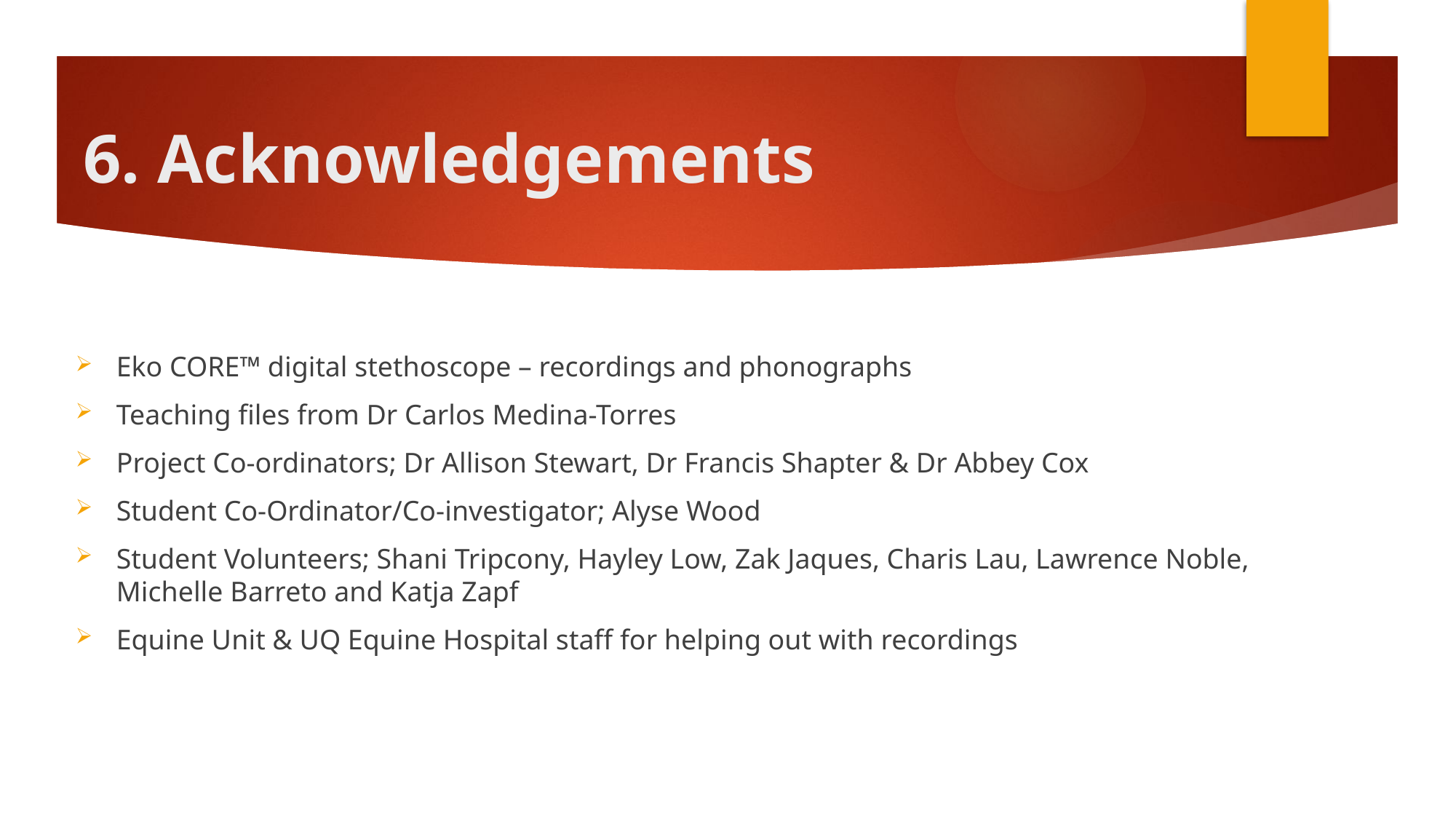

# 6. Acknowledgements
Eko CORE™ digital stethoscope – recordings and phonographs
Teaching files from Dr Carlos Medina-Torres
Project Co-ordinators; Dr Allison Stewart, Dr Francis Shapter & Dr Abbey Cox
Student Co-Ordinator/Co-investigator; Alyse Wood
Student Volunteers; Shani Tripcony, Hayley Low, Zak Jaques, Charis Lau, Lawrence Noble, Michelle Barreto and Katja Zapf
Equine Unit & UQ Equine Hospital staff for helping out with recordings

## Slide 57
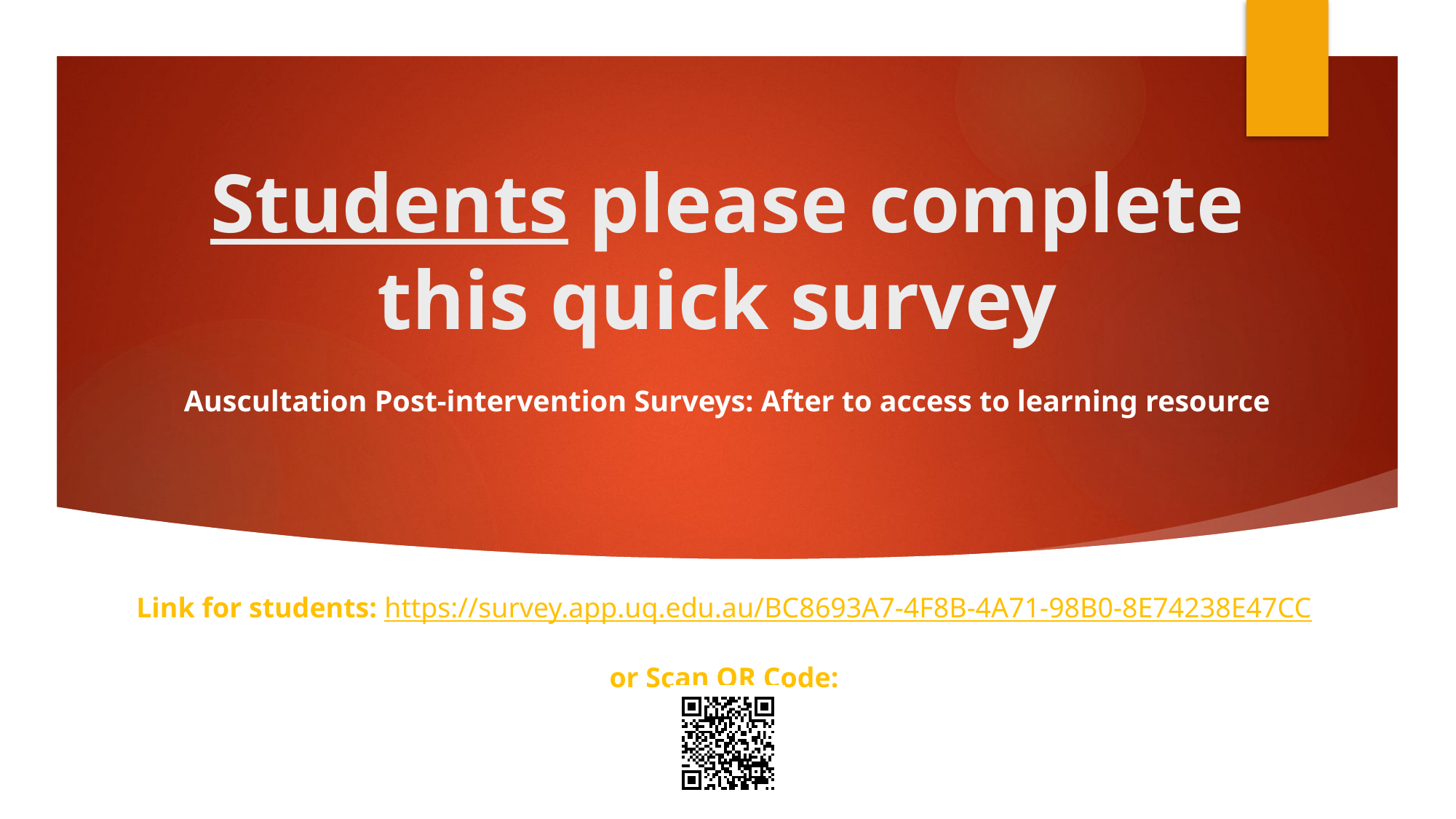

Students please complete this quick survey
Auscultation Post-intervention Surveys: After to access to learning resource
Link for students: https://survey.app.uq.edu.au/BC8693A7-4F8B-4A71-98B0-8E74238E47CC
or Scan QR Code:

## Slide 58
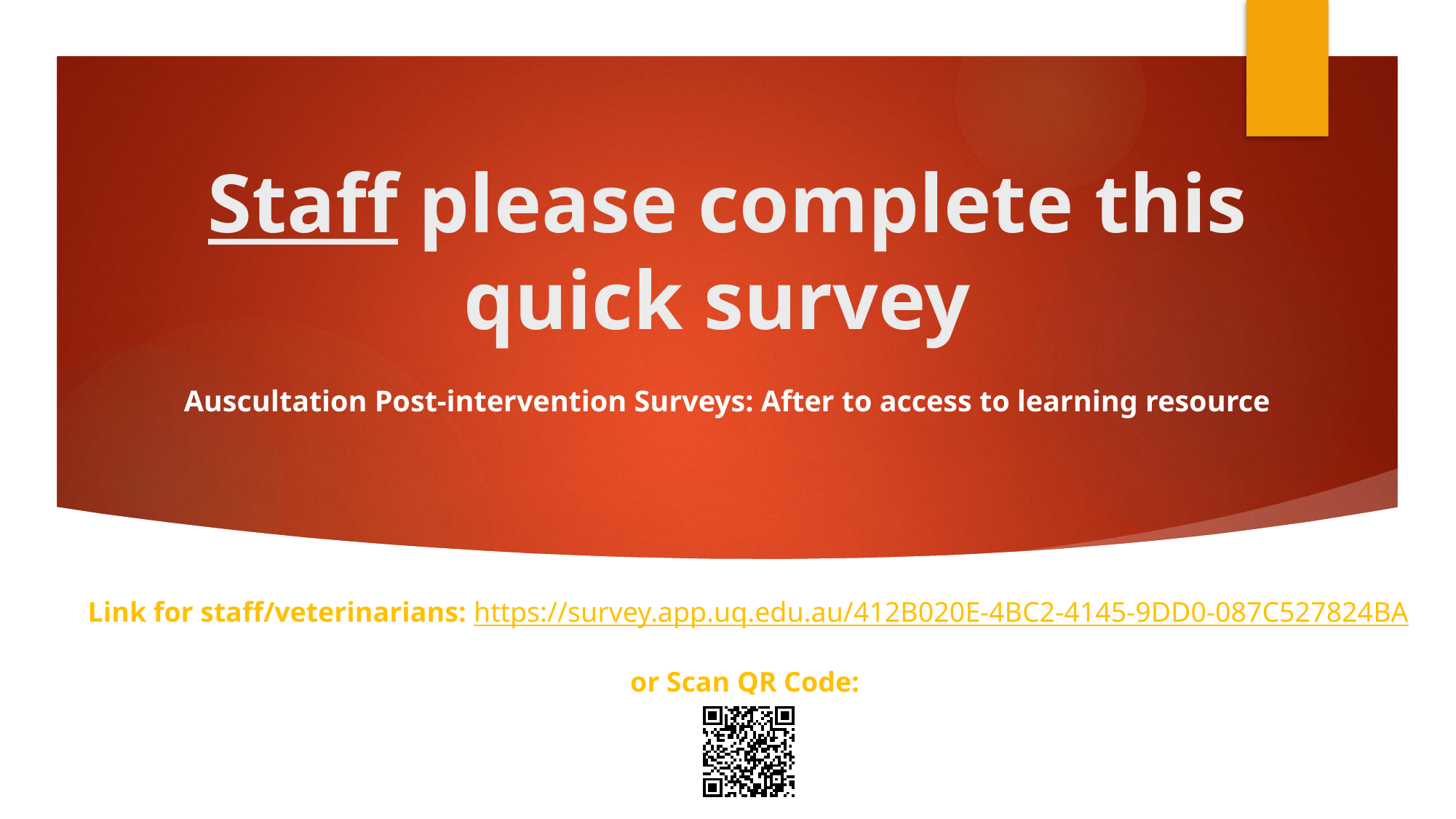

Staff please complete this quick survey
Auscultation Post-intervention Surveys: After to access to learning resource
Link for staff/veterinarians: https://survey.app.uq.edu.au/412B020E-4BC2-4145-9DD0-087C527824BA
or Scan QR Code:

## Slide 59
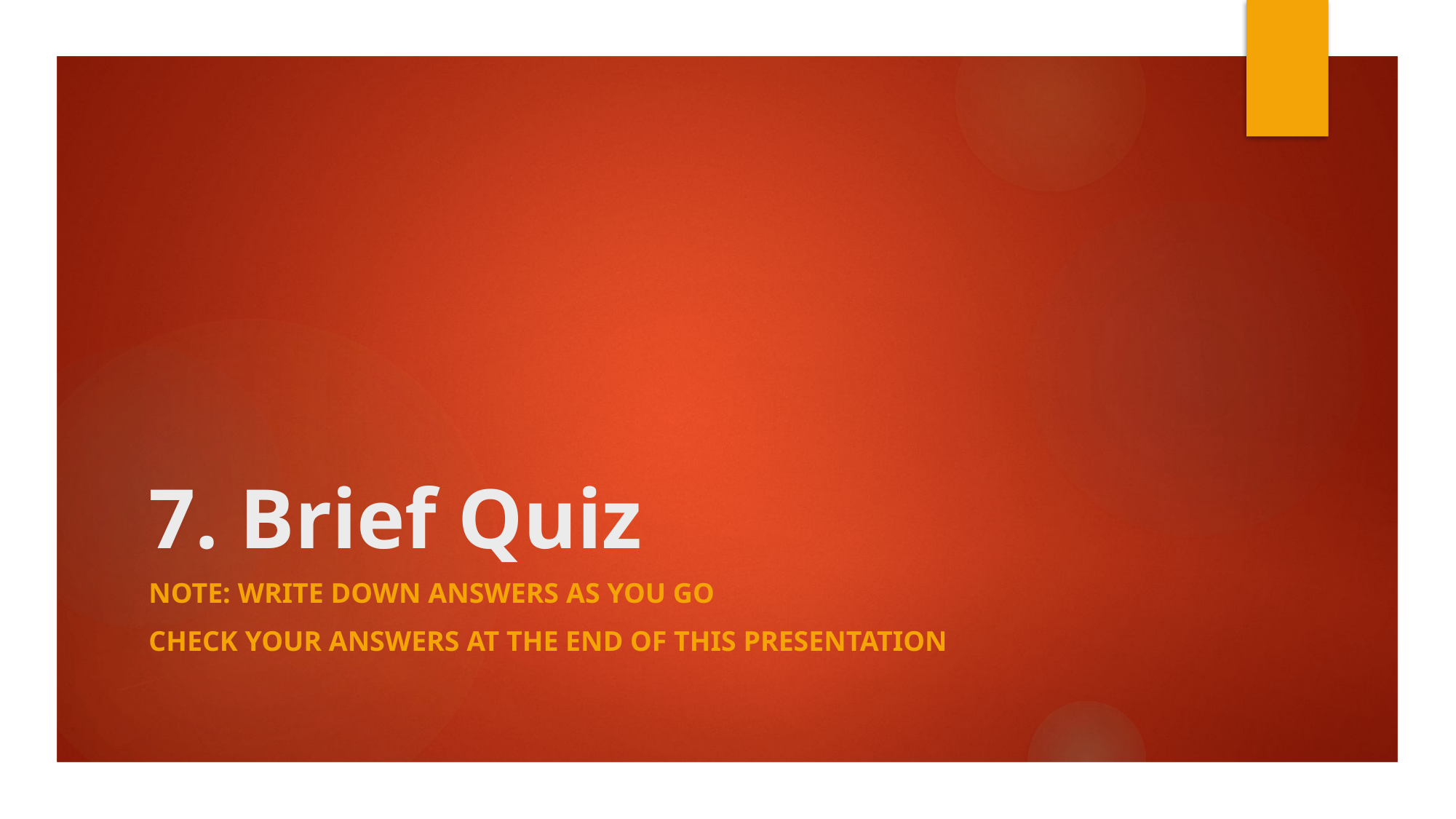

# 7. Brief Quiz
Note: write down answers as you go
Check your answers at the end of this Presentation

## Slide 60
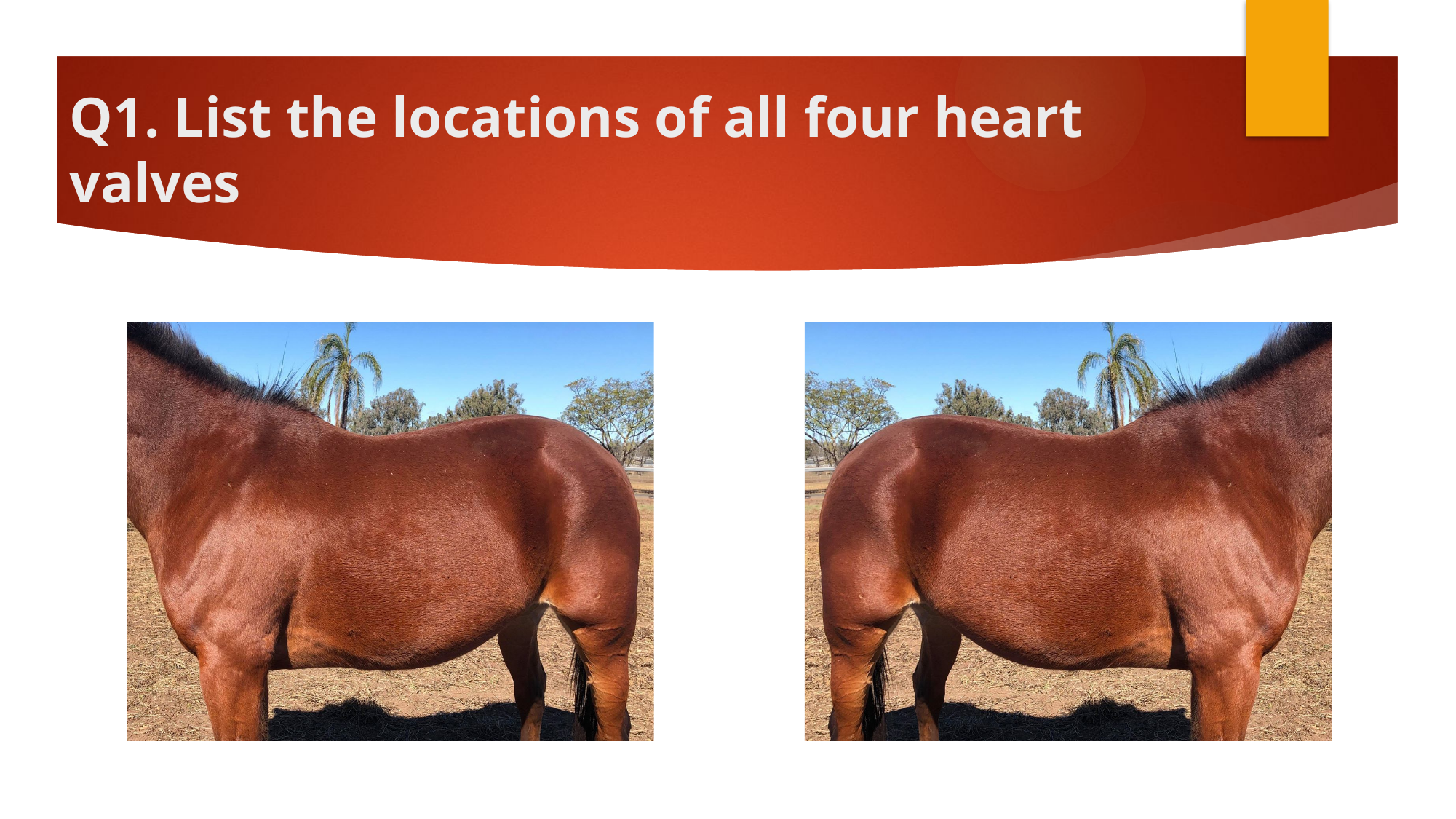

Q1. List the locations of all four heart valves

## Slide 61
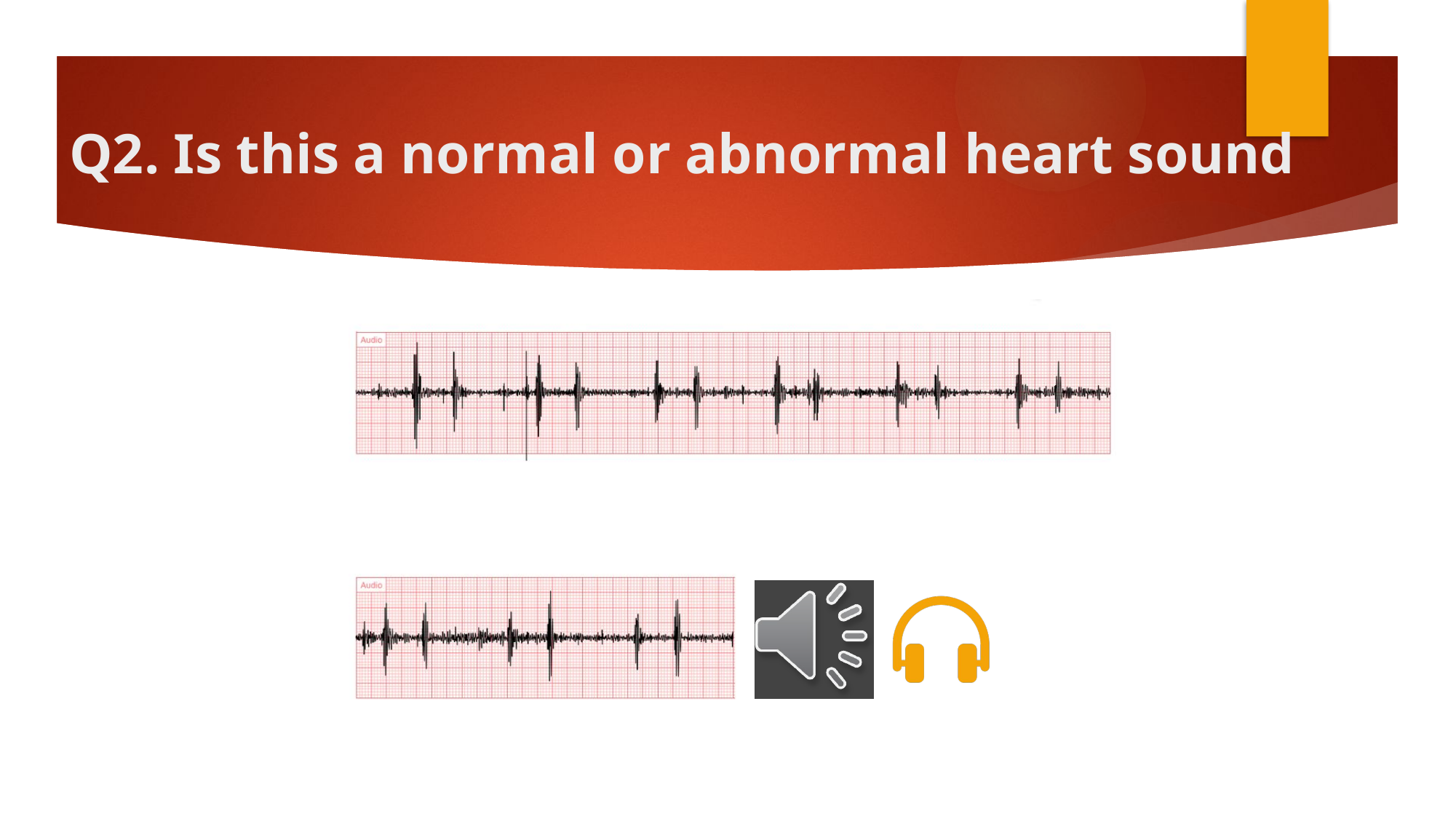

# Q2. Is this a normal or abnormal heart sound

## Slide 62
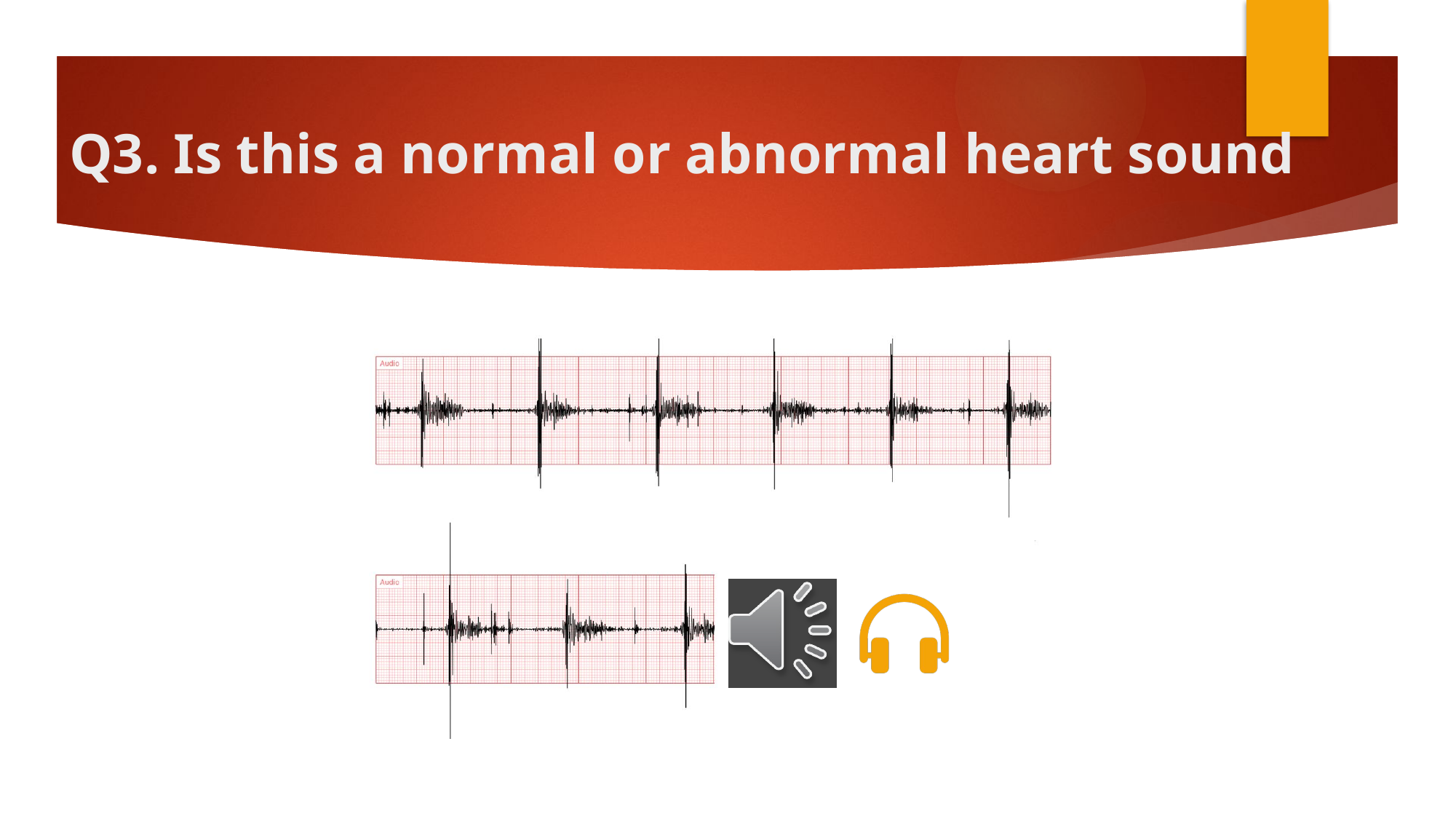

# Q3. Is this a normal or abnormal heart sound

## Slide 63
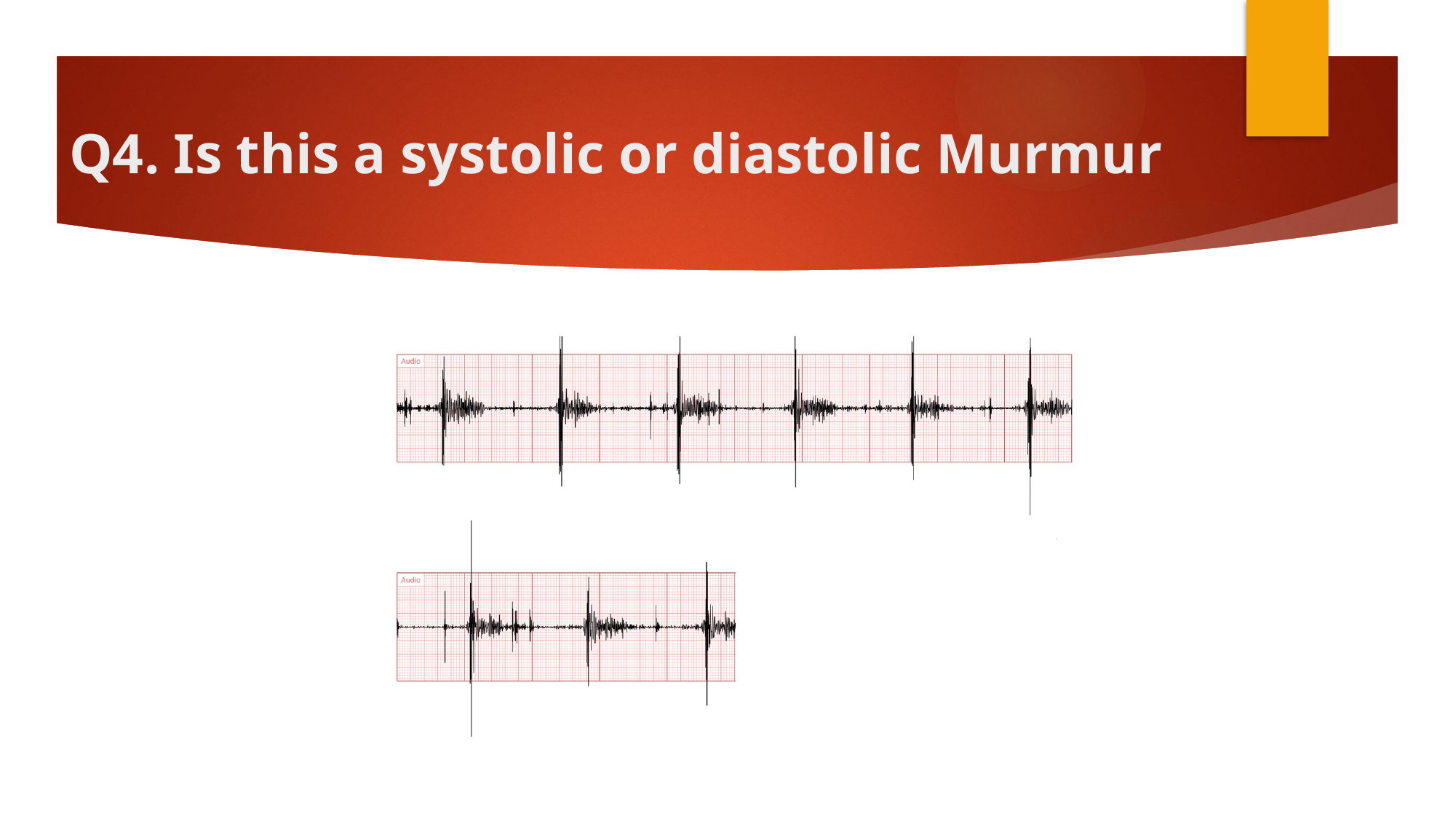

Q4. Is this a systolic or diastolic Murmur

## Slide 64
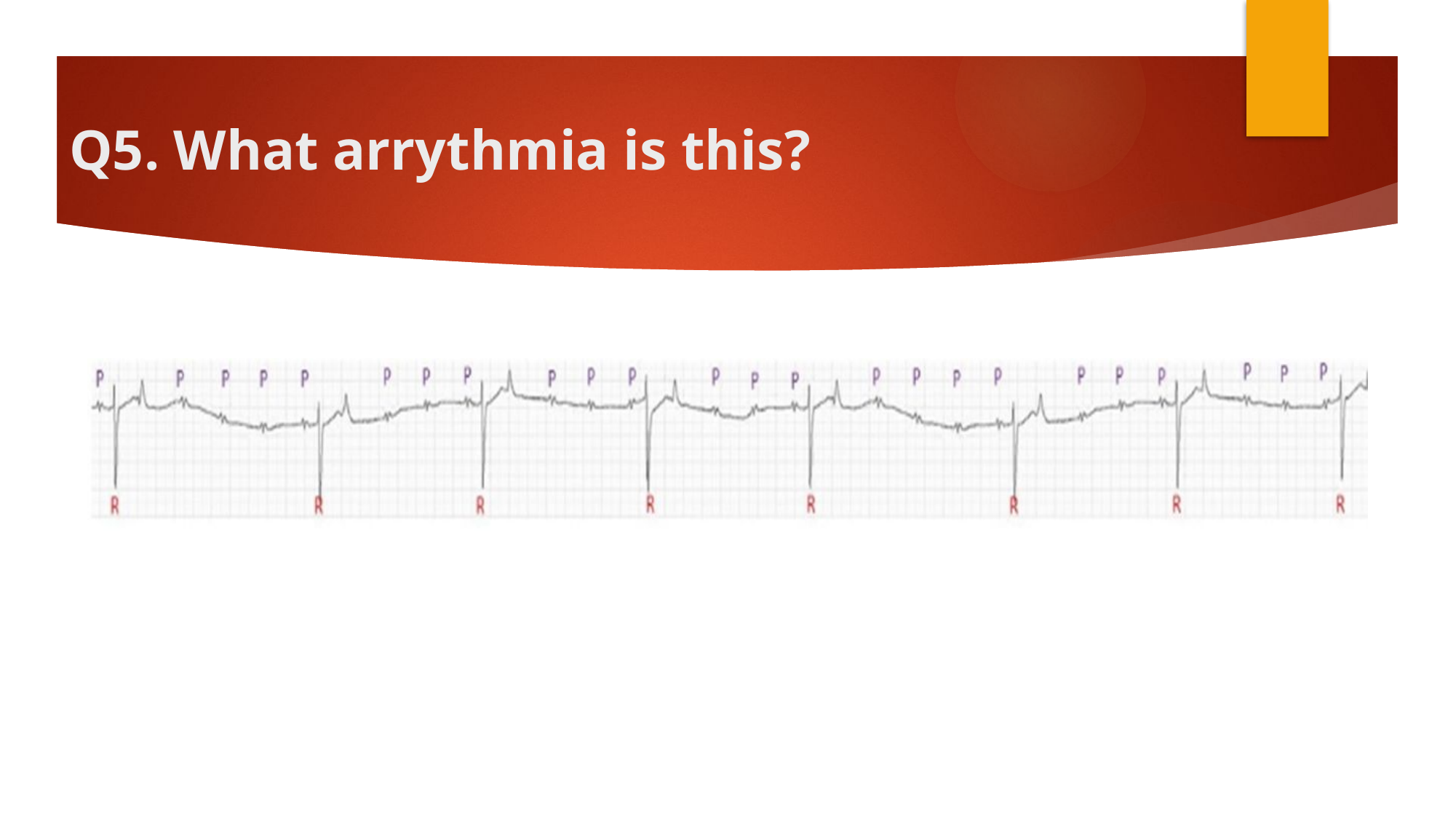

# Q5. What arrythmia is this?

## Slide 65
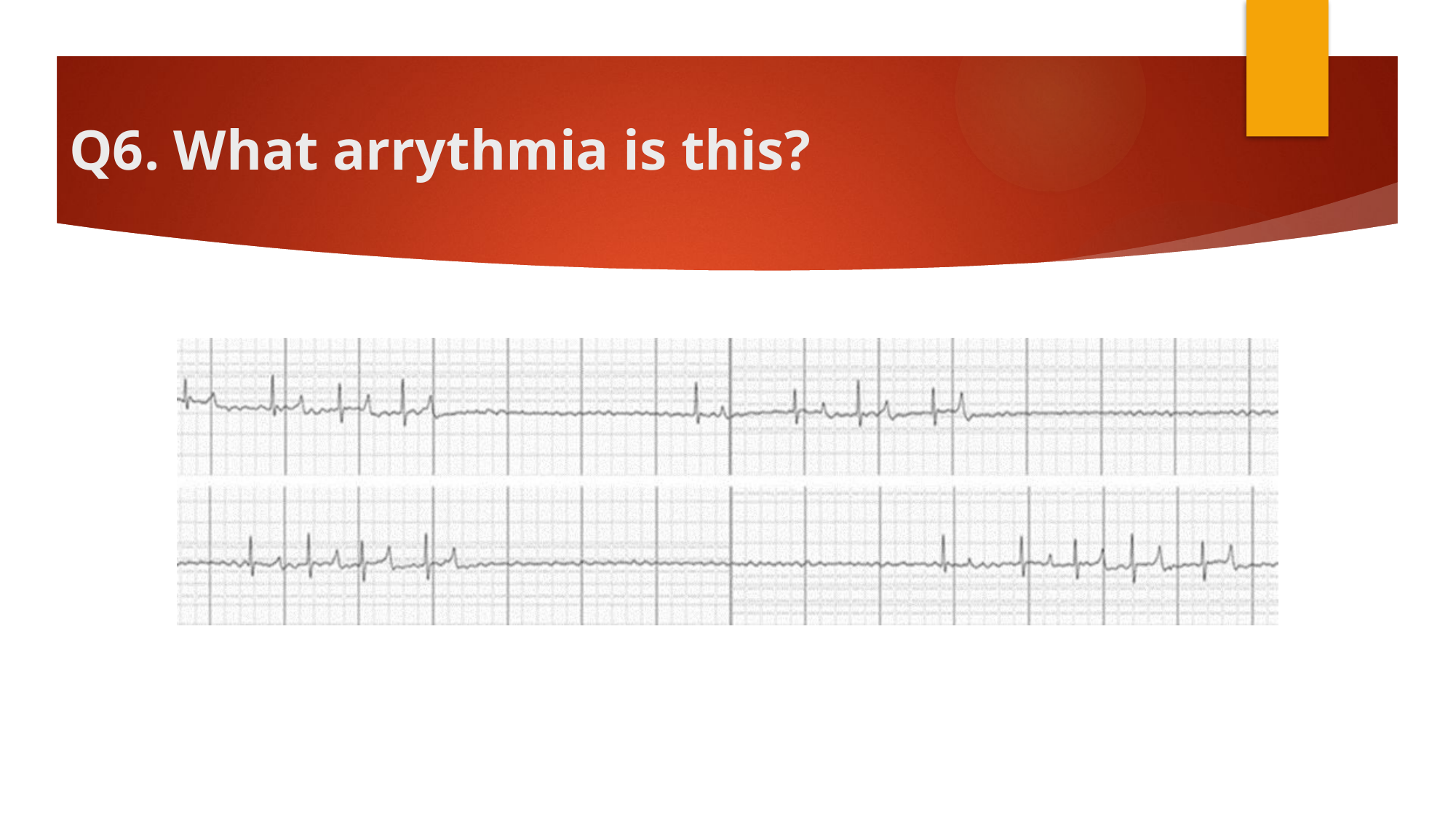

Q6. What arrythmia is this?

## Slide 66
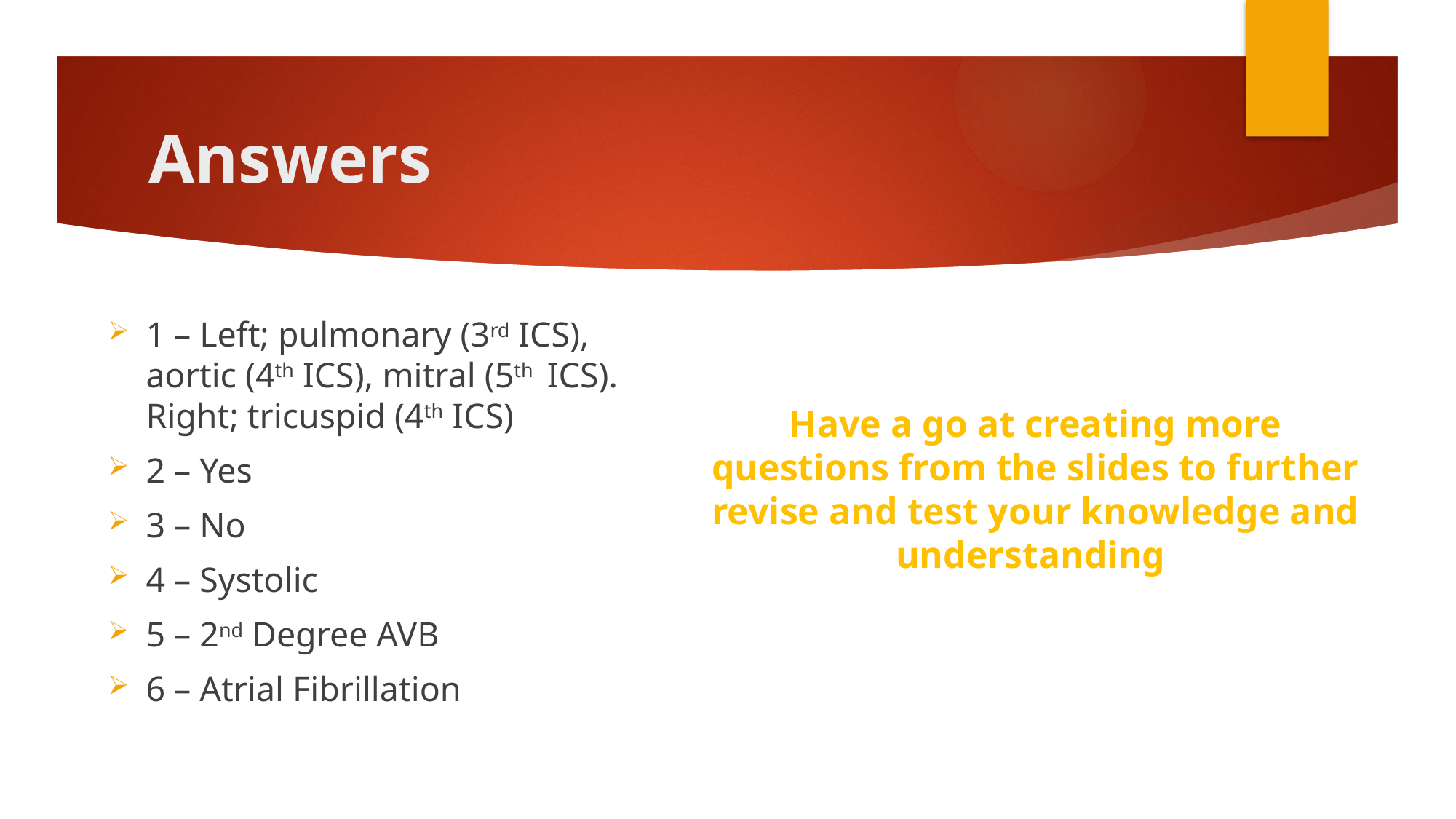

# Answers
1 – Left; pulmonary (3rd ICS), aortic (4th ICS), mitral (5th ICS). Right; tricuspid (4th ICS)
2 – Yes
3 – No
4 – Systolic
5 – 2nd Degree AVB
6 – Atrial Fibrillation
Have a go at creating more questions from the slides to further revise and test your knowledge and understanding

## Slide 67
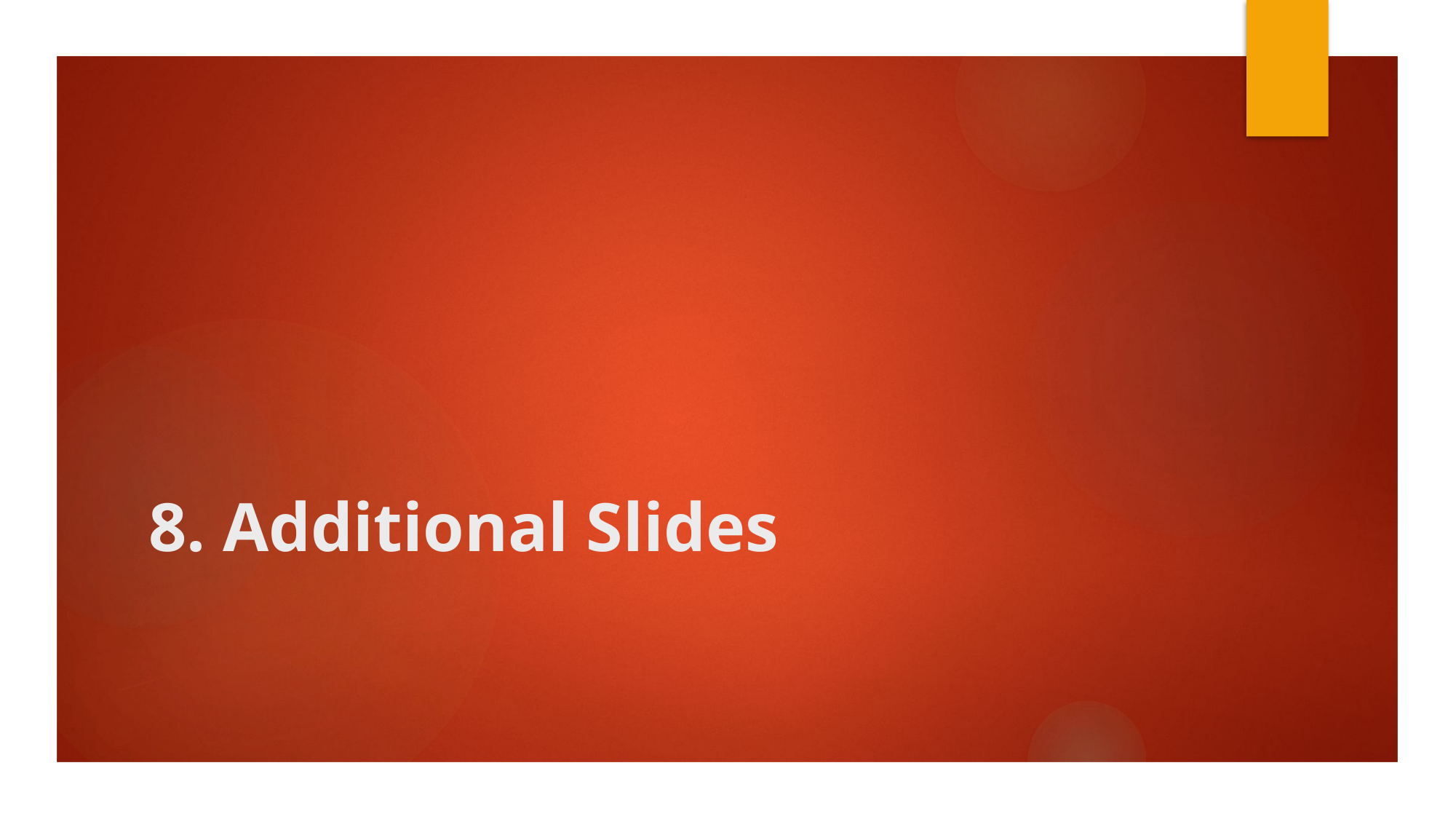

# 8. Additional Slides

## Slide 68
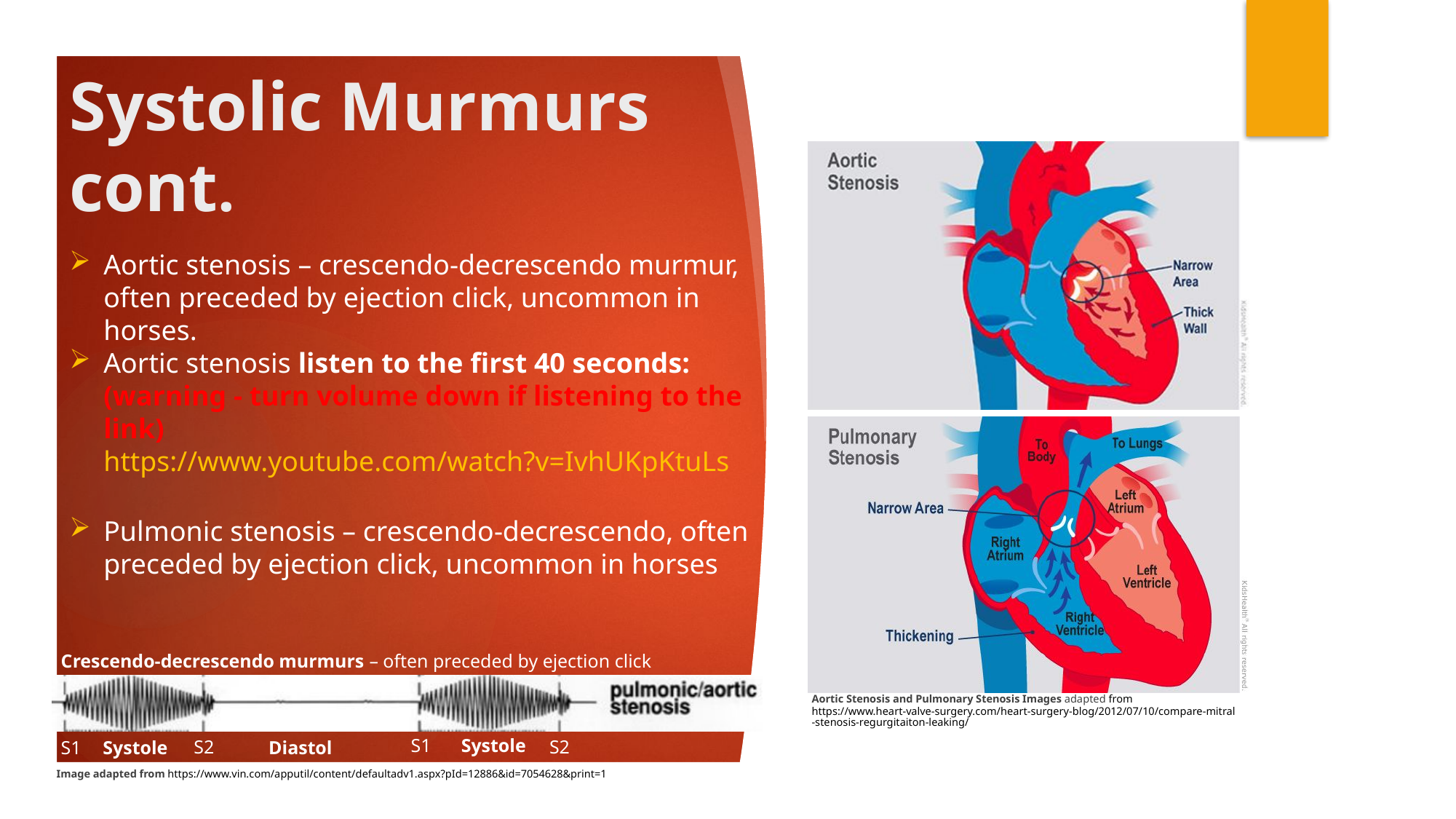

# Systolic Murmurs cont.
Aortic stenosis – crescendo-decrescendo murmur, often preceded by ejection click, uncommon in horses.
Aortic stenosis listen to the first 40 seconds: (warning - turn volume down if listening to the link) https://www.youtube.com/watch?v=IvhUKpKtuLs
Pulmonic stenosis – crescendo-decrescendo, often preceded by ejection click, uncommon in horses
Crescendo-decrescendo murmurs – often preceded by ejection click
Aortic Stenosis and Pulmonary Stenosis Images adapted from https://www.heart-valve-surgery.com/heart-surgery-blog/2012/07/10/compare-mitral-stenosis-regurgitaiton-leaking/
S1
Systole
S2
S2
S1
Systole
Diastole
Image adapted from https://www.vin.com/apputil/content/defaultadv1.aspx?pId=12886&id=7054628&print=1

## Slide 69
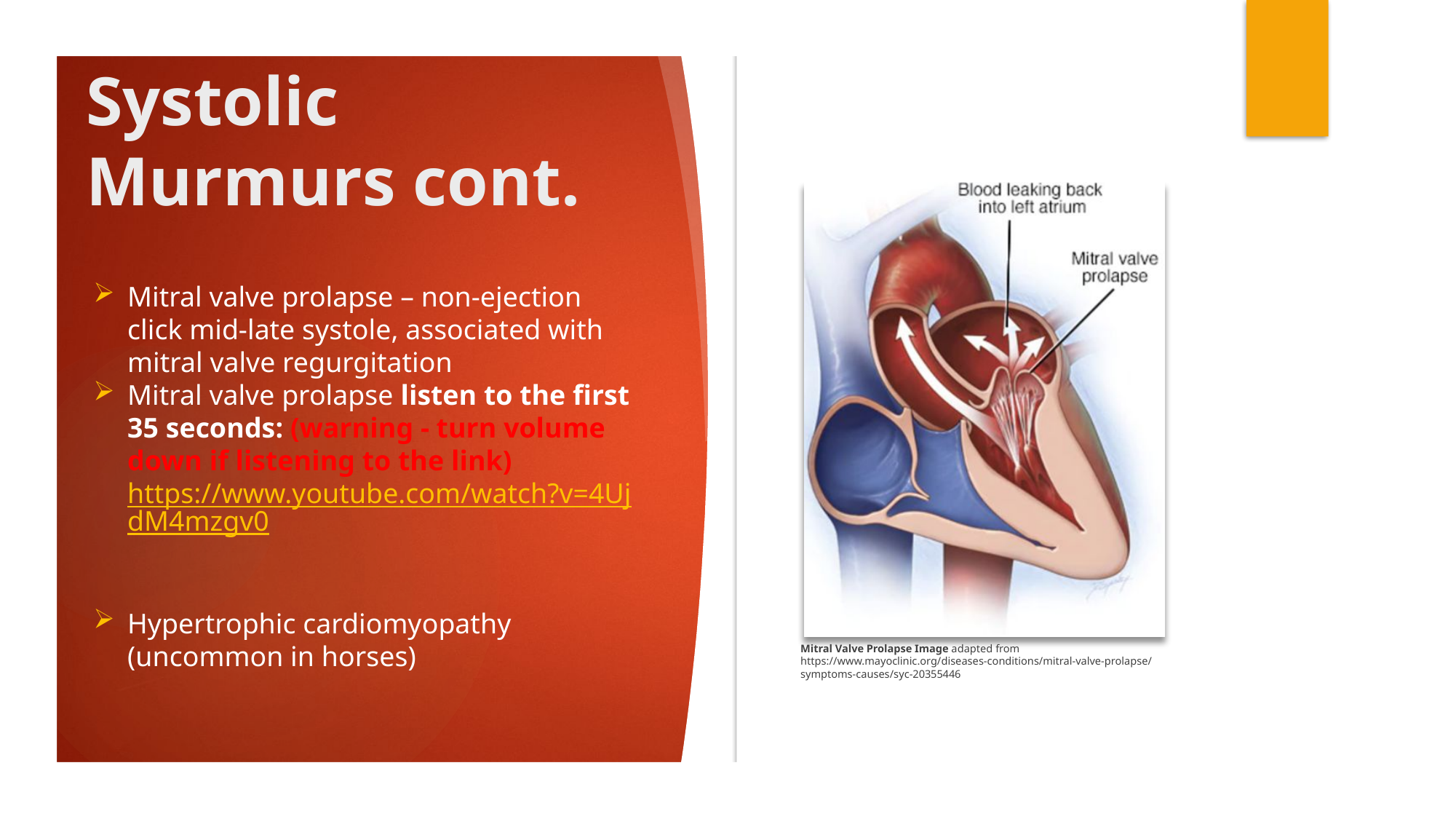

# Systolic Murmurs cont.
Mitral valve prolapse – non-ejection click mid-late systole, associated with mitral valve regurgitation
Mitral valve prolapse listen to the first 35 seconds: (warning - turn volume down if listening to the link) https://www.youtube.com/watch?v=4UjdM4mzgv0
Hypertrophic cardiomyopathy (uncommon in horses)
Mitral Valve Prolapse Image adapted from https://www.mayoclinic.org/diseases-conditions/mitral-valve-prolapse/symptoms-causes/syc-20355446

## Slide 70
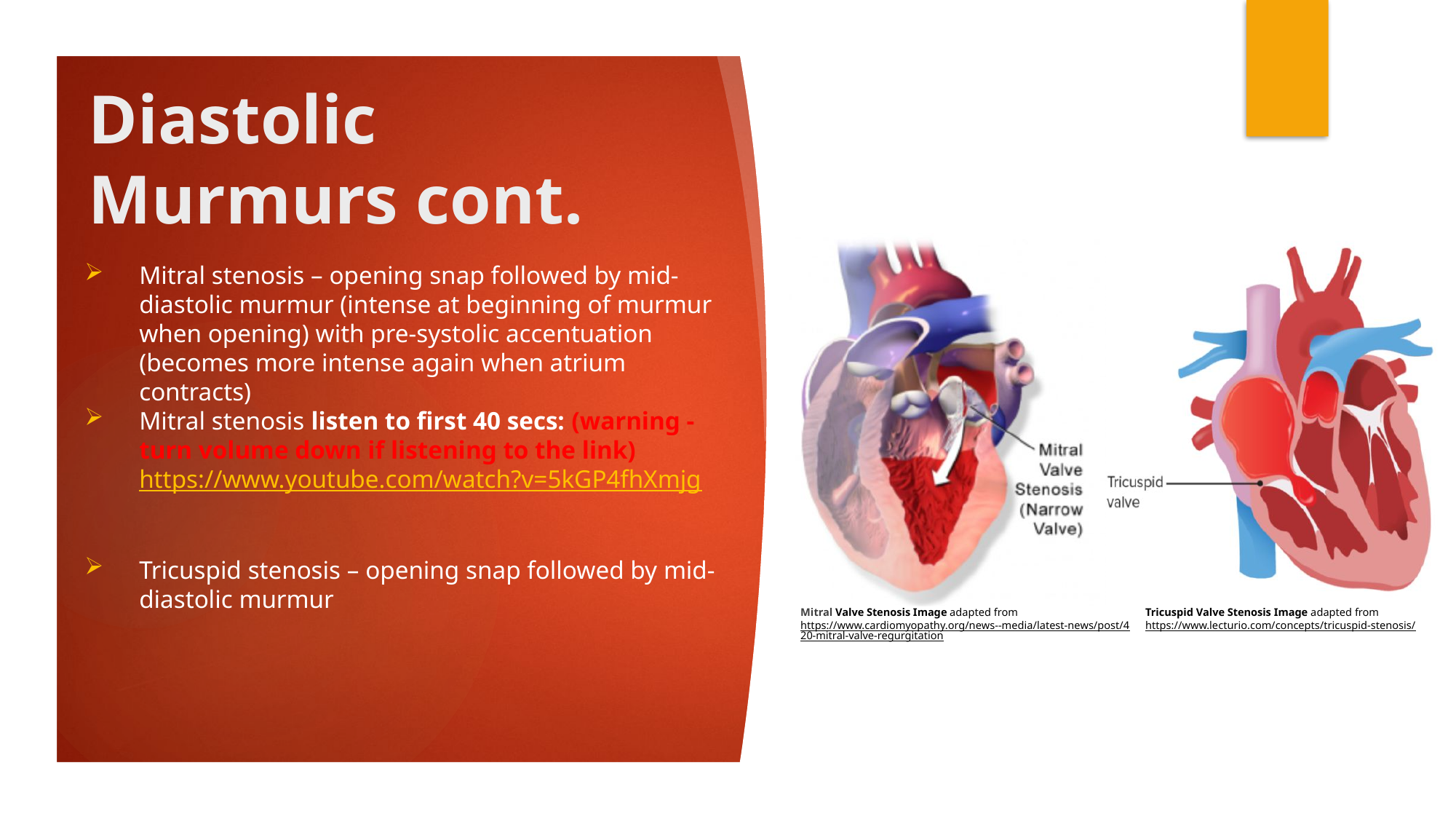

Diastolic Murmurs cont.
Mitral stenosis – opening snap followed by mid-diastolic murmur (intense at beginning of murmur when opening) with pre-systolic accentuation (becomes more intense again when atrium contracts)
Mitral stenosis listen to first 40 secs: (warning - turn volume down if listening to the link) https://www.youtube.com/watch?v=5kGP4fhXmjg
Tricuspid stenosis – opening snap followed by mid-diastolic murmur
Mitral Valve Stenosis Image adapted from https://www.cardiomyopathy.org/news--media/latest-news/post/420-mitral-valve-regurgitation
Tricuspid Valve Stenosis Image adapted from https://www.lecturio.com/concepts/tricuspid-stenosis/

## Slide 71
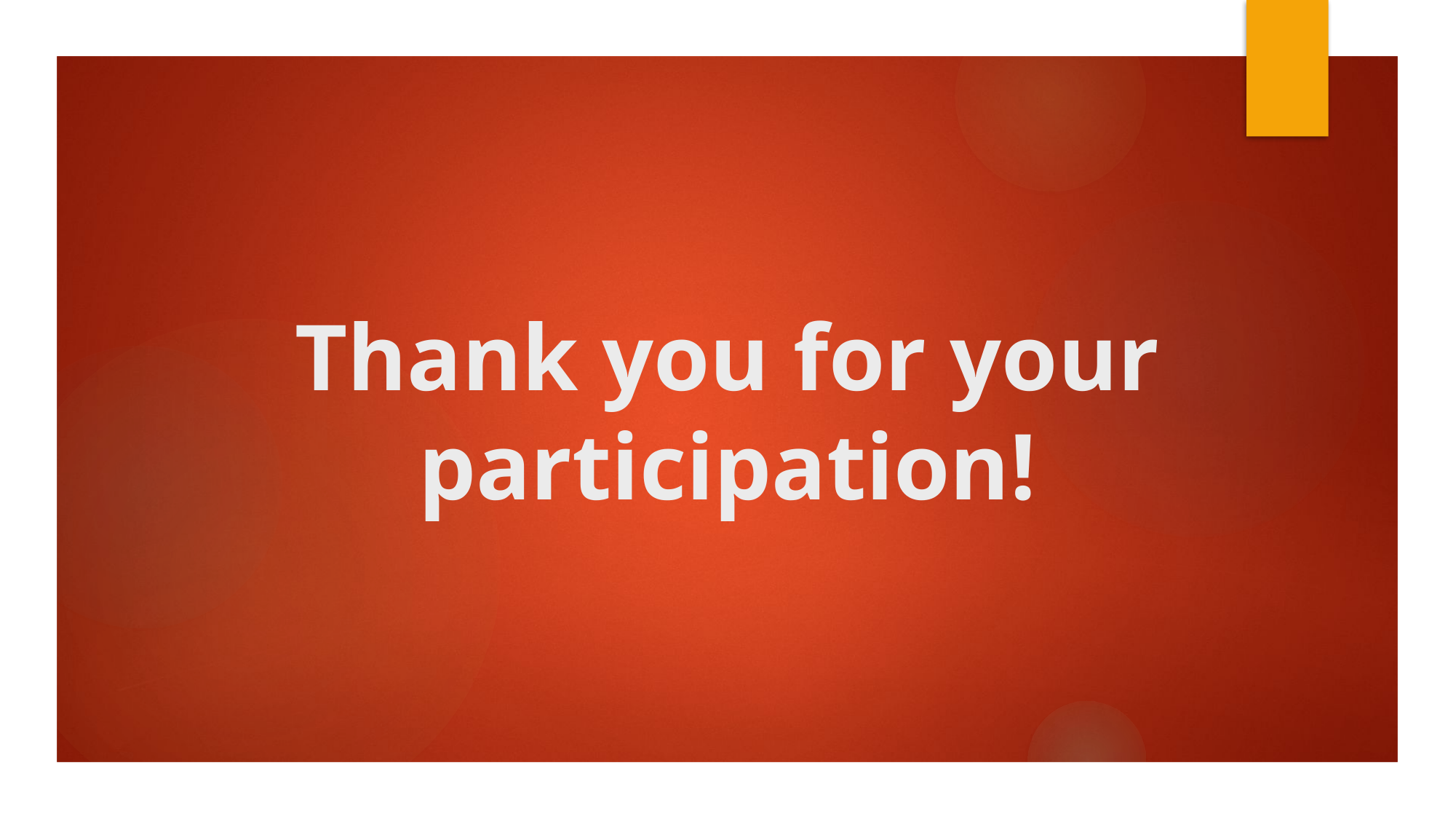

# Thank you for your participation!
