# Supplementary material for: Assessment of a Teaching Module for Cardiac Auscultation of Horses by Veterinary Students
Source: Animals (Basel). 2024 Apr 29;14(9):1341. doi: 10.3390/ani14091341 (PMC11083587; doi:10.3390/ani14091341)
Supplement: Supplementary file 1 [file animals-14-01341-s001.zip › File S2. Preintervention Survey (students & veterinarians) Prior to access to learning resource.pdf]

Pre-intervention Survey (students & veterinarians): Prior to access to learning resource

- I have read the survey information sheet attached and consent to my survey data being used for research

  yes ☐

- Which of the following captures your age (in years)?
  - Less than 20
  - 21-25
  - 25-30
  - 30-35
  - 35-40
  - 40-50
  - 50-60
  - Above 60
- What is your highest level of education before you commenced BVSc (Hons)?
  - Year 12
  - Certificate IV
  - Bachelor degree (with or without Honours)
  - Master degree
  - Doctoral degree
  - Other (please specify): ______________
- If you're a student, how many years are you into your veterinary science study?
  - 3rd year
  - 4th year
  - 5th year
  - First year out
  - Undertaking residency

- N/A

- If you’re a veterinarian, how many years is it since you’ve graduated?
    - 1-5 years
    - 5-10 years
    - >10 years
    - N/A

Please indicate how strongly you agree or disagree with each of the following statements.

Note: *Statements refer to common auscultatory findings in horses*

	Strongly agree	Agree	Neutral	Disagree	Strongly disagree
Q1. I am interested in more audio-visual learning resources for cardiac auscultation					
Q2. I think these resources would benefit practical studies immensely					
Q3. I would like to see more resources with recordings of GIT and lung sounds also					
Q4. I/students would feel more competent in practicals if we/they had access to such learning resources beforehand					
Q5. I am already competent at auscultating sounds over each of the heart valves					

Q6. I am familiar with identifying normal heart sounds					
Q7. I am able to identify mitral valve regurgitation (systolic murmur)					
Q8. I am able to identify aortic valve regurgitation (diastolic murmur)					
Q9. I am able to identify second degree atrioventricular (AV) blocks					
Q10. I am able to identify atrial fibrillation					
Q11. I am able to identify ventricular septal defects					
Q12. I am able to identify a patent ductus arteriosus					
Q13. I am able to identify pentology or tetralogy of fallot					
Please write your response in the spaces below:					
What practical skills are you most interested in learning/teaching more about regarding auscultation?					
How would you describe your competency level with auscultation of the heart, lung and GIT.					

What do you find most difficult about developing auscultation skills	

Is there anything that you would like to tell us? ____

So we can link your responses before and after using the learning resources, we need to create a Password for the next survey:

What is the name of your first pet? ________________

What is the name of the street you grew up on? ________________

If you no longer wish to participate in this survey, simply close the browser window without selecting Submit.

Copy of survey results?

If you would like to be sent a copy of the survey results, please click the link below. This will take you to another webform where you can enter your contact details (as seen below). This will ensure that your survey responses will remain anonymous.

Contact information:

Name: ________________

UQ student number: ________________

UQ email address: ________________

Thank you very much for your time and feedback!
