# Supplementary material for: Assessment of a Teaching Module for Cardiac Auscultation of Horses by Veterinary Students
Source: Animals (Basel). 2024 Apr 29;14(9):1341. doi: 10.3390/ani14091341 (PMC11083587; doi:10.3390/ani14091341)
Supplement: Supplementary file 1 [file animals-14-01341-s001.zip › File S3. Postintervention Survey (students) After access to learning resource.pdf]

Post-intervention Survey (students): After access to learning resource

I have read the survey information sheet attached and consent to my survey data being used for research

yes ☐

So we can link your responses before and after using the learning resources, can you please enter to following two questions as you did on the first survey

What is the name of your first pet? ________________

What is the name of the street you grew up on? ________________

-

- Which of the following captures your age (in years)?
  - Less than 20
  - 21-25
  - 25-30
  - 30-35
  - 35-40
  - 40-50
  - 50-60
  - Above 60
- What is your highest level of education before you commenced BVSc (Hons)?
  - Year 12
  - Certificate IV
  - Bachelor degree (with or without Honours)
  - Master degree
  - Doctoral degree
  - Other (please specify): ______________
- If you’re a student, how many years are you into your veterinary science study?

- 3rd year
- 4th year
- 5th year
- First year out
- Undertaking residency
- N/A

- If you're a veterinarian, how many years is it since you've graduated?
  - 1-5 years
  - 5-10 years
  - >10 years
  - N/A

Please indicate how strongly you agree or disagree with each of the following statements.

Note: *Statements refer to common auscultatory findings in* *horses*

	Strongly Agree	Agree	Neutral	Disagree	Strongly disagree
Q1. This resource helped me prepare for practicals/training					
Q2. The resource was easy to work with					
Q3. I prefer clear high-quality audio without background noise and artefacts for learning new auscultation techniques,					
Q4. I feel more competent with my practical and diagnostic skills					

Q5. I can differentiate between normal and abnormal equine heart sounds					
Q6. I believe I can diagnose heart sounds with more accuracy after using the resource					
Q7. I am familiar with identifying normal heart sounds					
Q8. I am able to identify mitral valve regurgitation (systolic murmur)					
Q9. I am able to identify aortic valve regurgitation (diastolic murmur)					
Q10. I am able to identify second degree atrioventricular (AV) blocks					
Q11. I am able to identify atrial fibrillation					
Q12. I am able to identify ventricular septal defects					
Q13. I am able to identify a patent ductus arteriosus					
Q14. I am able to identify pentology or tetralogy of fallot					

Open text:

What was most helpful about the learning resource:

What would improve the learning resource?

If you no longer wish to participate in this survey, simply close the browser window without selecting Submit.

Copy of survey results?

If you would like to be sent a copy of the survey results, please click the link below. This will take you to another webform where you can write your contact details. This will ensure that your survey responses will remain anonymous.

Contact information:

Name: ________________

UQ student number: ________________

UQ email address: ________________

Thank you very much for your time and feedback!
