# Supplementary material for: Assessment of a Teaching Module for Cardiac Auscultation of Horses by Veterinary Students
Source: Animals (Basel). 2024 Apr 29;14(9):1341. doi: 10.3390/ani14091341 (PMC11083587; doi:10.3390/ani14091341)
Supplement: Supplementary file 1 [file animals-14-01341-s001.zip › File S4. Thematic analysis pre- and pos-intervention questions.pdf]

Thematic analysis – Coding and Q's

Pre-intervention Analysis

**Definitions of Coding Nodes – 'What practical skills are you most interested in learning/teaching more about regarding auscultation?'**		
**TITLE**	**CODE**	**ATTRIBUTES**
Competency	COMP	Any reference to competency in auscultating the heart
Abnormal versus normal	A&N	Any reference to distinguishing between normal and abnormal
Practical skills	SKILL	Any reference to developing practical skills and/or training
Anatomy & Physiology	A&P	Any reference to anatomy and physiology
Heart murmurs & arrythmias	M&A	Any reference to heart murmurs and arrythmias
Lung or GIT	L/G	Any reference to clear explanations and descriptions
Other	OTH	Anything that doesn't fit any of the above!

**Definitions of Coding Nodes – 'How would you describe you competency level with auscultation of the heart, lung and GIT?'**		
**TITLE**	**CODE**	**ATTRIBUTES**
Good	G	Anything relating to a good level of competency when auscultating
Average	A	Anything relating to a moderate/average level of competency auscultating
Poor	P	Anything relating to a poor level of competency auscultating

**Definitions of Coding Nodes – 'What do you find most difficult about developing auscultation skills?'**		
**TITLE**	**CODE**	**ATTRIBUTES**
Practical skills	PRAC	Any reference to the lack of opportunity to gain practical skills in auscultation
Abnormal versus normal	A&N	Any reference to difficulty in distinguishing between abnormal pathologies and normal to make a diagnosis
Anatomy and physiology	A&P	Any reference to understanding anatomy, physiology and placement of stethoscope for auscultation
Heart murmur	MUR	Any reference to heart murmurs specifically subtle/low grade heart murmurs
Teaching and learning	TEA	Any reference to having supervision when auscultating, teachers or learning resources
GIT auscultation	GIT	Any reference to ease of use
Other	OTH	Anything that doesn't fit any of the above!

**5th Year Survey: Pre-Cardiology Learning Resource**			
**2021**			
**ID**	**What practical skills are you most interested in learning/teaching more about regarding auscultation?**	**How would you describe your competency level with auscultation of the heart, lung and GIT?**	**What do you find most difficult about developing auscultation skills?**

1 (discard)	Diagnosing murmurs	Good	Diagnosing low grade murmurs
2 (discard)	Knowing normal	Fair to Good	Hearing normal, hearing in field
3 (discard)	Competent auscultation of heart	Average	Describing what I hear. Deducing what is abnormal.
4 (discard)	Everything. Initially what is normal and then how to distinguish different parts of the heart working (and other organs) and then learn to identify common abnormalities. I am in second year so we have not done very much of this yet.	Not competent yet	Starting- just got stethoscope today, would be good to know what is common in humans and animals in terms of sounds so we can practice on ourselves firstXD
5	What different conditions sound like on auscultation	Basic	Describing the patterns I hear upon auscultation
6	Normal and abnormal lung sounds	Very poor	Difficult to practice at home
7	How to identify what type of murmur	Competent with Identifying normal and abnormal but not identifying exact types of murmurs	Exposure
8	Identifying murmurs and arrhythmias	Moderate level; able but not confident	Having enough resources to consistently compare normal to abnormal.
9	Identifying murmurs	Poor	Understanding what the different components of a heart beat mean (i.e. systolic, diastolic) and what murmurs mean (i.e. crescendo, decrescendo) and I also do not understand how to read and ECG
10	Experience hearing different murmurs	Average	Not many opportunities to hear abnormalities
11	Identify murmur	Poor	Lack of experience
12	Heart and GIT auscultation	Average	Knowing what normal GIT sounds are and identifying heart murmurs
13	Hands on experience with diagnosed cases of cardiac murmurs and arrhythmias.	It is okay, would like to hear more real life cases of abnormal lung and heart sounds. Would not be able to identify increased GIT sounds	Hearing low grade heart murmurs, developing confidence to confidently identify mildly abnormal changes
14	Just a basic understanding of how to do it with good technique and the most common findings.	Very poor	Placement of the stethoscope and understanding what you are trying to hear

	Especially want to know 'normal'		
15	I need more training that focuses on the practical application. I struggle to understand a lot of the cardiac training when it is just in lecture form (and we did not have any pracs on clinical exam of the horse or cardiac auscultation due to COVID)	I know roughly where to place the stethoscope, but I have trouble identifying problems	The lack of practical training in this area is extremely lacking in this program.
16	Learning to differentiate the different heart sounds	Non-existent	Lack of ability to practice on actual animals
17	Real world practice classifying and grading murmurs	Satisfactory. I can hear a grade 3+ murmur, can usually describe it as systolic or diastolic. Can identify harsh bronchovesicular, harsh crackles and wheezes. Can identify presence and intensity of GIT sounds.	Lack of hands on experience, we learn so much theory but I had probably heard 8 murmurs before 5th year. I have definitely improved during my clinical time.
18	Identifying heart conditions	Not adequate	Not getting familiar enough with the abnormal
19	Lung sounds - I find it really difficult	Proficient but not great	Lack of practice in the field. I find it very different auscultating real patients because they are all different and there are lots of background noises, in comparison to online library
20	Abnormal heart and lung sounds	Moderate for GIT and heart; poor for lung	Lack of resources or ability to auscultate an abnormal sounds and have a person with expertise confirm whether or not I was correct in my assessment.
21	Classifying murmurs	Sound, but have not been exposed to certain types of pathology. Audio-learning would give me more exposure so that I can recognize the abnormality later.	Recognizing abnormalities that you have never heard before.
22	Picking valves and systole/diastole	Improving, confident to pick up obvious murmurs	Gaining the experience/case load to listen to
23	How to identify normal and abnormal heart sounds and the topographical anatomy to find such sounds/heart valves	No very competent	Finding the anatomical locations to hear heart sounds

24	Identifying murmurs	Beginner-intermediate	Getting enough practice
25	How to recognise abnormalities efficiently	Needs improvement	The opportunity to actually practice on an animal
26	Cardiac and GI auscultation	Low level competency	Gaining practical experience
27	Exposure to real life murmurs / regurgitates that aren't so obvious coz that's realistic	Not bad but we weren't able to do clinical exams in third year due to covid so technically never was taught how to do this on the horse	Sometimes struggle to find the actual heart but once I do I'm gokd
28	Just practicing it more so i can identify abnormalities better and be confident in doing it	Low competency. Good in theory but havent actually done practicals to be competent	I guess more the availability of horses to practice
29	Listening to the actual horses with cardio abnormality	Normal	Differentiating the different murmurs
30	I want to learn the basics for day 1 competencies	Incompetent. I have never auscultated on a horse before. I may be able to identify some stuff by matching it to previous recordings I have heard.	Where to put the stethescope to get all the desired valves. I always have difficulty finding stuff, so I think I will have difficulty finding the valves.
31	Where to auscultate	Not competent	Lack of practice
32	Being able to differentiate normal vs abnormal heart/lung/gut sounds	Not very competent	Not hearing enough normal sounds to be able to differentiate from abnormal sounds
33	Detecting murmurs and arrhythmias	Average	Hearing the subtle heart sounds of a murmur
34	.	At this point in learning, my competency is quite low	When you are in a prac it is hard to know you are hearing exactly what the tutor is hearing
35	Performing more precise cardiac auscultation	not very good	Not having enough practical hours to develop skills
36	Auscultation	Very low	Putting lectures into real life
37	Being more adept at picking up abnormal sounds and their timings	Low	Lack of auditory practice combined with physical placement of stethoscope on an actual horse
38	The different heart problem sounds	Under confident	Having limited chances to practice
39	Correct sounds	Not competent	Not knowing what to be listening for
40	Familiarising myself with common abnormalities	Missed a lot of 3rd year thanks to covid…. So realistically it could use some work	Some individuals are harder to hear than others and when you're learning it can be off-putting/difficult to tell if you're placing your

			stethoscope in the right place or if you need to push down harder etc.
41	Diagnosing abnormal heart sounds	Poor, experience only in first year	Learning the right spots to auscultatate
42	Identifying pathology in specific valves	I can find the correct locations and listen but not sure of exact pathology sounds	Getting experience listening to different species with different pathologies
43	Proper placement of the stethoscope	Poor	Having the opportunity to practice
44	Heart sounds	Poor	Lack of opportunities to practice
45	More understanding of normal heart sounds	Very low	Not enough practical time
46	Identifying murmurs and other heart issues	Very low	We don't have enough practicals to practice doing them
47	Anatomical landmarks	poor	Anatomical landmarks and lack of access to live animals
48	Where to place stethoscope in each animal. What does normal sound like. What should I when I can't hear something when I should be able to with the stethoscope I have?	Low - I have attempted to practise this on my cats (only practice animals available to me), but it's more of a 'stab in the dark' approach rather than useful building of skills	We did not do any PE practicals on anything but cattle. I feel like I struggle to find the proper landmarks or perhaps do not have the proper technique for my auscultation to be of diagnostic quality.
49	Mainly normal vs abnormal sounds	Extremely limited	Knowing the normal ranges initially
50	Heart sounds, specific locations to identify specific pathologies and getting familiar with the different sounds of different pathologies	I feel confident in auscultating normal in the heart, lungs and GIT and so may be able to identify abnormal but not specific diagnosis, especially heart sounds. Lung abnormalities are easier to classify as crackles, wheezes, etc.	You don't really get to hear an abnormality until it is presented to you, so I only know what normal sounds like so I may be able to identify abnormal but not specific diagnosis.
51	Positioning of the stethoscope for each valve	Capable in identifying normal and can identify when things are abnormal but poorly specific for any particular cardiac disease	Translating a description of a pathology into listening and diagnosing said pathology
52	The differences in murmurs	Poor	Noticing the very subtle murmurs
53	How to determine what type of murmur there is, some can be so subtle I have no idea how tell if	Very low - especially in horses	Hearing if there is something wrong because it can be so subtle and then determining the location

	there is one and what sort it is		
54	Where to actually listen	In horses, limited (in smallies and cattle competent)	Lack of practice on real horses
55	Identifying abnormal sound	Basic	Practical time to practice
56	Finding correct positioning of stethoscope, and differentiating the different types of murmurs (still get mixed up between them)	Not very competant at all. I am confident I can identify 'normal' in most cases, but then again, I still lack in a lot of experience.	Unless I can hear something, and be told/figure out - what I'm hearing is 'this', then it's tricky to develop an ear for it. I think the more practice I can get, the better (especially with a teacher to help avoid confusion/incorrect reasoning).
**2022**			
57	Identifying any cardiac conditions	Very poor	Hearing a pattern and actually remembering what condition it identifies to
58	Murmurs	poor	Connecting the sound to the condition and its pathogenicity
59	Getting exposed to more normal cases so I can identify when something is abnormal and then go from there.	My skills are pretty basic, I can auscultate normal reasonably comfortably, I think I would be able to identify abnormal sounds and describe them with basic terms but wouldn't be able to identify specific abnormalities yet.	Getting enough exposure to normal and abnormal cases to actually hear the difference.
60	Segregating systole and diastole	Okay at identifying normal, not so good at abnormal	Murmurs
61	All the pathology regarding cardiac	Probably 0/10 at the moment, I can only pick up sounds and that is all for me for now.	I even have the difficulty to put the stethoscope at the right location to pick up the normal sound....Sometimes i can get very loud heart beat while sometimes i do not pick up a single sound.
62	Where to find each valve on the horse. I can find the mitral valve easily the others not so well. Also, the ability to recognise the most common abnormalities on auscultation.	Just enough to be considered competent	Where to place the stethoscope-cardiac
63	Arrhythmia/murmur identification	Moderate - I can identify when something is	Describing abnormalities.

		abnormal, but often not the specific abnormality.	
64	Identifying valves	Neutral	Don't know what the normal/abnormal sounds supposed to sound like
65	How to localise murmurs and grade them	I can identify normal heart sounds and abnormal, but not diagnose specific murmurs. Happy with identifying normal bronchovesicular lung sounds and GIT sounds in horses.	Most of the animals we encounter during our time in vet school are clinically "healthy", so it's hard to practice listening to abnormal heart, lung and GIT sounds.
66	Grading murmur	Low	Capturing subtle differences
67 (discard)	Identifying murmurs	Basic	Time to practice on different horses
68	GIT and lameness tutorials similar to this would be great	It was oK RE lungs and GIT, and OK with the heart, definitely better after Alyses tutorial tho	It can be so subtle - and sound so different over the different valves
69	How to identify murmurs and arrhythmias	Able to identify abnormalities but unable to classify them	Lack of opportunity to practice
70	Auscultating different murmurs	Poor	Having an reference for what is normal
71	Identifying murmurs	Poor	Not having someone confirm whether what I hear is correct
72	Learning about the different murmurs	I can usually pick up most things, but do not feel confident enough to diagnose Something without having someone else double check	Getting enough practice with being able to double check with someone with mroe experience
73	Being able to differentiate different heart murmurs	Poor	Being able to know what is normal vs abnormal
74	ID different sounds	Not good	Chance to really have a real animal to practice on
75	Ability to identify murmurs and type of murmurs	Moderate	Identifying low grade heart murmurs
76	Lung and heart sounds	Very poor	Difficult to teach and difficult to learn
77	Just further opportunities to listen and practice. I had the equine medical prac yesterday and we listened to only four horses and I found it very challenging	Poor-moderate - I just dont have enough experience	Discerning what is a murmur and what is normal or gut sounds
78	Identifying murmur/abnormalities	Can identify the murmur but can't specify the type	Not having enough experience (only theory based)
79	What the characteristic sounds are of typical	I feel more competent calling lung and gut	I struggle to differentiate the different valves and to

	cardiac abnormalities so I feel more confident in recognising them.	sounds normal than I do cardiac sounds as I believe there is much more variation with these sounds.	note what rhythms are abnormal.
80	Using echocardiogram to identify AV blocks etc, preferably in practical classes instead of just lectures	not very competent, only what we have learnt in 3rd year	Finding the right place to listen every time, especially when animals have individual differences
81	Being able to identify the type of murmur	i know what normal is but struggle with abdnormal	Not having enough practice of abnormal
82	Learning to identify what is abnormal confidently	Very little practice with auscultation	Knowing what is normal and variations of normal
83	The ability to recognise and diagnose abnormalities	poor. have not been exposed enough to be confident in applying theoretical knowledge.	Access to animals with pathology, or high quality audio/visual aids that are realistic and engaging.
84	Listening to non-pathologic vs pathologic sounds	low-moderate competency	Needing lots of exposure to know what you're hearing
85	Recognising different murmur types and when they occur in the cardiac cycle	Low, only able to detect obvious changes	Not having enough hands on practice auscultating animals
86	How to tell in lung sounds are increased or decreased	preliminary	Knowing when something is normal
87	Everything	ok	Not enough opportunities to practice as a student
88	Where to place the stethoscope with precision for each valve	Average	Getting a good sound and knowing which abnormality I am hearing
89	Learning murmurs	…	Available practical time
90	How to locate the different heart valves through auscultation	Average. I can identify heart, lung and GIT sounds, and I am able to identify if the horse has a murmur or an arrhythmia. But I am not able to distinguish what type of murmur or arrhythmia that I am hearing.	Identifying the differences in the different murmurs.
91	Hearing the different types of murmurs and defects	Not sufficient to identify all abnormalities	Having access to animals with murmurs and having confirmation of the type of murmur these animals have
92	Audible resources	Average, needs improvement	Lack of audible resources
93	Being confident in my auscultation skills	Not that confidence. I second guess myself if theres a murmur or not.	Knowing if I'm hearing what I'm meant to be hearing.
94	Identifying pathologies	Adequate but needs more practice	Exposure to opportunities to perform auscultation

Post-intervention Analysis

Definitions of Coding Nodes – 'What was most helpful about the learning resource?'		
**TITLE**	**CODE**	**ATTRIBUTES**
Complete resource	RES	Any reference to the helpfulness of having the material condensed into a single resource
Audio visual	AV	Any reference to the audio or visuals being helpful or the inclusion of audio recordings and/or ECG's in the resource
Abnormal	ABN	Any reference to including information regarding abnormalities or pathologies
Nothing	NOT	Any reference to the resource not being helpful
Revision	REV	Any reference to anatomy and/or revision
Heart murmur	MUR	Any reference to heart murmurs specifically
Clear explanation	CLE	Any reference to clear explanations and descriptions
Ease of use	EASE	Any reference to ease of use
Understanding	UN	Any reference to understanding information presented in the resource
Other	OTH	Anything that doesn't fit any of the above!

Definitions of Coding Nodes – 'What would improve the learning resource?'		
**TITLE**	**CODE**	**ATTRIBUTES**
Clearer explanations	CLA	Any reference to the explanations and clarity of information in the resource
Audio	AUD	Any reference to the clearness of audio recordings or inclusion of more recordings
Concise	CON	Any reference to improving the conciseness of the slides
Nothing	NOT	Any reference to the resource not needing any improvements
Cases	CAS	Reference to more cases and/or inclusion of more quiz questions.
More interaction	INT	Technology and improved graphics to make it even more interactive
Accessibility	ACC	More accessibility and/or incorporation in more areas it can be available
Additional information	INFO	Any reference to include additional information to include
Other	OTH	Anything that doesn't fit any of the above!

5th Year Survey 2021 & 2022: Post Cardiology Learning Resource		
**Respondent ID**	**What was most helpful about the learning resource**	**What would improve the learning resource?**
	**2021**	
1	It was good to have all the material condensed into 1 resource	I still can't tell the difference for auscultation for a fib and 2nd degree av block. I think some slide titles need to be clarified - for example ""mitral regurgitation - aortic valve"" was at first confusing
2	Audio and visuals	Maybe not so many words on a slide
3	Having audio files and all the information together in one place. It was very clearly structured and written	Less youtube links, occasionally there's too many words on the slide- lose interest after a while. May be better if quiz answers came after each question so it's easier to go back and revise?
4	Nothing	Nothing
5	Examples of each type of abnormality	Slightly clearer sound examples
6	Heart sounds	More cases to practice
7	Sounds are quite clear and clearly states what heart murmur the sound is corresponding to	Nothing. I think this resource with adequate experience in auscultation in actual horses will help with this
8	Being able to hear the heart sounds with slides that explain what is happen and show the ECG along with it. It was also nice to not have lecturers talking over the top of the sound so I can actually listen to it	It would be awesome if there was some way to have a little ball move along the ECG in time with the sound so we can link the sound with the visual (kinda like a little karaoke ball). In the quiz section you asked if the sound was normal or abnormal and then answered with a yes and no instead of normal and abnormal
9	I did like the links to YouTube videos - visual animation is the best way to understand and remember physiology I feel	The audio was not great, but realistic of what you will hear out in the field so that is helpful is some regard because you wont get crisp sounds.
10	The audio examples paired with visual cues	More quiz questions. Maybe at the end of each topic
11	Visual information	Could play the sounds first and then show the ECG (so that way I would not be so reliant on the visual cues and would 'listen harder
12	The audio's were very helpful! and the animations were very good to follow along with	Maybe tips on how to limit artifact noises? Or even audio over to explain the whole ppt? I find listening to someone talk can better my learning

13	The audio recordings accompanied with a detailed description of what you were listening to	Maybe if more videos were embedded into the PowerPoint rather than on YouTube
14	The murmur recordings	Even more murmur recordings i.e. from different horses with different grades
15	Listening to the sounds	Question and answer
16	Being able to have information and recordings of the murmurs all in one place	I would lke it if there were recording of most of the murmurs/arrthymias embedded in the slides
17	Very easy to use, helpful audio	Comparing abnormal v normal sounds regularly to note the difference
18	Revision info to look back on	Not much
19	The explanations and annotations which accompanied the audio	NIL
20	The recorded heart sounds	An ecg of the recorded heart sounds so it is easier to follow
21	Recordings of abnormal sounds	The ECG trace moving as the sound is played/animations pointing to each sound on the ECG as it corresponds with the recording. Mainly for when a background noise was herd, otherwise was easy to follow
22	Recording accompanied by matching ECG	Better recordings of tetralogy of fallout, VSD etc. (but understand this could be difficult)
23	Good quality recordings. Concise clear explanations	Having the heart sounds play as a video alongside the sound
24	The audio was clear and demonstrated sounds at different valves	I can't think of anything
25	The recordings were great quality. Overall was a great learning resource I will keep for the future	N/A it was great
26	The recordings of the heart sounds	Different recordings of the same problem, as every case is different and hearing the same problem on different horses would sound different and would help us to learn them better
27	Audio	Clean up the audio
28	Audio samples	Visual tracking of soundwave to go with audio
29	Realistic sounds with gut sounds in background - useful for in practice to know what these sound like	More audio samples
30	Clear + realistic heart sounds that went for a good number of cardiac cycles	Perhaps have a couple of different horses/ grades for normal and different heart sounds

31	Audio files really good	Perhaps a video of the audio recording one the graph at the same time the recordings are paying
32	The sounds and the explanations which allowed me to understand what I am listening to	I know it is difficult to improve but some of the sounds were very quiet and difficult to hear
33	Overlayed waveform transcripts with the audio files was very useful especially for filtering out background noise	Maybe add small explanations of auscultation noises such as ejection clicks, opening snaps etc.
34	The sound recordings	Not hearing people speaking in the background of some of the recordings
35	The clear way it was laid out- I appreciated the clear diagrams. I will definitely be coming back to this before/during my equine rotation in 2022	Some more information about common clinical presentations with the different heart conditions. This is a great resource!
**2022**		
36	Revision of the anatomy and heart sounds, what each sound corresponded to, as well as seeing the trace and hearing the heart sounds side by side	More recordings of murmurs rather than the youtube
37	The audio	Clear audio, more interactive in the later slides","thank you! amazing resource
38	Hearing the abnormal heart sounds and the reasoning behind them	Making it more known that it's available
39	Audio and heart/valves image	Clearer sounds. Personally the amount of ppt slides put me off a bit because i'm not the biggest fan of sitting down and studying. But the contents are very helpful
40	Recordings embedded within presentation	Having the ecg move as the sound plays
41	The recordings plus the ECG	More recordings maybe (high quality one)
42	Excellently presented and easy to use - very straightforward and useful in the fact that there is a revision section before going into abnormalities	Just a few typos and mislabels - when listening to mitral valve the picture might be labelling aortic valve or vice versa. Would be good in some text heavy slides if there could be a small heart diagram in the corner just reminding where the valves are
43	Audio recording of normal and abnormal heart sounds	…
44	Explanation of the types of murmurs and arrhythmias and what they sound like	More audio options and maybe a quiz at the end to test our skills
45	I really liked the audios - they would have been immensely helpful for my	Maybe just more examples? overall it was great.

	medicine prac but I didn't know about the resource!	
46	Realistic sound with GIT sounds	Youtube videos embeded into powerpoint
47	Detailed presentation, clear audio	I still feel like actually doing it on live animals help more than watching and listening presentations
48	I enjoyed listening to the sounds for each individual abnormality and the quiz at the end made me actual realise what I'd picked up	Nothing, it was very useful
49	The murmur sounds	N/A
50	Hearing real life heart sounds rather than perfect simulations	Integrating the YouTube videos important to watch, considering they were extra, I did not watch them
51	Clear and easy to follow	-
52	Good description of the different causes of murmurs and arrhythmias.	Louder audio recordings. Repeat the audio recording automatically once it finishes. Show corresponding position on ECG as the audio is playing.
53	Having clear heart sounds and good descriptions of the sounds	Regulating the sound between videos
54	Recordings	More recording
55	Audible aspect	Accessibility
56	Simplified and easy to understand the physiology	One for abdomens and lungs?
57	Having a single document where i can hear different heart sounds	The volume differences in audio between recordings and youtube vids
